# Low-temperature alcoholysis as a strategy for polyethylene terephthalate recycling and upcycling

**DOI:** 10.1126/sciadv.aed4780

**Published:** 2026-07-17

**Authors:** Matt J. Price, Adam H. Redfearn, Steven T. G. Street, Benjamin F. Tunley, Susanna R. Harvey, Elliott A. L. Smith, Yuya Watanabe, Joshua C. Worch, Arianna Brandolese, Andrew P. Dove

**Affiliations:** ^1^ School of Chemistry, University of Birmingham, Edgbaston B15 2TT, UK.; ^2^ Department of Chemistry, Virginia Polytechnic Institute and State University, 1040 Drillfield Drive, Blacksburg, VA 24061, USA.

## Abstract

Postconsumer plastic waste has the potential to serve as a valuable feedstock in an integrated circular economy in which it can be interconverted between thermoplastic and thermoset materials. However, the development of cost-effective and sustainable recycling or upcycling methods is often constrained by harsh reaction conditions, limited catalyst performance, and overall low process efficiency. Here, we report the low-temperature (120°C) alcoholysis of polyethylene terephthalate (PET) that allows the application of functional, bioderivable alcohols in the depolymerization process. Terpenoids such as prenol proved highly effective, supporting both efficient depolymerization and repolymerization to PET. The unsaturated terpenolysis products were also used to prepare polymer networks via thiol-ene photochemistry and formulated into photocurable resins suitable for three-dimensional printing using digital light processing technology. The terpenoid-based networks were also able to be efficiently depolymerized, demonstrating the potential for loop-to-loop chemical recycling of PET/terpenoid materials. This work establishes low-temperature alcoholysis of PET as a practical strategy for advancing a broader circular PET-based materials economy.

## Teaser

Alcoholysis of PET using terpenoid alcohols enables loop-to-loop recycling and upcycling between thermosets and thermoplastics.

## INTRODUCTION

Polyethylene terephthalate (PET), a widely used polymer in packaging and textiles, represents a substantial component of persistent global plastic waste, in part as a consequence of its resistance to degradation in the environment (*1*). Over the past 70 years, only a small fraction of the >8 billion tons of plastic that have been produced have been recycled (*1*), and as such, there exists a vast untapped stream of valuable postconsumer waste plastic that could serve as an alternative chemical feedstock platform for new materials synthesis. While mechanical recycling is one of the most common recycling methodologies, chemical and enzymatic recycling to monomer (CRM) has emerged as a promising strategy to depolymerize degraded and contaminated PET waste by breaking it down to its monomers, thereby enabling the production of virgin-like PET in a more circular economy (*2*–*4*).

Beyond CRM, upcycling offers sustainable and value-generating alternatives, particularly for reactive, carbonyl-containing polymers (*5*–*10*). PET upcycling has mainly focused on aminolysis, on account of the superior reaction kinetics and more favorable thermodynamics than transesterification methods with alcohols. Catalyst-free aminolysis of PET has been explored to produce antimicrobial polyionenes (*11*) and to create aminoamide monomers and oligomers for capturing CO_2_ (carbon dioxide) (*12*). Using 1,5,7-triazabicyclo[4.4.0]dec-5-ene (TBD) as an organocatalyst has also enabled the preparation of a library of terephthalic diamides (*13*), and a protic organocatalyst has also been reported for the synthesis of a small set of aminolyzed PET products (*14*). Several groups have used aminolysis of PET as a way to access sustainable feedstocks for polyurethane synthesis (*15*, *16*), as well as for epoxy resin and paint additives (*16*, *17*). Despite this focus on aminolysis, alcoholysis of PET may offer advantages, such as better cost effectiveness, more emphasis on green chemistry principles [as amines are often synthesized from alcohols (*18*, *19*)], and greater potential for biomass sourcing (*20*, *21*). Alcohols generally have lower toxicity (*22*, *23*), and there remains the potential to more readily recover upcycled PET-based polyester materials back into thermoplastic PET in an integrated terephthalic acid (TPA)–based circular economy (*24*). However, alcohols are less nucleophilic than amines, making PET alcoholysis more challenging. Even in the presence of catalysts, alcoholysis typically requires temperatures close to 200°C to obtain suitable reaction kinetics and conversions (*4*, *25*, *26*). In turn, this limits the potential pool of alcohols that can be applied on account of their low boiling points and/or low degradation temperatures. The glycolysis of PET to bis(2-hydroxyethyl)terephthalate (BHET) has proved to be viable at lower temperatures using organocatalysts (*27*) and leveraging the effect of a cosolvent (*28*, *29*). Other alcohols such as methanol (*30*, *31*), benzyl alcohol, 1-octanol, 1,3-propanediol, 1,6-hexanediol (*32*), and butanol (*33*) have been used with some success, albeit at high pressures (given that reaction temperatures commonly exceed 190°C).

**Fig. 1.**
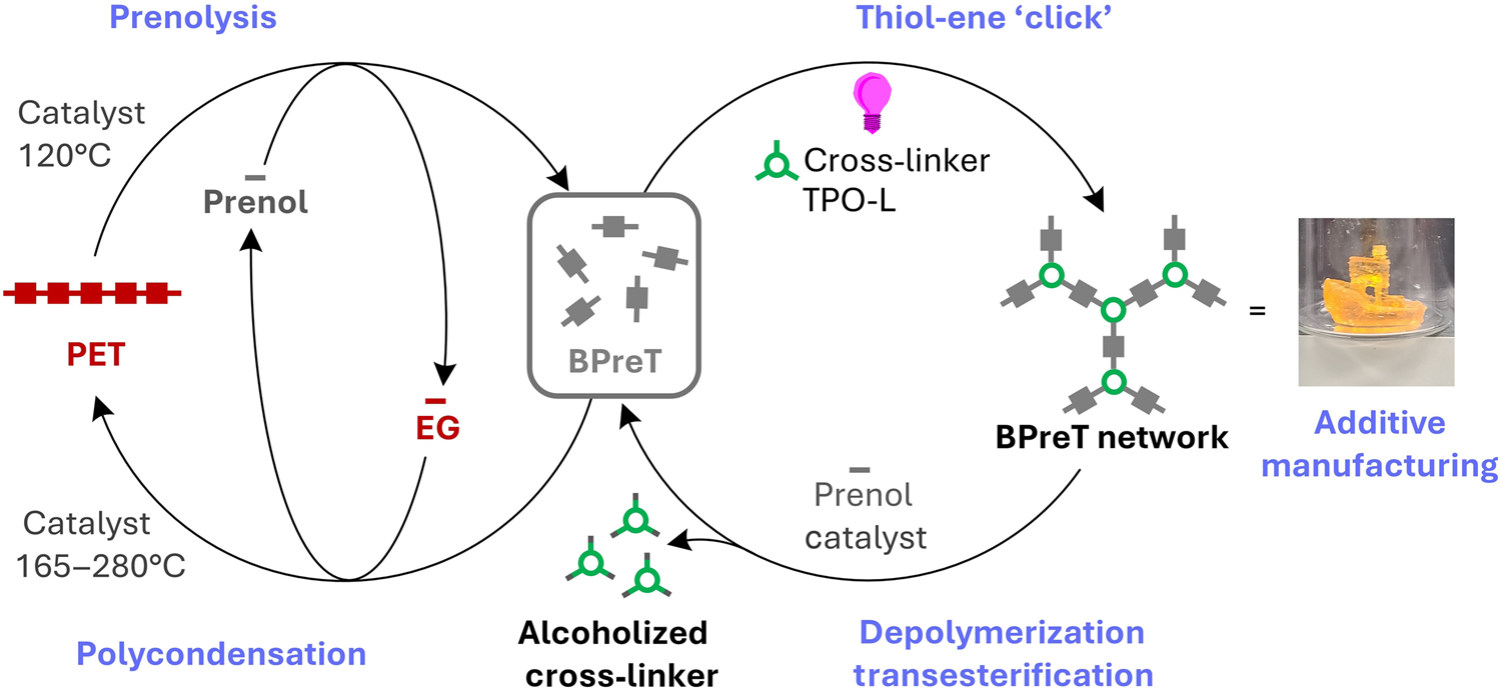
Integrated circular materials economy based upon the prenolysis of PET. Reaction of PET with prenol affords BPreT, which can either be used to synthesize PET (by reaction with EG) or PET-derived networks and photoresins for additive manufacturing. Treating BPreT networks with prenol reformed BPreT, closing the loop.

We envisaged an integrated TPA-based circular economy (Fig. 1) in which thermoplastic PET could be interconverted into thermoset 3D-printable materials and back by leveraging a range of bioderivable terpenoid alcohols with latent carbon-carbon double bonds to create materials with a wide range of properties. Critically, we hypothesized that the cross-linked materials created would be able to be reverted back to virgin-like PET, hence allowing interconversion between thermoplastic PET and photocured TPA and terpenoid alcohol–based materials. To achieve this, a protocol that enables PET depolymerization under milder reaction conditions, compared to those typically used in the CRM of PET, was required. We identified the zinc acetate (ZnOAc_2_)/4-dimethylaminopyridine (DMAP) dual-catalyst system (*34*) as an efficient means to promote PET alcoholysis at low temperature. The high activity of the dual-catalyst system enabled us to use bioderived alcohols, which would typically degrade at the elevated temperatures commonly used in PET chemical recycling (∼180°C). Hence, to advance PET recycling and upcycling, we initially explored low-boiling-point terpenoid alcohols as promising candidates. Owing to their bioderivable origin and inherent dual functionality, their applications can be expanded to create open-loop 3D-printable materials with properties that are controlled by judicious choice of the terpenoid alcohol used in the PET depolymerization.

## RESULTS

**Fig. 2.**
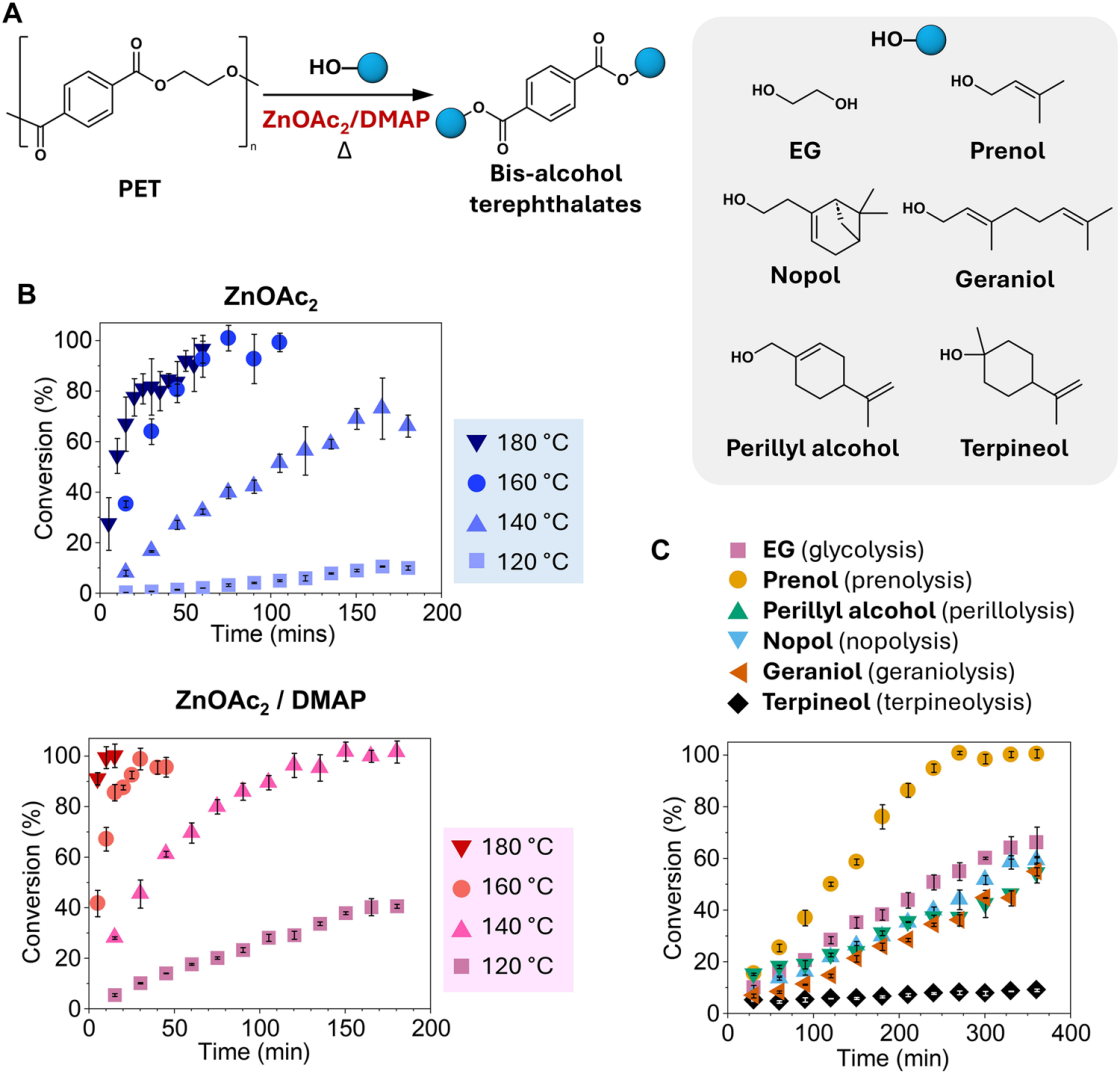
Kinetics of PET alcoholysis. (**A**) Scheme detailing the alcoholysis of PET and the terpenoids studied in this work. (**B**) Kinetic plots of ZnOAc_2_- and ZnOAc_2_/DMAP–mediated glycolysis at 120° to 180°C (10 mol % catalyst). (**C**) Kinetic plots of ZnOAc_2_/DMAP–catalyzed glycolysis compared to selected terpineolysis methods at 120°C (10 mol % catalyst).

Encouraged by the high performance of the ZnOAc_2_/DMAP dual-catalyst system in promoting PET glycolysis at 180°C (*34*), its catalytic activity was first evaluated and benchmarked against ZnOAc_2_ (in the absence of DMAP) across a range of temperatures from 120° to 180°C (Fig. 2, A and B). Depolymerization efficiencies were monitored by ^1^H nuclear magnetic resonance (NMR) spectroscopy, comparing the peak corresponding to the reference proton environment within both the BHET product and soluble PET oligomers, with mesitylene used as an internal standard [δ = 6.65 parts per million (ppm)]. The activity of ZnOAc_2_ decreased significantly as the temperature was lowered from 180° to 120°C, with minimal conversion observed at 120°C (11% conversion after 180 min; Fig. 2B). In contrast, ZnOAc_2_/DMAP showed greater activity at 120°C, reaching more than 40% conversion after 3 hours. To evaluate the role of DMAP in the dual-catalyst system with ZnOAc_2_, kinetic data were fitted to the pseudo-first-order kinetic model (*35*), diffusion-controlled shrinking core model, and kinetic-controlled shrinking core model (fig. S1) (*36*, *37*). The best fit was observed with the pseudo-first-order model (table S1), which was then used to calculate activation energies. Under these conditions, the activation energy for each system was found to be 134 ± 36 and 131 ± 7 kJ mol^−1^, respectively, in agreement with previous studies (fig. S2) (*37*, *38*). These findings also support our previous work that demonstrates the benefits of a dual-catalyst system, comprising both acidic and basic components, for PET depolymerization (*34*, *36*, *39*, *40*). Compared to state-of-the-art low-temperature catalysts from the literature, ZnOAc_2_/DMAP–catalyzed glycolysis performed favorably, reaching comparable or higher conversion than 1,8-diazabicyclo(5.4.0)undec-7-ene and choline chloride/ZnOAc_2_ (fig. S4) (*32*, *41*). Notably, the activity of ZnOAc_2_/DMAP at 120°C enabled us to explore alternative alcohols with lower boiling points and/or low thermal stability at ambient pressure.

To promote PET upcycling using biobased alcohols, we sought to explore the use of terpenoids: prenol, perillyl alcohol, nopol, geraniol, and terpineol (Fig. 2A). Preliminary depolymerization studies were conducted over 6 hours at 120°C using ZnOAc_2_/DMAP as the catalyst with conversion assessed by ^1^H NMR spectroscopy (Fig. 2B). While all alcohols yielded bis(terpene) terephthalates, reactivity with PET followed the following order: prenol > perillyl alcohol > geraniol > nopol > terpineol. Bis(prenol) terephthalate (BPreT) was the easiest product to isolate, followed by bis(perillyl alcohol) terephthalate (BPerT), which also precipitated readily (figs. S5 to S8). In contrast, bis(nopol) and bis(geraniol) terephthalates were harder to isolate, and the yield of bis(terpineol) terephthalate was too low for effective recovery (figs. S9 to S12). The volatility, odor activity, and oxidation sensitivity of some of these terpenoid alcohols did not present any challenges with respect to their application in these processes. Kinetic studies (Fig. 2C) comparing terpenoid-promoted PET depolymerization to glycolysis showed that prenol was the most effective, achieving complete PET conversion in only 4.5 hours, compared to glycolysis, which achieved only 66% in 6 hours. Other terpenoids (perillyl alcohol, geraniol, and nopol) displayed comparable depolymerization rates to ethylene glycol (EG), whereas, most likely as a result of the tertiary alcohol, terpineol yielded minimal conversion (9%). Kinetic modeling showed that the diffusion-controlled shrinking core model best fit the data with apparent rate constant (*k*_app_) values of 0.14 × 10^−2^, 0.34 × 10^−2^, 0.12 × 10^−2^, and 0.086 × 10^−2^ min^−1^ for glycolysis, prenolysis, perillyl alcoholysis, and nopolysis, respectively (table S2 and fig. S3).

We hypothesized that the high *k*_app_ for prenolysis was likely a consequence of the balance between the hydrophilicity and hydrophobicity of prenol, in comparison to other alcohols studied, which increased the wettability of PET and facilitated mass transfer. Swelling tests in EG, perillyl alcohol, geraniol, and nopol exhibited similar swelling effects at 120°C, whereas prenol presented a significant increase in swelling under the same conditions (fig. S13). Droplets of EG were found to have a contact angle of 46°, whereas that of prenol was not measurable on account of instantaneous wetting of the PET surface (fig. S14). In addition, dynamic mechanical thermal analysis (DMTA) thermograms of samples of PET swollen in prenol displayed a lower glass transition temperature (*T*_g_) than PET swollen in EG (fig. S15), further emphasizing that prenol swells PET more than EG. This enhanced swelling likely promotes a better mass transport of the alcohol and catalyst into the polymer during prenolysis, as has previously been observed with the addition of cosolvents (*28*, *29*). To examine the potential of prenol as a cosolvent to accelerate glycolysis, we investigated the effect of doping PET glycolysis reactions with prenol in loadings of 5 and 20 mol %. Rate enhancement of the pure glycolysis system was observed, attributable to the enhanced swelling, but depolymerization reached a lower conversion as a consequence of transesterification side reactions of the mixture of small-molecule terephthalates formed (figs. S16 to S24 and table S3). We also investigated the effect of reducing the loading of prenol in depolymerization. Prenolysis was viable at equivalents of terpenoid alcohol as low as 5 equiv, although a decreased rate of depolymerization and conversion to BPreT was observed (fig. S28). At 15 equiv of prenol, the rate was slightly enhanced, reaching full conversion after 3.5 hours, as a consequence of the higher effective reaction concentration, demonstrating the scope for the depolymerization efficiency to be optimized further by carefully balancing these parameters. The excess prenol used in the depolymerization reaction was found to be recoverable in high purity by simple distillation over at least two successive cycles (figs. S29 and S30) and could be reused in new prenolysis reactions with no loss of depolymerization efficiency (fig. S31).

To probe the commercial applicability of the optimized prenolysis system, we investigated its performance with PET waste. Postconsumer PET drink bottles could be depolymerized in high yield (>95%; fig. S27), albeit with a reduced depolymerization rate that is attributed to the use of PET flakes in contrast to the fine powder of the commercial PET model substrate. Last, EcoScale analysis, which provides a semiquantitative economical and ecological assessment of chemical reactions, showed that depolymerization using prenol achieved a score of 77 (table S4), underscoring its strong performance as a green and sustainable synthetic methodology (*42*, *43*). These results combined illustrate the attractiveness of this PET depolymerization system.

**Fig. 3.**
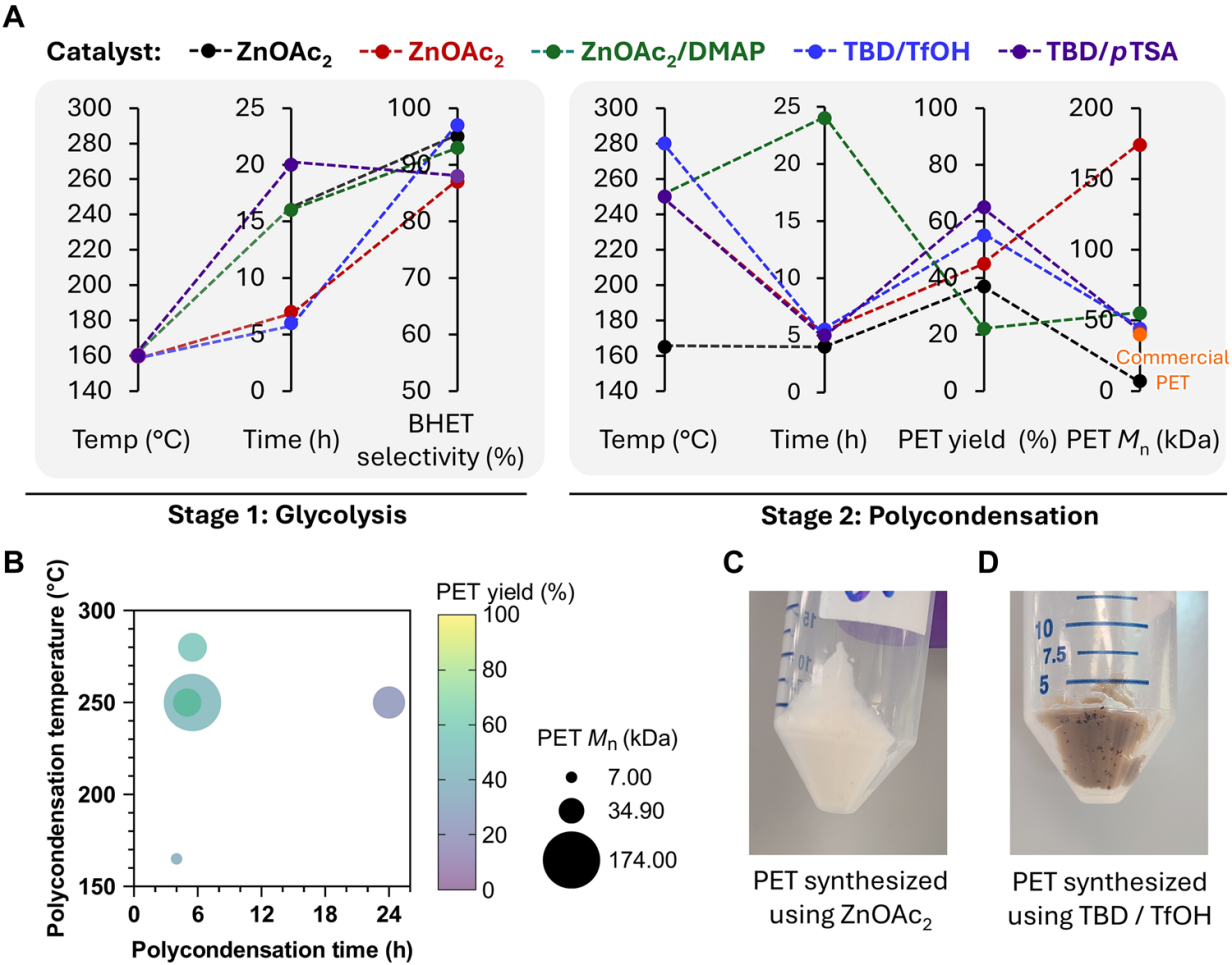
PET synthesis from BPreT. (**A**) Parallel coordinate plot detailing the two-stage, one-pot synthesis of PET from BPreT under varied conditions. Dashed lines are added to aid identification of related data points. h, hours. (**B**) Bubble plot describing the effect of polycondensation time and temperature on PET yield and Mn. (**C** and **D**) Pictures of PET synthesized in the presence of (C) ZnOAc_2_ and (D) TBD/TfOH.

To demonstrate that bis(terpenoid) terephthalate esters serve as a viable, complementary alternative to methanolysis or glycolysis in PET chemical recycling, we synthesized PET from BPreT via in situ conversion to BHET in a process analogous to that used industrially for the production of PET from dimethyl terephthalate (Fig. 3 and table S5). ZnOAc_2_-catalyzed glycolysis of BPreT proceeded readily at 165°C, producing BHET with 91% purity after 16 hours, as assessed by high-performance liquid chromatography (HPLC) (figs. S33 and S35). Placing the system under vacuum at 165°C was sufficient to drive off residual EG, furnishing PET as a white solid after 4 hours with a number-average molecular weight (*M*_n_) of 7 kDa [number-average degree of polymerization (DP_n_) = 74], as assessed by ^1^H NMR spectroscopy (fig. S36). To optimize the quality of the obtained PET, we explored the effects of catalyst [ZnOAc_2_, ZnOAc_2_/DMAP, TBD/triflic acid (TfOH), and TBD/*p*TSA (*para*-toluenesulfonic acid)], temperature (165° to 280°C), and reaction time (4 to 24 hours). The optimum conditions were found to yield off-white PET (*M*_n_ of 174 kDa; DP_n_ = 1814) through a two-step protocol consisting of a preliminary stirring of the reaction at 25°C for 48 hours, followed by heating to 250°C for 5.5 hours with ZnOAc_2_ (Fig. 3C and figs. S38 to S42) and a heavily discolored PET (*M*_n_ of 45 kDa; DP_n_ = 472) with TBD/TfOH (Fig. 3D). BPreT reacted readily with EG to form BHET under all conditions examined, with selectivity above 79% for BHET versus asymmetric terephthalate species with all catalysts and reaction times from 7 to 20 hours. This was higher than the commercial BHET, which had a purity of 74%, as assessed by HPLC. PET synthesized via the above-described process (*M*_n_ = 7 to 174 kDa) was comparable to commercial PET powder (*M*_n_ = 40 kDa; DP_n_ = 428; fig. S49). Our methodology demonstrates that high-purity BHET (79 to 94%) is readily produced from BPreT using a range of catalyst systems and that such conditions enable the one-pot synthesis of PET with a range of molecular weights. These findings support the potential of “prenolysis” as a viable strategy for enabling a circular PET economy under mild reaction conditions.

**Fig. 4.**
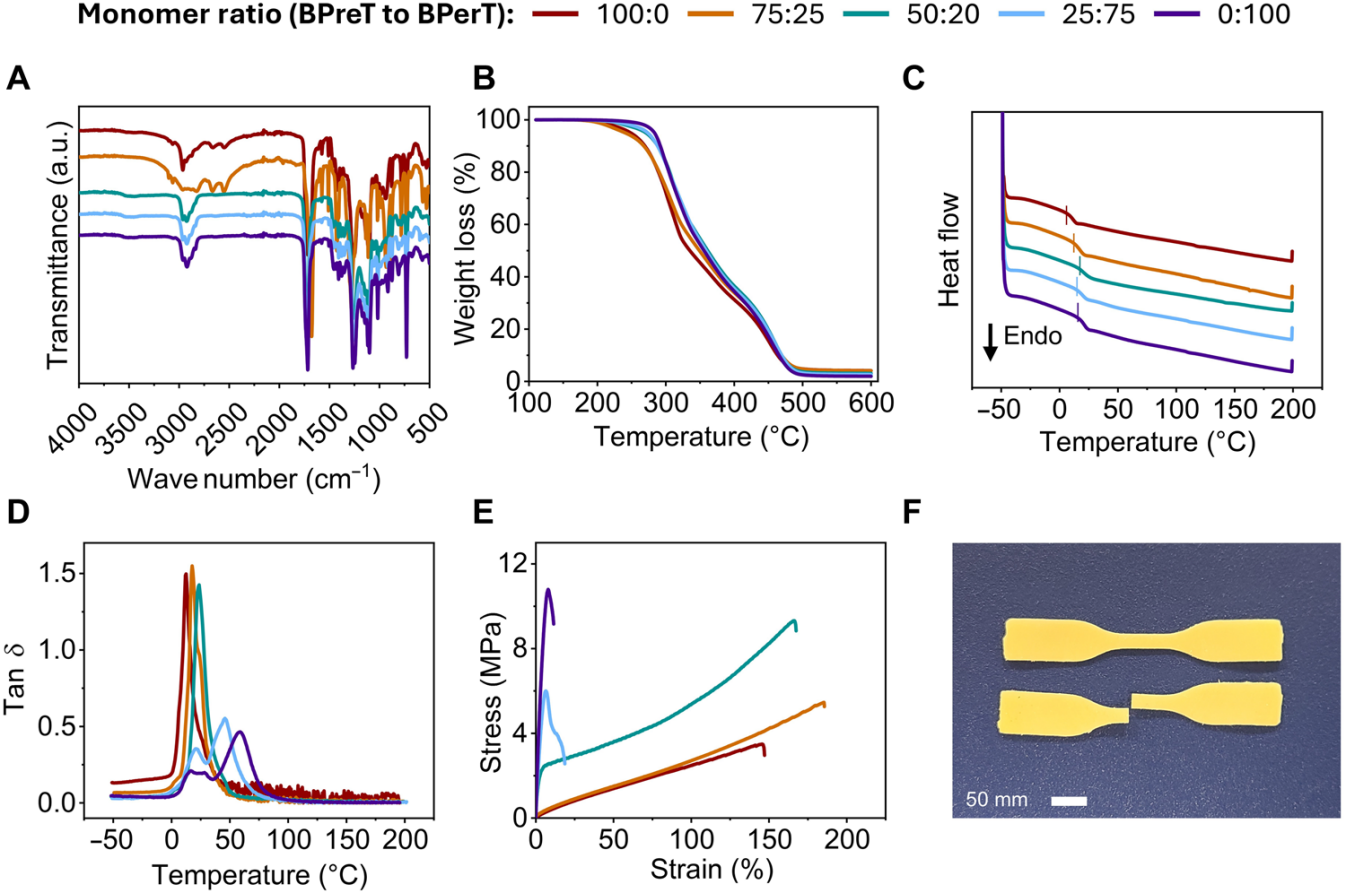
Thermomechanical properties of the BPreT/BPerT polymer networks. (**A**) FTIR spectra of the networks. a.u., arbitrary units. (**B**) TGA thermograms from 110° to 600°C at a heating rate of 10°C min^−1^. (**C**) DSC thermograms of the second heating cycle from −50° to 200°C at a heating rate of 10°C min^−1^, with the *T*_g_ indicated by the vertical lines. (**D**) DMA thermogram from −50° to 200°C at a heating rate of 5°C min^−1^, 1 Hz, and 1 μm. (**E**) Representative stress versus strain curves obtained from uniaxial tensile testing, tested at 10 mm min^−1^. (**F**) Example image of a 100% BPreT dogbone before and after tensile analysis.

The presence of unsaturated carbon-carbon double bonds in BPreT provided the potential to upcycle PET into cross-linked polymer networks. To this end, a photoinitiated radical thiol-ene reaction with trimethylolpropane tris(3-mercaptopropionate) (TMPMP) was performed with BPreT and BPerT, selected on the basis of their ready recovery from PET depolymerization. A series of networks with different thermal and mechanical properties were synthesized (Fig. 4 and table S6). Fourier transform infrared spectroscopy (FTIR) (Fig. 4A) showed the presence of thiol peaks at ν̄ = 2662 and 2552 cm^−1^ in networks containing 75% or more BPreT, suggesting incomplete conversion despite extended ultraviolet (UV) curing times. The reduced reactivity observed in BPreT networks was likely due to the increased steric hindrance of the trisubstituted alkene compared to the terminal alkene in BPerT. The thermal stability of the networks was initially investigated via thermogravimetric analysis (TGA), where the degradation temperature (*T*_d,5%_) was recorded as the temperature at 5% mass loss (Fig. 4B). The networks had similar *T*_d,5%_, ranging from 241° to 284°C, with networks containing a higher ratio of BPerT having a slight increase in thermal stability. Differential scanning calorimetry (DSC) demonstrated that all the networks were amorphous, displaying only a glass transition temperature (*T*_g_) ranging from 11.3° to 21.4°C with increased *T*_g_ with a higher BPerT content; no melting transition was observed (Fig. 4C). The observed increase in *T*_g_ is consistent with the molecular structure of the network, reflecting the reduced mobility of the cyclohexene ring in BPerT, which leads to a lower free volume in the network. This trend in *T*_g_ was further confirmed by DMTA (Fig. 4D and figs. S56 to S60). Notably, networks with 75 or 100% BPerT showed tan δ peak broadening and split into two peaks. This is attributed to visible β-relaxation peaks from the local movement of BPerT in the networks (*44*). In addition, the DMTA storage modulus (*E*′) plateaus at temperatures above the *T*_g_, suggesting that networks with a higher BPreT content are more tightly cross-linked than BPerT-rich networks (*45*), as expected from the shorter linkage in the monomer. Further investigation of the mechanical properties was performed via uniaxial tensile testing of networks, which demonstrated that the properties varied from elastomeric to brittle materials, as the proportion of BPerT increased (Fig. 4E and figs. S51 to S55). The polymer networks returned to their original size after break (Fig. 4F), displaying good elastic recovery attributed to covalent cross-links that preserved the network’s shape. Networks incorporating 100 or 75% BPreT showed elastomeric behavior [strain at break of 137 ± 13 and 162 ± 22%, respectively; ultimate tensile strength (UTS) of 3.3 ± 0.3 and 4.6 ± 0.7 MPa, respectively]. The network with 50% BPreT and 50% BPerT displayed ductile plastic behavior with a strain at break of 182 ± 31% and UTS of 8.8 ± 0.5 MPa. These tensile properties are not typically observed in cross-linked polymers and are characteristic of the material having high toughness (*46*). At increased proportions of BPerT (75 or 100%), the materials become brittle with strain at break values of 19.6 ± 5.3 and 21.5 ± 7.2%, respectively, and UTS values of 5.0 ± 0.8 and 9.0 ± 1.4 MPa, respectively. While a direct comparison to commercial materials is not possible because of differences in experimental setup, these materials nonetheless appear to have a lower tensile strength but higher elongation at break than previous reports of other recycled PET network materials, indicative of improved elasticity but reduced strength (*47*–*49*).

**Fig. 5.**
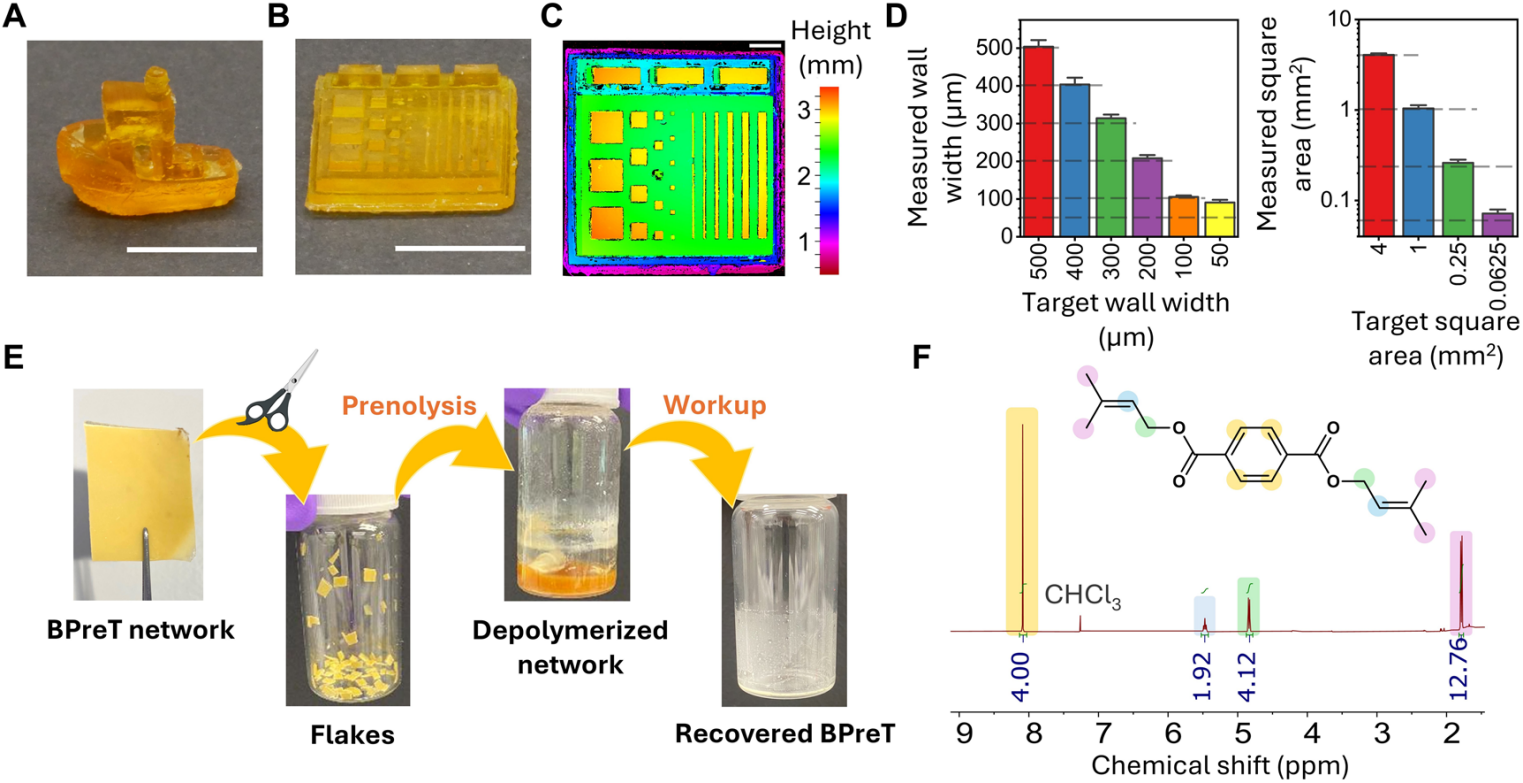
3D printing and depolymerization of upcycled materials. (**A**) Photographs of 3D-printed “3DBenchy” and (**B**) “bridges and walls” test square. Scale bar, 1 cm. (**C**) Optical microscopy image of 3D-printed “bridges and walls” test square. Scale bar, 2 mm. (**D**) Bar chart comparing the measured width of wall features and surface area of square features on 3D-printed test square to those of the model. (**E**) Depolymerization of a BPreT network to yield monomeric BPreT using ZnOAc_2_/DMAP as a catalyst at 180°C. (**F**) ^1^H NMR spectrum of the recovered BPreT (400 MHz, 298 K, CDCl_3_).

The rapid synthesis of robust photopolymer materials prompted us to explore whether these monomers could be used in a photoresin-based digital light processing (DLP) three-dimensional (3D) printing process. We chose the prenyl terephthalate monomer because of its improved solubility in TMPMP compared to the other synthesized terephthalate esters, thus simplifying the formulation of a homogeneous resin, facilitating a uniform photocuring process. By using *N*-methyl-2-pyrrolidone (NMP), a solvent with reported bioderivable potential (*50*, *51*), as a diluent, we formulated a resin comprising prenyl terephthalate, TMPMP, phenylbis(2,4,6-trimethylbenzoyl)phosphine oxide (BAPO) as the photoinitiator, and Sudan II as an opacifying agent (table S7). Rheological analysis revealed a viscosity of ∼100 mPa·s (fig. S61), which is suitable for DLP photocurable resins. Furthermore, photorheology of the resin indicated gelation in less than 6 s (fig. S62), which is appropriate for translation to DLP printing (*52*, *53*). The PET-derived photocurable resin was 3D printed using a commercial DLP printer equipped with a 405-nm light source, successfully producing a “3DBenchy” model known for its intricate overhanging geometries (Fig. 5A). To assess the printing resolution of the resin, a “bridges and walls” test structure was fabricated (Fig. 5B) and analyzed using optical microscopy (Fig. 5, C and D). Raised square features with surface areas as small as 0.25 mm^2^ were printed within an error of 5%, while more significant overcuring was noted for the smaller 0.0625-mm^2^ squares, with the error rising to 15%. Similarly, walls with widths as low as 100 μm were printed within a 6% error, increasing to 81% for the smallest 50-μm walls, demonstrating the ability of this material to construct 3D structures with challenging geometries that are difficult to achieve through conventional manufacturing methods.

Given the presence of terephthalate esters in the upcycled networks, we hypothesized that they could also undergo depolymerization to regenerate BPreT, enabling a closed-loop system for PET-derived upcycled network materials or recycling back to thermoplastic PET. To this end, a BPreT network was heated with ZnOAc_2_/DMAP dual-catalyst system (0.2 equiv to the esters in the network) in excess prenol for 24 hours. We attempted to isolate BPreT by recrystallization from a 1:1 (v/v) methanol/water mixture. However, the isolated yield was limited to only 13%, likely due to incomplete conversion resulting from the cross-linked nature of the material. To achieve the full depolymerization of the network, we increased the catalyst loading to 0.3 equiv and extended the reaction time to 110 hours. The network rapidly broke down into small fragments within 0.5 hours, resulting in an orange solution (Fig. 5E and fig. S63) that required extended reaction times to yield BPreT. BPreT was subsequently isolated from the crude reaction mixture using flash chromatography, achieving a 50% isolated yield (Fig. 5F). Repolymerization of the recovered BPreT yielded materials with thermomechanical properties consistent with the virgin networks (figs. S64 to 66), albeit with a small decrease in the *T*_g_ of the recycled polymer network, attributable to incorporation of fragments with a more flexible backbone resulting from incomplete depolymerization. These findings confirm that BPreT can be recovered from network materials, with the added value of the regenerated photoresin materials compensating for reduced depolymerization efficiency compared to PET, effectively closing the loop for upcycled PET through prenolysis and highlighting the versatility of BPreT as a key building block in a closed-loop materials system.

## DISCUSSION

This work presents low-temperature alcoholysis of PET as a practical strategy to promote a broader circular TPA-based materials economy. We propose that the use of active dual-catalyst systems, such as ZnOAc_2_/DMAP, could enable the use of bioderivable low-boiling-point alcohols for both PET recycling and upcycling. The possibility of using PET waste in the preparation of photocurable resins can open up further uses of plastic waste in additive manufacturing. The polymeric network developed in this work also proved to deliver diverse recyclable materials having a range of thermomechanical properties, starting with a limited pool of monomers. Although the research reported here focuses on the use of terpenoids, it paves the way to the use of other bioderivable and multifunctional alcohols, such as sugar-based alcohols, and demonstrates how a wide range of interconnected materials could be formulated from a simple and available resource.

## MATERIALS AND METHODS

### Materials

Anhydrous EG (>98.5%), DMAP (>99%), zinc acetate dihydrate [Zn(OAc)_2_·2H_2_O, >99.9%], (*S*)-(−)-perillyl alcohol (>95%), geraniol (98%), 3-methyl-2-buten-1-ol (prenol, 99%), (1*R*)-(−)-nopol (nopol; 98%), mesitylene (>99%), NMP (99.5%), TMPMP (>95.0%), BAPO (97%), and Sudan II (90%) were purchased from Sigma-Aldrich Company. PET was purchased from Goodfellow Company. TBD/TfOH (1:1) salt and TBD/*p*TSA (1:1) salt were synthesized following a previously reported procedure (*39*).

### Methods

#### NMR spectrometry

^1^H NMR kinetics studies were conducted on a Magritek Spinsolve 60-MHz HF Ultra. ^1^H and ^13^C NMR spectra were recorded on a Bruker Avance III 400 spectrometer equipped with a QNP (Quattro Nucleus Probe) operating at 400 MHz at 298 K. ^1^H spectra were referenced against solvent residual proton signals: δ = 7.26 ppm (CDCl_3_) and δ = 2.50 ppm [dimethyl sulfoxide-*d*_6_ (DMSO-*d*_6_)]; ^13^C spectra were referenced against solvent residual signals: δ = 77.16 ppm (CDCl_3_) and δ = 39.52 ppm (DMSO-*d*_6_). Spectra were processed using MestreNova (MestreLab, Spain). Kinetics studies used mesitylene as an internal standard (variable concentration, 7.2 to 6.2 ppm) and were quantified using the quantitation plug-in of MestreNova. Resonance multiplicities are described as s (singlet), d (doublet), t (triplet), m (multiplet), dd (doublet of doublets), dt (doublet of triplets), td (triplet of doublets), tt (triplet of triplets), tq (triplet of quartets), qd (quartet of doublets), or ddp (double doublet of pentets).

#### High-resolution mass spectrometry (HRMS)

Mass spectrometry was performed using a Waters Xevo G2-XS QTof with Atmospheric Solids Analysis Probe Positive (ASAP+) for direct solid sampling. Mass/charge ratio (*m*/*z*) scan completed from 0 to 1200 *m*/*z*.

#### Fourier transform infrared spectroscopy (FTIR)

FTIR spectroscopy was carried out using an Agilent Technologies Cary 630 FTIR spectrometer. Sixteen scans from 600 to 4000 cm^−1^ were taken at a resolution of 4 cm^−1^, and the spectra were corrected for background absorbance. Peaks above 1000 cm^−1^ are reported in wave number (cm^−1^) with the peak strength notated as w (weak), m (medium), or s (strong), as well as br (broad).

#### Flash column chromatography

Flash chromatography was performed on a Teledyne ISCO CombiFlash Rf+ Lumen equipped with an evaporative light scattering detector and a UV-visible (UV-Vis) photodiode array (PDA) detector operating at λ = 254 and 280 nm and used RediSep RF normal phase cartridges.

#### Thermogravimetric analysis (TGA)

TGA thermograms were obtained using a TGA/DSC 1-Thermogravimetric Analyzer (Mettler Toledo). Thermograms were recorded under a N_2_ (nitrogen gas) atmosphere at a heating rate of 10°C min^−1^, from 110° to 600°C, with an average sample weight of ∼10 mg. Aluminum pans were used for all samples. Decomposition temperatures were reported as the 5% weight loss temperature (*T*_d,5%_)_._

#### Differential scanning calorimetry (DSC)

The thermal characteristics of the polymers were determined using DSC (STARe system DSC3, Mettler Toledo) from −50° to 200°C at a heating rate of 10°C min^−1^ for two heating/cooling cycles, unless otherwise specified. The glass transition temperature (*T*_g_) was determined from the inflection point in the second heating cycles of DSC.

#### UV-Vis spectroscopy

The UV-Vis spectra of BHET and BPreT were acquired using a Cary 3500 Multicell UV-Vis Spectrophotometer and quartz glass cuvettes. The analytes were dissolved in acetonitrile, and spectra over a wavelength range of 200 to 800 nm were gathered.

#### High-performance liquid chromatography (HPLC)

HPLC was performed on a Shimadzu Prominence system composed of a DGU-20A 5R degassing unit, LC-20AD pump, SIL-20AC HT Autosampler, CBM-20A system controller, CTO-20AC column oven, SPD-M20A PDA Detector, RF-20A Fluorescence Detector, and FRC-10A fraction collector. The analytical HPLC method used a Varian Polaris 3u C18-A 250 × 4.6-mm reverse phase column (180-Å pore size and 5-μm particle size) with the column oven set to 60°C. The mobile phases were water and formic acid (0.1%, v/v) for the aqueous phase and acetonitrile and formic acid (0.1%, v/v) for the organic phase. The flow rate was 0.6 ml/min. The gradient increased from 5% organic phase initially to 95% organic phase after 25 min before returning to 5% organic phase and holding for the following 5 min for a total runtime of 30 min per sample. The PDA detector was set to monitor 200 to 400 nm at a cell temperature of 40°C, and the fluorescence detector was set to a λ_ex_ of 320 nm and a λ_em_ of 385 nm. Samples were dissolved in acetonitrile (final concentration <5 mM based on 100% conversion) and filtered through syringe filters (0.2-μm polytetrafluoroethylene) before injection. The injection volume was 10 μl per sample. The resulting data were processed, the chromatogram at 285 nm was extracted, and the peak area for each peak was quantified using a custom Python script (see the Supplementary Materials) that uses the hplc-py package (*54*). Expressing each peak area as a fraction of the total peak area for the sample gave the relative fraction of each species and, therefore, the % purity on the basis of the terephthalate chromophore. Peak retention times were assigned using pure reference samples of TPA, BHET, dimer, trimer, and commercial BHET [containing mono-(2-hydroxyethyl) terephthalic acid, BHET, dimer, and trimer], as previously disclosed (*36*).

#### Uniaxial tensile testing

Dumbbell-shaped samples were cut directly from the synthesized films using a custom ASTM Die D-638 Type V. Tensile tests at a stretching speed of 5 mm min^−1^ were carried out using a Testometric M350-5CT universal mechanical testing instrument fitted with a load cell of 5 kN at room temperature (21 ± 1°C). The gauge length was set as 6.3 mm, and the crosshead speed was set to 5 mm min^−1^. The dimensions of the neck of the specimens were 6.3 mm in length, 1.6 mm in width, and 0.4 to 0.7 mm in thickness. The reported results are average values from three individual measurements.

#### Dynamic mechanical analysis (DMA)

DMTA data were obtained using a Mettler Toledo DMA (dynamic mechanical analysis) 1 star system and analyzed using the software package STARe V13.00a (build 6917). Thermal sweeps were conducted using films (*L* × *W* × thickness = 9.00 mm × 6.00 mm × 0.5 to 0.7 mm) cooled to −50°C and held isothermally for ∼5 min. Storage and loss moduli, as well as the loss factor (ratio of *E*″ and *E*′; tan δ), were probed as the temperature was swept from −50° to 150°C at 5°C min^−1^ and 1 Hz.

#### 3D printing

DLP 3D printing was performed on an unmodified MiiCraft Ultra 125 printer with a 405-nm light source. Layers were 50 μm thick and cured for 15 s, and two base layers were used and cured for 60 s each.

#### Contact angle measurements

Drop shape analysis was performed using a Krüss DSA25E Optical Tensometer. Thirty microliters of the analyzed liquid was manually added to a commercial PET film placed on the stage. Measurements were made using Krüss Advance software.

#### General procedure for the glycolysis of PET

Glycolysis reactions were conducted using a modified version of our previously reported procedure (*34*). A 20-ml scintillation vial was charged with EG (2.8 ml, 50 mmol, 20 equiv), Zn(OAc)_2_·2H_2_O (55 mg, 0.25 mmol, 0.10 equiv), and DMAP (31 mg, 0.25 mmol, 0.10 equiv) and sealed with a screw cap. The mixture was brought to the desired temperature (120° to 180°C) in a preheated metal heating block with stirring at 1000 rpm. PET (480 mg, 2.5 mmol, 1 equiv) was added to the solution, and 0.1-ml aliquots were taken at regular time points. Mesitylene was used as an internal standard and added to DMSO-*d*_6_ (15 ml) such that the number of moles of mesitylene in 0.4 ml of the NMR solution was the same as the number of moles of product in the aliquot at a theoretical full conversion. Reaction aliquots were diluted in 0.4 ml of NMR solution and filtered through a syringe filter before ^1^H NMR spectroscopy was conducted on a benchtop NMR spectrometer (60 MHz). The terephthalate aromatic peak (δ = 8.1 ppm) was integrated with respect to the mesitylene aromatic peak (δ = 6.7 ppm) to obtain conversion at a specific time point.

*Bis(2-hydroxyethyl) terephthalate (BHET).* ^1^H NMR (400 MHz, DMSO-*d*_6_, 298 K) δ = 8.12 (s, 4H; Ar-H), 4.96 (t, *J* = 5.7 Hz, 2H; OH), 4.34–4.28 (m, 4H; CH_2_), 3.75–3.68 (m, 4H; CH_2_). ^13^C NMR (100 MHz, DMSO-*d*_6_, 298 K) δ = 165.2 (C**C**(O)O), 133.8 (C═**C**(C)C), 129.5 (C═**C**C), 67.0 (O**C**C), 59.0 (C**C**OH).

#### General procedure for the alcoholysis of PET

A 20-ml scintillation vial was charged with an alcohol (50 mmol, 20 equiv), Zn(OAc)_2_·2H_2_O (55 mg, 0.25 mmol, 0.10 equiv), and DMAP (31 mg, 0.25 mmol, 0.10 equiv) and sealed with a screw cap. The mixture was brought to 120°C in a preheated metal heating block with stirring at 1000 rpm. PET (480 mg, 2.5 mmol, 1 equiv) was added to the solution, and 0.1-ml aliquots were taken at regular time points. Mesitylene was used as the internal standard and added to DMSO-*d*_6_ (15 ml) such that the number of moles of mesitylene in 0.4 ml of the NMR solution was the same as the number of moles of product in the aliquot at a theoretical full conversion. Reaction aliquots were diluted in 0.4 ml of NMR solution and filtered through a syringe filter before ^1^H NMR spectroscopy was conducted on a benchtop NMR spectrometer (60 MHz). The terephthalate aromatic peak (δ = 8.1 ppm) was integrated with respect to the mesitylene aromatic peak (δ = 6.7 ppm) to obtain conversion at a specific time point. The depolymerized product was isolated by filtering the reaction mixture while it was hot. The product was precipitated from the supernatant by the addition of methanol until all alcohol dissolved, before water was added to a point just before phase separation. This mixture was aged in a fridge at 4°C overnight and filtered to isolate the crude solid, which was further purified by either flash chromatography (1:1 ethyl acetate:hexane) or dissolution in the minimum amount of boiling acetone, followed by dropwise addition of water.

*Bis(prenyl)terephthalate (BPreT).* Yield: 0.506 g, 67%, colorless crystalline solid. ^1^H NMR (400 MHz, chloroform-*d*, 298 K) δ = 8.11 (s, 4H; Ar-H), 5.50 (ddp, *J* = 8.6, 5.7, 1.4 Hz, 2H; OCH_2_C**H═**C), 4.86 (dt, *J* = 7.3, 0.9 Hz, 4H; OC**H**_**2**_CH), 1.81 (dd, *J* = 7.2, 1.3 Hz, 12H; CH═CC**H**_**3**_), 1.57 (s; H_2_O). ^13^C NMR (100 MHz, chloroform-*d*, 298 K) δ = 165.9 (C**C**═O(O)), 139.6 (Ar-**C**HCOO), 134.2 (CH_2_**C**H═C), 129.5 (Ar-**C**H), 118.4 (CH═**C**CH_3_(CH_3_)), 62.3 (C═O(O)**C**H_2_CH), 25.8 (CH═C**C**H_3_(CH_3_)), 18.1 (CH═CCH_3_(**C**H_3_)). FTIR wavenumber/cm^−1^: 2903 (w, br, C─H), 1715 (s, C═O), 1501 (w, C═C). HRMS (ASAP): *m*/*z* calcd. for C_18_H_22_O_4_ + H^+^: 303.1596 [M + H]^+^; found: 303.1600. Elemental analysis: calcd. for C_18_H_22_O_4_: C, 71.50; H, 7.33; N, 0; found: C, 71.97; H, 7.15; N, 0.

*Bis(perillyl)terephthalate (BPerT).* Yield: 0.654 g, 60%, colorless crystalline solid. ^1^H NMR (400 MHz, chloroform-*d*, 298 K) δ = 8.14 (s, 3H; Ar-H), 5.88 (s, 2H; C═C**H**CH_2_), 4.76 (dd, *J* = 4.8, 1.4 Hz, 8H; OC**H**_**2**_C; C═C**H**_**2**_(CH_3_)), 2.21 (s, 6H; C═CHC**H**_**2**_CH; CH_2_(CH_2_)C**H**C═CH_2_(CH_3_)), 2.05 (d, *J* = 14.7 Hz, 4H; CH═C(CH_2_)C**H**_**2**_CH_2_), 1.95–1.87 (m, 4H; CH_2_C**H**_**2**_CH), 1.77 (dd, *J* = 1.5, 0.8 Hz, 6H; C═CH_2_(C**H**_**3**_)). ^13^C NMR (100 MHz, chloroform-*d*, 298 K) δ = 165.7 (C**C═**O(O)), 149.5 (C═**C**HCH_2_), 134.2 (OCH_2_**C**═CH(CH_2_)), 132.4 (**Ar**C═O(O)), 129.6 (Ar), 126.2 (CH**C**═CH_2_(CH_3_)), 108.9 (C═**C**H_2_(CH_3_)), 69.3 (C═O(O)**C**H_2_C), 40.8 ((CH_2_)CH_2_**C**HC), 30.5 (CH**C**H_2_CH), 27.3 (CH_2_**C**H_2_CH), 26.5 (C**C**H_2_CH_2_), 20.8 (C=CH_2_(**C**H_3_)). FTIR wave number/cm^−1^: 3033 (w, br, C─H), 1796 (s, C═O), 1625 (w, C═C). HRMS (ES+): *m*/*z* calcd. for C_28_H_34_O_4_ + Na^+^: 457.2368 [M + Na]^+^; found: 457.2365. Elemental analysis: calcd. for C_28_H_34_O_4_: C, 77.39; H, 7.89; N, 0; found: C, 77.31; H, 7.54; N, 0.

*Bis(nopyl)terephthalate (BNopT).* Yield: 0.564 g, 49%, colorless crystalline solid. ^1^H NMR (400 MHz, chloroform-*d*, 298 K) δ = 8.10 (s, 4H; Ar-H), 5.39 (tt, *J* = 3.0, 1.4 Hz, 2H; C═C**H**CH_2_), 4.38 (td, *J* = 6.9, 2.8 Hz, 4H; C=O(O)C**H**_**2**_CH_2_), 2.52–2.36 (m, 8H; CH_2_C**H**_**2**_C, CHC**H**_**2**_CH), 2.35–2.18 (m, 2H; CH_2_C**H**C(CH_2_)), 2.14 (qd, *J* = 6.1, 2.6 Hz, 4H; CHC**H**_**2**_CH), 1.30 (s, 6H; CC**H**_**3**_(CH_3_)), 1.19 (d, *J* = 8.5 Hz, 2H; CC**H**C(CH_2_)), 0.85 (s, 6H; CCH_3_(C**H**_**3**_)). ^13^C NMR (100 MHz, chloroform-*d*, 298 K) δ = 165.8 (C**C**═O(O)), 144.1 (CH_2_**C**═CH(CH_2_)), 134.2 (**Ar**C═O(O)), 129.5 (Ar), 119.1 (C═**C**HCH_2_), 63.7 (C═O(O)**C**H_2_CH_2_), 45.8 (C**C**HC(CH_2_)), 40.7 (CH_2_**C**HC(CH_2_)), 38.0 ((CH)CH**C**CH_3_(CH_3_)), 36.0 (CH_2_**C**H_2_C), 31.7 (CH**C**H_2_CH), 31.4 (CH**C**H_2_CH), 26.3 (C**C**H_3_(CH_3_)), 21.2 (CCH_3_(**C**H_3_)). FTIR wave number/cm^−1^: 3077 (m, br, C─H), 1801 (s, C═O), 1526 (w, C═C). HRMS (ES+): *m*/*z* calcd. for C_30_H_38_O_4_ + Na^+^: 463.2848 [M + Na]^+^; found: 463.2851. Elemental analysis: calcd. for C_30_H_38_O_4_: C, 77.89; H, 8.28; N, 0; found: C, 78.0; H, 8.38; N, 0.

*Bis(geraniol)terephthalate (BGerT).* Yield: 0.636 g, 58%, yellow oil. ^1^H NMR (400 MHz, chloroform-*d*, 298 K) δ = 8.09 (s, 4H; Ar-H), 5.50 (tq, *J* = 7.1, 1.3 Hz, 2H; CH_2_C**H**═C), 5.14–5.04 (m, 2H, CH_2_C**H**═C), 4.89 (d, *J* = 7.1 Hz, 4H, C═O(O)C**H**_**2**_CH), 2.22–1.96 (m, 8H, CC**H**_**2**_CH_2_, CH_2_C**H**_**2**_CH), 1.80 (s, 6H, CC**H**_**3**_), 1.70 (s, 6H, CC**H**_**3**_(CH_3_)), 1.63 (s, 6H, CCH_3_(C**H**_**3**_)). ^13^C NMR (100 MHz, chloroform-*d*, 298 K) δ = 166.02 (Ar**C**═O(O)), 142.92 (**Ar**C═O(O)), 134.34 (OCH_2_**C**H═C), 132.03 (CH_2_**C**H═C), 129.64 (Ar), 123.82 (CH═**C**CH_2_(CH_3_)), 118.24 (CH═**C**CH_3_(CH_3_)), 62.42 (C═O(O)**C**H_2_CH), 39.69 (C**C**H_2_CH_2_), 26.41 (CH_2_**C**H_2_CH), 25.81 (CH═CCH_2_(**C**H_3_)), 17.82 (CH═C**C**H_3_(CH_3_)), 16.71 (CH═CCH_3_(**C**H_3_)). FTIR wave number/cm^−1^: 2960 (m, C─H), 2910 (m, C─H), 2848 (m, C─H), 1714 (s, C═O), 1667 (w, C═C), 1575 (w, C═C), 1500 (w, C═C). HRMS (ASAP): *m*/*z* calcd. for C_28_H_38_O_4_ + H^+^: 439.2848; found: 439.2850. Elemental analysis: calcd. for C_18_H_22_O_4_: C, 76.68; H, 8.73; N, 0; found: C, 75.84; H, 7.83; N, 0.

#### Exemplar procedure for the recovery of prenol from depolymerization of PET

PET powder (11.00 g, 0.057 mmol, 1 equiv), Zn(OAc)_2_·2H_2_O (1.14 g, 0.0057 mmol, 0.1 equiv), DMAP (0.70 g, 0.0057 mmol, 0.1 equiv), and prenol (110 ml, 1.14 mmol, 20 equiv) were combined in a round-bottom flask and stirred at 120°C for 17 hours. The reaction was then allowed to cool to room temperature before excess prenol was isolated by distillation (140°C) in yields of 80% from the first cycle and 67% from the second cycle and then reused in subsequent depolymerization reactions.

#### General procedure for the synthesis of PET from bis(prenyl)terephthalate

To a solution of BPreT (100 mg, 0.33 mmol, 1 equiv) in EG (1 ml, 18.19 mmol, 55 equiv), an appropriate catalyst (0.017 mmol, 0.05 equiv) was added, and the reaction mixture was heated to 165°C under nitrogen. After an appropriate amount of time (5.5 to 20 hours), an aliquot of the reaction mixture was taken (one drop) and diluted in DMSO-*d*_6_ before conversion and selectivity were assessed by ^1^H NMR spectroscopy on a benchtop NMR spectrometer (60 MHz) and by HPLC, respectively. The reaction mixture was placed under vacuum and heated to the desired temperature (165° to 280°C). It was noted that residual EG boiled off in a few seconds after vacuum was applied. After the reaction proceeded for the desired amount of time (4 to 24 hours) and until no further visual changes were observed, the reaction mixture was flushed with nitrogen and cooled to room temperature. To the crude reaction mixture, a chloroform/trifluoroacetic acid mixture (5 ml; 8:1, v/v) was added and left to stir for 5 min until no further dissolution of solids occurred (longer time may be needed for high molecular weights). The undissolved solids were filtered off, and the supernatant was precipitated into methanol (50 ml). The solid product was collected by centrifugation and dried to yield PET as a white to brown solid. To assess PET *M*_w_ (weight-average molecular weight), the product was dissolved in chloroform-*d*/trifluoroacetic acid (0.7 ml; 8:1, v/v), and the molecular weight was determined via ^1^H NMR spectroscopy by comparing the terminal glycol peak (δ = 4.17 ppm) to the backbone glycol peak (δ = 4.66 to 5.10 ppm) (*55*). ^1^H NMR (400 MHz, 298 K, chloroform-*d*/trifluoroacetic acid; 8:1, v/v) δ = 8.11 (s, 4H, Ar-H), 5.10–4.66 (m, 4H, CH_2_), 4.65–4.56 (m, 2H, ether bridge CH_2_), 4.19–4.14 (m, 2H, CH_2_), 4.13–4.05 (m, 2H, ether bridge CH_2_).

#### Synthesis of cross-linked networks

A 7-ml vial was charged with BPreT and BPerT at a ratio according to table S5 (totaling 1.0 mmol, 3.0 equiv), TMPMP (220 μl, 0.67 mmol, 2.0 equiv), Igracure TPO-L (19 mg, 3 wt %), and tetrahydrofuran (2 ml). The mixture was briefly mixed to homogenize, then transferred to a Teflon beaker, and irradiated under UV light (*h*ν = 365 nm) for 1.5 to 2.5 hours). The resultant films were left to dry for 24 hours at ambient temperature and then postcured in a vacuum oven at 120°C for a further 24 hours.

#### Formulation of resin for 3D printing

BPreT (2.810 g, 9.29 mmol, 3.0 equiv), TMPMP (2.469 g, 6.20 mmol, 2.0 equiv), Sudan II (1.9 mg, 6.87 μg, 0.002 equiv), and NMP (2.0 ml) were combined in a 20-ml scintillation vial and stirred at room temperature overnight to form a homogeneous resin. BAPO (0.156 g, 0.373 mmol, 0.12 equiv) was then added, and the resin was stirred for a further hour. Resin formulation is summarized in table S6.

#### General procedure for the depolymerization of cross-linked networks

A 20-ml scintillation vial was charged with a BPreT network (99 mg, 0.73 mmol in relation to esters, 1 equiv), prenol (1.5 ml, 15 mmol, 20 equiv), Zn(OAc)_2_·2H_2_O (49 mg, 0.22 mmol, 0.3 equiv), and DMAP (26 mg, 0.22 mmol, 0.3 equiv) and sealed. The mixture was brought to 120°C and stirred for 110 hours. An aqueous 5% HCl (hydrochloric acid) solution (10 ml) was added to the mixture and extracted with dichloromethane (3 × 10 ml). The combined organic layer was then washed with water (2 × 10 ml) and brine (10 ml) and dried over anhydrous MgSO_4_ (magnesium sulfate). After the removal of the salt and subsequent concentration, the residue was purified via flash chromatography (*n*-hexane/ethyl acetate, 10:0 to 9:1) to yield BPreT as a colorless solid (31 mg, 50%).

## Supplementary Materials

### This PDF file includes:

Figs. S1 to S66 Tables S1 to S5 Supplementary Text References

## References

[1] R. Geyer, J. R. Jambeck, K. L. Law, Production, use, and fate of all plastics ever made. Sci. Adv. 3, e1700782 (2017). 28776036 10.1126/sciadv.1700782 PMC5517107

[2] M. Babaei, M. Jalilian, K. Shahbaz, Chemical recycling of polyethylene terephthalate: A mini-review. J. Environ. Chem. Eng. 12, 112507 (2024).

[3] E. Barnard, J. J. Rubio Arias, W. Thielemans, Chemolytic depolymerisation of PET: A review. Green Chem. 23, 3765–3789 (2021).

[4] V. Sinha, M. R. Patel, J. V. Patel, PET waste management by chemical recycling: A review. J. Polym. Environ. 18, 8–25 (2010).

[5] L. T. Korley, T. H. Epps III, B. A. Helms, A. J. Ryan, Toward polymer upcycling—Adding value and tackling circularity. Science 373, 66–69 (2021). 34210879 10.1126/science.abg4503

[6] S. C. Kosloski-Oh, Z. A. Wood, Y. Manjarrez, J. P. de Los Rios, M. E. Fieser, Catalytic methods for chemical recycling or upcycling of commercial polymers. Mater. Horiz. 8, 1084–1129 (2021). 34821907 10.1039/d0mh01286f

[7] S. E. Lewis, B. E. Wilhelmy, F. A. Leibfarth, Organocatalytic C–H fluoroalkylation of commodity polymers. Polym. Chem. 11, 4914–4919 (2020).

[8] C. Jehanno, J. W. Alty, M. Roosen, S. De Meester, A. P. Dove, E. Y.-X. Chen, F. A. Leibfarth, H. Sardon, Critical advances and future opportunities in upcycling commodity polymers. Nature 603, 803–814 (2022). 35354997 10.1038/s41586-021-04350-0

[9] J. C. Worch, A. P. Dove, 100th anniversary of macromolecular science viewpoint: Toward catalytic chemical recycling of waste (and future) plastics. ACS Macro Lett. 9, 1494–1506 (2020). 35617072 10.1021/acsmacrolett.0c00582

[10] G. W. Coates, Y. D. Getzler, Chemical recycling to monomer for an ideal, circular polymer economy. Nat. Rev. Mater. 5, 501–516 (2020).

[11] J. P. Tan, J. Tan, N. Park, K. Xu, E. D. Chan, C. Yang, V. A. Piunova, Z. Ji, A. Lim, J. Shao, Upcycling poly (ethylene terephthalate) refuse to advanced therapeutics for the treatment of nosocomial and mycobacterial infections. Macromolecules 52, 7878–7885 (2019).

[12] M. Poderyte, R. Lima, P. I. Golbækdal, D. W. Juhl, K. L. Olesen, N. C. Nielsen, A. Lanza, J.-W. Lee, Repurposing polyethylene terephthalate plastic waste to capture carbon dioxide. Sci. Adv. 11, eadv5906 (2025). 40911693 10.1126/sciadv.adv5906 PMC12412650

[13] K. Fukushima, J. M. Lecuyer, D. S. Wei, H. W. Horn, G. O. Jones, H. A. Al-Megren, A. M. Alabdulrahman, F. D. Alsewailem, M. A. McNeil, J. E. Rice, Advanced chemical recycling of poly (ethylene terephthalate) through organocatalytic aminolysis. Polym. Chem. 4, 1610–1616 (2013).

[14] J. Demarteau, I. Olazabal, C. Jehanno, H. Sardon, Aminolytic upcycling of poly (ethylene terephthalate) wastes using a thermally-stable organocatalyst. Polym. Chem. 11, 4875–4882 (2020).

[15] G. Mir Mohamad Sadeghi, R. Shamsi, M. Sayaf, From aminolysis product of PET waste to novel biodegradable polyurethanes. J. Polym. Environ. 19, 522–534 (2011).

[16] T. Spychaj, E. Fabrycy, S. Spychaj, M. Kacperski, Aminolysis and aminoglycolysis of waste poly (ethylene terephthalate). J. Mater. Cycles Waste Manag. 3, 24–31 (2001).

[17] E. Bulak, I. Acar, The use of aminolysis, aminoglycolysis, and simultaneous aminolysis–hydrolysis products of waste PET for production of paint binder. Polym. Eng. Sci. 54, 2272–2281 (2014).

[18] J. He, J. W. Kim, K. Yamaguchi, N. Mizuno, Efficient catalytic synthesis of tertiary and secondary amines from alcohols and urea. ChemInform 41, 9888–9891 (2010). 10.1002/anie.200905385 19943307

[19] C. Gunanathan, D. Milstein, Selective synthesis of primary amines directly from alcohols and ammonia. Angew. Chem. Int. Ed. 47, 8661–8664 (2008). 10.1002/anie.200803229 18846519

[20] M. Shiramizu, F. D. Toste, Deoxygenation of biomass-derived feedstocks: Oxorhenium-catalyzed deoxydehydration of sugars and sugar alcohols. Angew. Chem. Int. Ed. 51, 8082–8086 (2012). 10.1002/anie.201203877 22764085

[21] D. Sun, S. Sato, W. Ueda, A. Primo, H. Garcia, A. Corma, Production of C4 and C5 alcohols from biomass-derived materials. Green Chem. 18, 2579–2597 (2016).

[22] P. Roose, K. Eller, E. Henkes, R. Rossbacher, H. Höke, “Aliphatic amines” in *Ullmann’s Encyclopedia of Industrial Chemistry*, B. Elvers, S. Hawkins, G. Schulz, Eds. (VCH, Weinheim, ed. 5, 1985), vol. A2, pp. 1–55.

[23] O. Strubelt, M. Deters, R. Pentz, C. Siegers, M. Younes, The toxic and metabolic effects of 23 aliphatic alcohols in the isolated perfused rat liver. Toxicol. Sci. 49, 133–142 (1999). 10367351 10.1093/toxsci/49.1.133

[24] C. C. Westover, T. E. Long, Envisioning a BHET economy: Adding value to PET waste. Sustain. Chem. 4, 363–393 (2023).

[25] R. López-Fonseca, I. Duque-Ingunza, B. De Rivas, S. Arnaiz, J. I. Gutierrez-Ortiz, Chemical recycling of post-consumer PET wastes by glycolysis in the presence of metal salts. Polym. Degrad. Stab. 95, 1022–1028 (2010).

[26] S. Baliga, W. T. Wong, Depolymerization of poly (ethylene terephthalate) recycled from post-consumer soft-drink bottles. J. Polym. Sci. A 27, 2071–2082 (1989).

[27] I. Olazabal, E. J. Luna Barrios, S. De Meester, C. Jehanno, H. Sardon, Overcoming the limitations of organocatalyzed glycolysis of poly (ethylene terephthalate) to facilitate the recycling of complex waste under mild conditions. ACS Appl. Polym. Mater. 6, 4226–4232 (2024). 38633816 10.1021/acsapm.4c00326 PMC11019730

[28] N. H. Le, T. T. Ngoc Van, B. Shong, J. Cho, Low-temperature glycolysis of polyethylene terephthalate. ACS Sustain. Chem. Eng. 10, 17261–17273 (2022).

[29] E. Luna, I. Olazabal, M. Roosen, A. Müller, C. Jehanno, M. Ximenis, S. De Meester, H. Sardon, Towards a better understanding of the cosolvent effect on the low-temperature glycolysis of polyethylene terephthalate (PET). Chem. Eng. J. 482, 148861 (2024).

[30] T. Sako, T. Sugeta, K. Otake, N. Nakazawa, M. Sato, K. Namiki, M. Tsugumi, Depolymerization of polyethylene terephthalate to monomers with supercritical methanol. J. Chem. Eng. Jpn. 30, 342–346 (1997).

[31] D. D. Pham, J. Cho, Low-energy catalytic methanolysis of poly (ethyleneterephthalate). Green Chem. 23, 511–525 (2021).

[32] K. Fukushima, D. J. Coady, G. O. Jones, H. A. Almegren, A. M. Alabdulrahman, F. D. Alsewailem, H. W. Horn, J. E. Rice, J. L. Hedrick, Unexpected efficiency of cyclic amidine catalysts in depolymerizing poly (ethylene terephthalate). J. Polym. Sci. A 51, 1606–1611 (2013).

[33] S. Liu, Z. Wang, L. Li, S. Yu, C. Xie, F. Liu, Butanol alcoholysis reaction of polyethylene terephthalate using acidic ionic liquid as catalyst. J. Appl. Polym. Sci. 130, 1840–1844 (2013).

[34] K. R. Delle Chiaie, F. R. McMahon, E. J. Williams, M. J. Price, A. P. Dove, Dual-catalytic depolymerization of polyethylene terephthalate (PET). Polym. Chem. 11, 1450–1453 (2020).

[35] F. Chen, Q. Zhou, R. Bu, F. Yang, W. Li, Kinetics of poly (ethylene terephthalate) fiber glycolysis in ethylene glycol. Fiber Polym. 16, 1213–1219 (2015).

[36] J. Sutton, G. Grause, A. A. R. Hmayed, S. T. Street, A. P. Dove, J. Wood, Organocatalytic glycolysis of polyethylene terephthalate and product separation by membrane filtration. Chem. Eng. J. 512, 162400 (2025).

[37] F. F. Chen, G. H. Wang, W. Li, F. Yang, Kinetics of glycolysis of poly (ethylene terephthalate) by shrinking-core model. Adv. Mater. Res. 233, 627–631 (2011).

[38] J. W. Chen, L. W. Chen, W. H. Cheng, Kinetics of glycolysis of polyethylene terephthalate with zinc catalyst. Polym. Int. 48, 885–888 (1999).

[39] S. Kaiho, A. A. R. Hmayed, K. R. Delle Chiaie, J. C. Worch, A. P. Dove, Designing thermally stable organocatalysts for poly (ethylene terephthalate) synthesis: Toward a one-pot, closed-loop chemical recycling system for PET. Macromolecules 55, 10628–10639 (2022).

[40] A. J. Spicer, A. Brandolese, A. P. Dove, Selective and sequential catalytic chemical depolymerization and upcycling of mixed plastics. ACS Macro Lett. 13, 189–194 (2024). 38253019 10.1021/acsmacrolett.3c00751 PMC10883033

[41] J. Huang, D. Yan, Q. Zhu, X. Cheng, J. Tang, X. Lu, J. Xin, Depolymerization of polyethylene terephthalate with glycol under comparatively mild conditions. Polym. Degrad. Stab. 208, 110245 (2023).

[42] K. Van Aken, L. Strekowski, L. Patiny, EcoScale, a semi-quantitative tool to select an organic preparation based on economical and ecological parameters. Beilstein J. Org. Chem. 2, 3 (2006). 16542013 10.1186/1860-5397-2-3 PMC1409775

[43] A. Gałuszka, Z. M. Migaszewski, P. Konieczka, J. Namieśnik, Analytical Eco-Scale for assessing the greenness of analytical procedures. Trends Anal. Chem. 37, 61–72 (2012).

[44] Y. Suzuki, T. Kano, T. Tomii, N. Tsuji, A. Matsumoto, Relaxation and amorphous structure of polymers containing rigid fumarate segments. Polymers 14, 4876–4887 (2022). 36433003 10.3390/polym14224876 PMC9692691

[45] M. Sepe, *Dynamic Mechanical Analysis for Plastics Engineering* (William Andrew, 1998).

[46] T. R. Long, R. M. Elder, E. D. Bain, K. A. Masser, T. W. Sirk, J. H. Yu, D. B. Knorr, J. L. Lenhart, Influence of molecular weight between crosslinks on the mechanical properties of polymers formed via ring-opening metathesis. Soft Matter 14, 3344–3360 (2018). 29658546 10.1039/c7sm02407j

[47] A. Oussai, Z. Bártfai, L. Kátai, Development of 3D printing raw materials from plastic waste. A case study on recycled polyethylene terephthalate. Appl. Sci. 11, 7338 (2021).

[48] T. Kuhnt, F. L. Morgan, M. B. Baker, L. Moroni, An efficient and easily adjustable heating stage for digital light processing set-ups. Addit. Manuf. 46, 102102 (2021).

[49] Y. He, N. Li, Z. Xiang, Y. Rong, L. Zhu, X. Huang, Natural polyphenol as radical inhibitors used for DLP-based 3D printing of photosensitive gels. Mater. Today Commun. 33, 104698 (2022).

[50] T. M. Lammens, M. C. R. Franssen, E. L. Scott, J. P. M. Sanders, Synthesis of biobased N-methylpyrrolidone by one-pot cyclization and methylation of γ-aminobutyric acid. Green Chem. 12, 1430–1436 (2010).

[51] O. Clavilier, D. Foy, F. Byrne, The solvent miscibility table updated: Miscibility and potential applications of green solvents. Green Chem. 27, 12151–12159 (2025).

[52] J. W. Seo, G. M. Kim, Y. Choi, J. M. Cha, H. Bae, Improving printability of digital-light-processing 3D bioprinting via photoabsorber pigment adjustment. Int. J. Mol. Sci. 23, 5428 (2022). 35628238 10.3390/ijms23105428 PMC9143265

[53] Z. Wang, Q. Lu, X. Li, Y. Zhou, Y. Xiao, M. Lang, Digital light processing of customized elastic scaffolds by efficient thiol-yne crosslinking. Eur. Polym. J. 202, 112586 (2024).

[54] G. Chure, J. Cremer, hplc-py: A python utility for rapid quantification of complex chemical chromatograms. J. Open Source Softw. 9, 6270 (2024).

[55] D. Tillier, H. Lefebvre, M. Tessier, J. C. Blais, A. Fradet, High temperature bulk reaction between poly (ethylene terephtalate) and lactones: 1H NMR and SEC/MALDI-TOF MS study. Macromol. Chem. Phys. 205, 581–592 (2004).

[56] Ecoscale calculator (2006); https://ecoscale.cheminfo.org/calculator.

