# Supplementary material for: Low-temperature alcoholysis as a strategy for polyethylene terephthalate recycling and upcycling
Source: Sci Adv. 2026 Jul 17;12(29):eaed4780. doi: 10.1126/sciadv.aed4780 (PMC13378564; doi:10.1126/sciadv.aed4780)
Supplement: Supplementary file 1 — Figs. S1 to S66 Tables S1 to S5 Supplementary Text References [file sciadv.aed4780_sm.pdf]

Science Advances
AAAS

Supplementary Materials for

Low-temperature alcoholysis as a strategy for polyethylene terephthalate recycling and upcycling

Matt J. Price *et al.*

Corresponding author: Andrew P. Dove, a.dove@bham.ac.uk

Sci. Adv. **12**, eaed4780 (2026)
DOI: 10.1126/sciadv.aed4780

This PDF file includes:

Figs. S1 to S66
Tables S1 to S5
Supplementary Text
References

Glycolysis kinetics

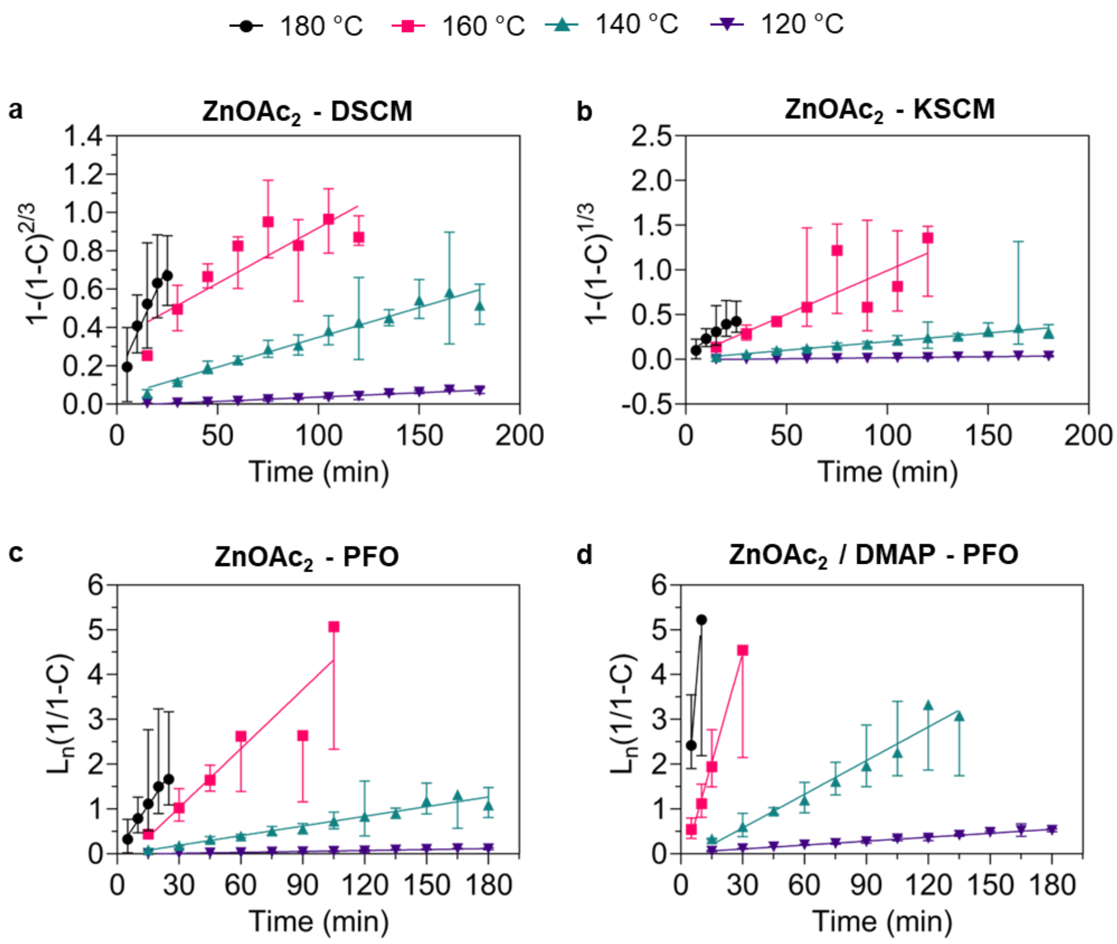

Fig. S1.

Kinetics data for (a-c) zinc acetate catalyzed glycolysis fitted to (a) the Diffusion Shrinking Core Model (DSCM), (b) the Kinetic Shrinking Core Model (KSCM), (c) the Pseudo First Order model (PFO), and (d) zinc acetate / DMAP catalyzed glycolysis fitted to the PFO model. Data analysis was conducted using the 'simple linear regression' function of GraphPad Prism.

Table S1.

DSCM, KSCM, and PFO models fitted to glycolysis reactions with apparent rate constants (k_{app}) for zinc acetate and zinc acetate / DMAP catalyzed glycolysis at temperatures between 120 °C and 180 °C. Data analysis was conducted using the 'simple linear regression' function of GraphPad Prism.

Catalyst / Model	Temperature (°C)	k_{app} ($\times 10^{-2}$ min^{-1})	σ (k_{app}) ($\times 10^{-2}$ min^{-1})	R^2
Zinc Acetate DSCM	180	2.35	0.36	0.93
	160	0.58	0.14	0.74
	140	0.31	0.019	0.96
	120	0.045	0.0023	0.97
Zinc Acetate KSCM	180	1.62	0.19	0.96
	160	0.97	0.27	0.69
	140	0.19	0.012	0.96
	120	0.023	0.0012	0.97
Zinc Acetate PFO	180	6.77	0.58	0.98
	160	4.40	0.84	0.87
	140	0.72	0.050	0.95
	120	0.070	0.0038	0.97
Zinc Acetate / DMAP PFO	180	56.13	-	>0.99
	160	16.33	0.78	>0.99
	140	2.50	0.19	0.96
	120	0.29	0.0080	>0.99

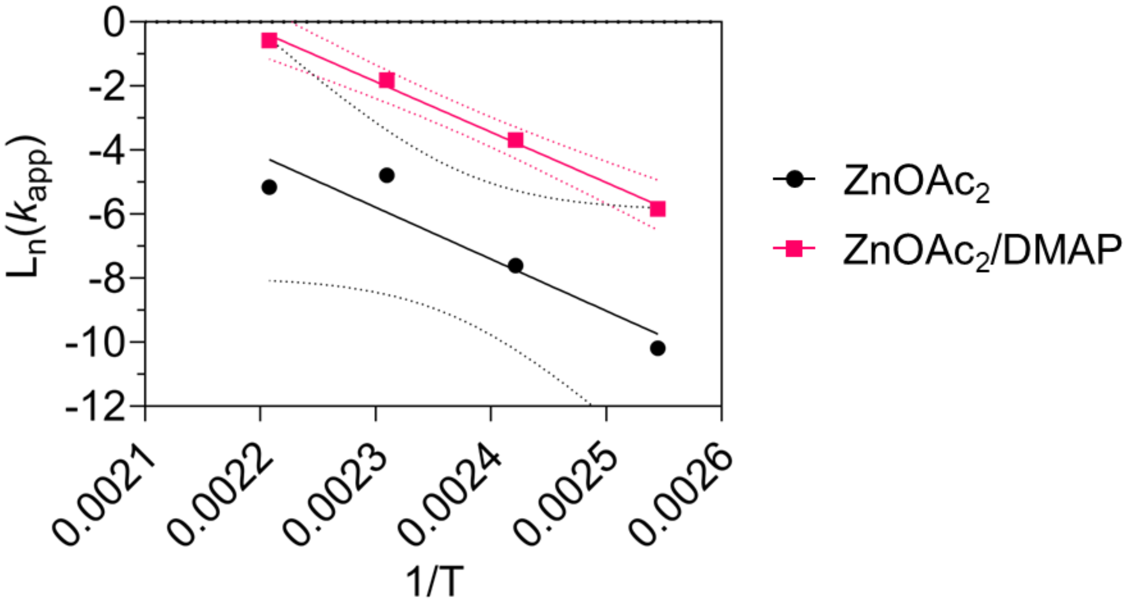

Fig. S2.

Arrhenius plot for the determination of the activation energy for both zinc acetate and zinc acetate / DMAP catalyzed glycolysis. Zinc acetate equation: Y = -16173*X + 31.41 ($R^2 = 0.88$). Zinc acetate / DMAP equation: Y = -15758*X + 34.38 ($R^2 = 0.99$). The 95 % confidence interval is displayed on the graph as a dotted line. Data analysis was conducted using the 'simple linear regression' function of GraphPad Prism.

Alcoholysis kinetics

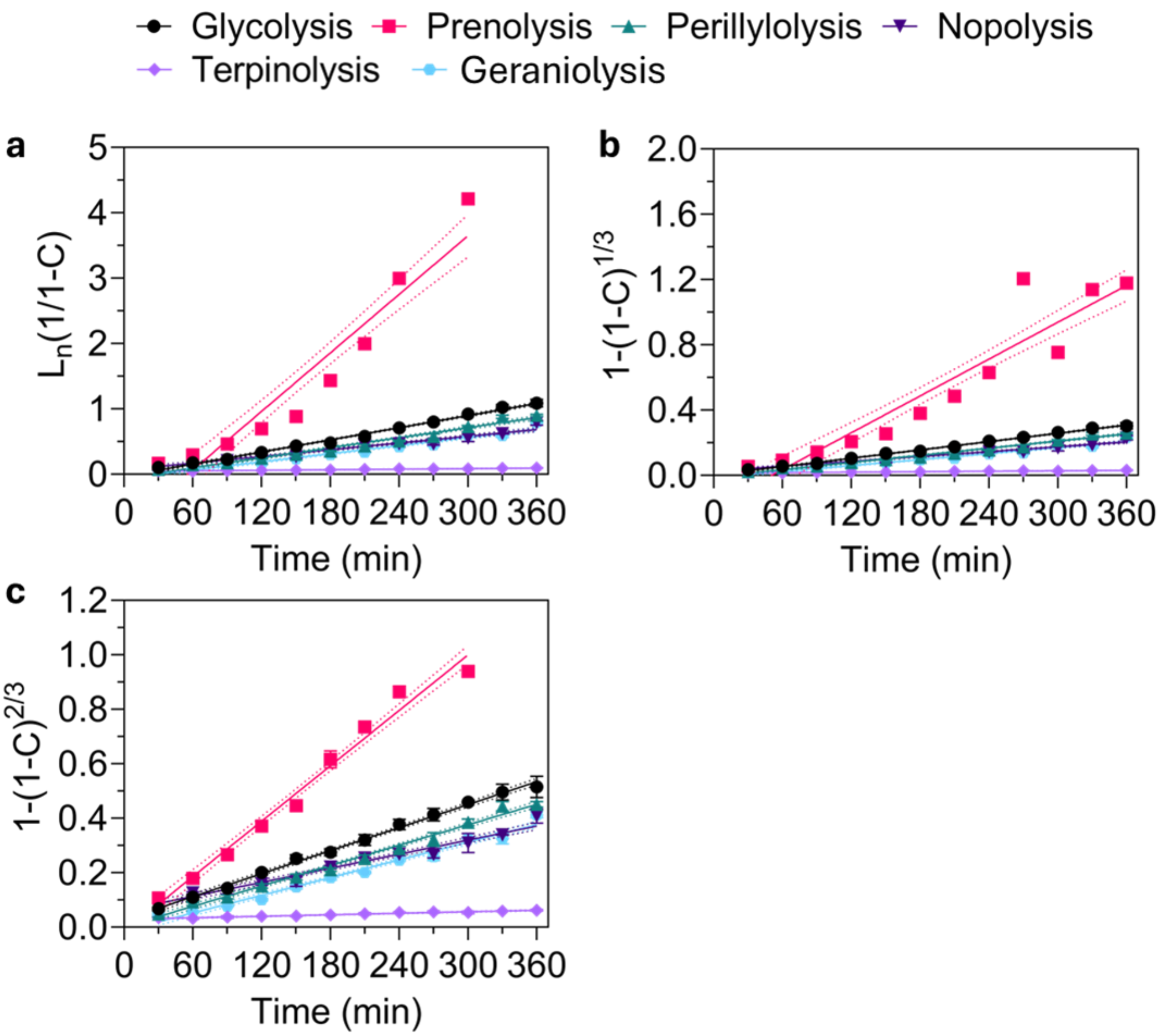

Fig. S3.

Kinetics data for zinc acetate / DMAP catalyzed alcoholysis at 120 °C fitted to (a) the PFO model, (b) the KSCM, and (c) the DSCM. Data analysis was conducted using the 'simple linear regression' function of GraphPad Prism.

Table S2.

Kinetics models fitted to zinc acetate / DMAP catalyzed alcoholysis kinetics at 120 °C: PFO Model, KSCM, and DSCM with apparent rate constants (k_{app}). Data analysis was conducted using the 'simple linear regression' function of GraphPad Prism.

**Model**	**Alcohol**	k_{app} ($\times 10^{-2}$ min^{-1})	σ (k_{app}) ($\times 10^{-2}$ min^{-1})	R^2
PFO	Ethylene Glycol	0.31	0.0058	0.99
	Prenol	1.49	0.094	0.91
	Perillyl Alcohol	0.26	0.0081	0.97
	Nopol	0.17	0.0077	0.94
	Terpineol	0.014	0.00086	0.89
	Geraniol	0.21	0.0078	0.95
KSCM	Ethylene Glycol	0.085	0.0015	0.99
	Prenol	0.38	0.024	0.88
	Perillyl Alcohol	0.073	0.0019	0.98
	Nopol	0.049	0.0021	0.94
	Terpineol	0.0047	0.00028	0.89
	Geraniol	0.061	0.0019	0.97
DSCM	Ethylene Glycol	0.14	0.0030	0.99
	Prenol	0.34	0.0099	0.98
	Perillyl Alcohol	0.12	0.0028	0.98
	Nopol	0.086	0.0034	0.95
	Terpineol	0.0092	0.00056	0.89
	Geraniol	0.11	0.0031	0.97

Benchmarking of Zn(OAc)$_2$/DMAP Against Comparable Low-Temperature Systems

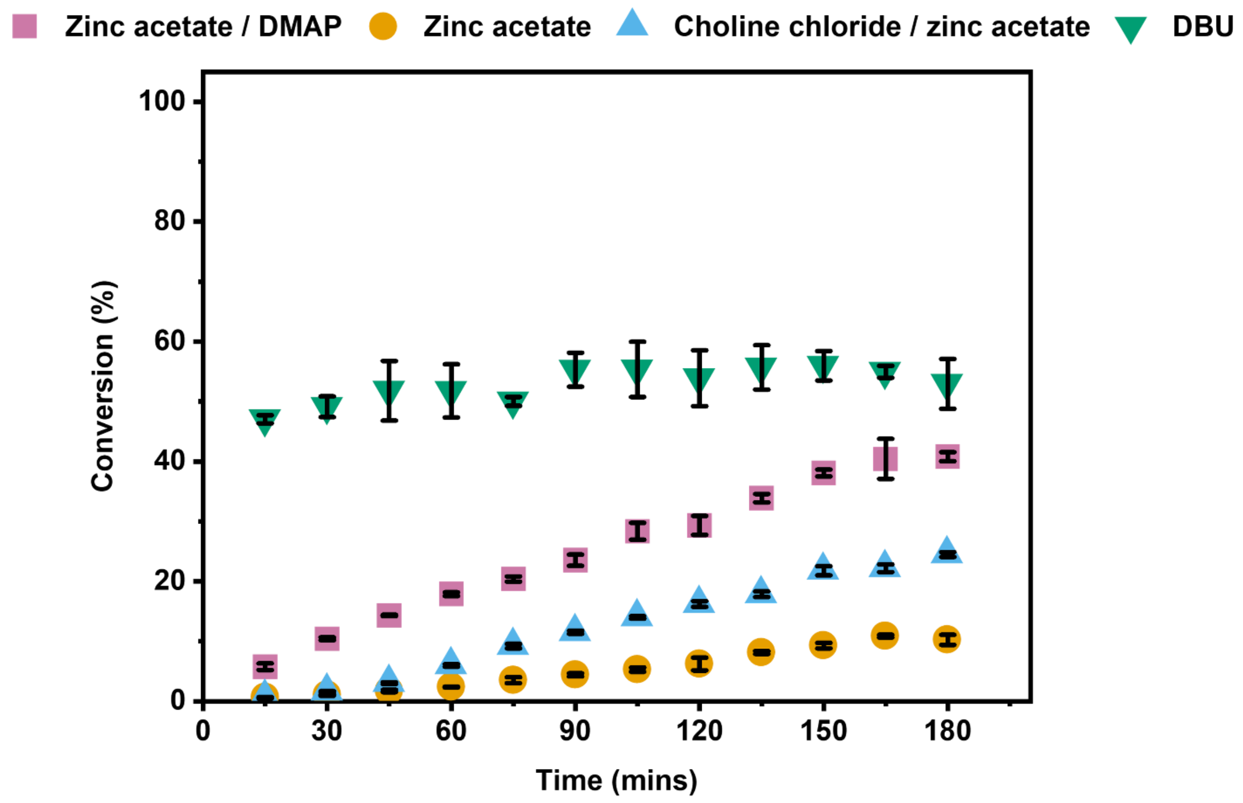

Fig. S4.
Plots of conversion against time for glycolysis of PET catalyzed by Zn(OAc)$_2$/DMAP compared to comparable systems from the literature.(*32, 41*)

NMR spectra

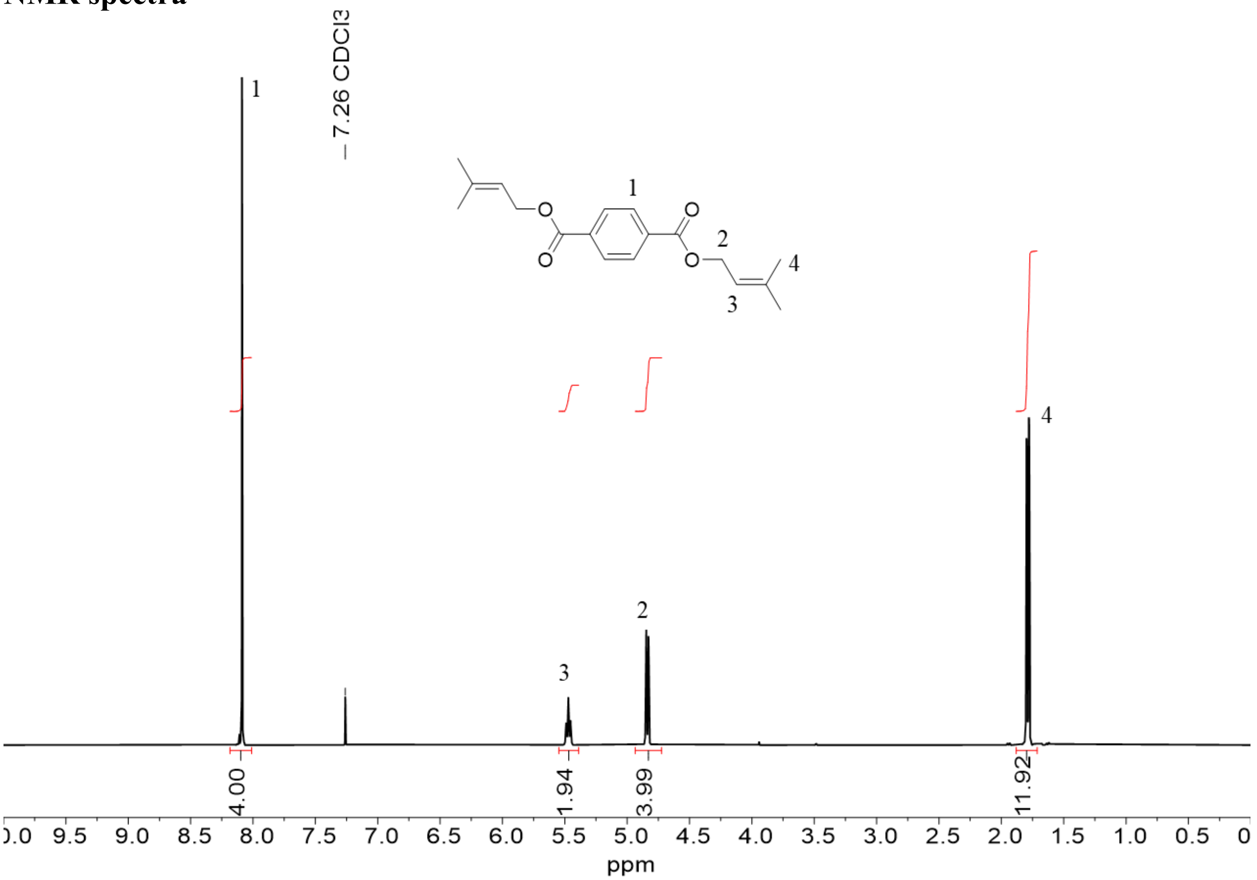

Fig. S5.

^{1}H NMR ($CDCl_3$, 298 K, 400 MHz) spectrum of bis(prenol) terephthalate (BPreT).

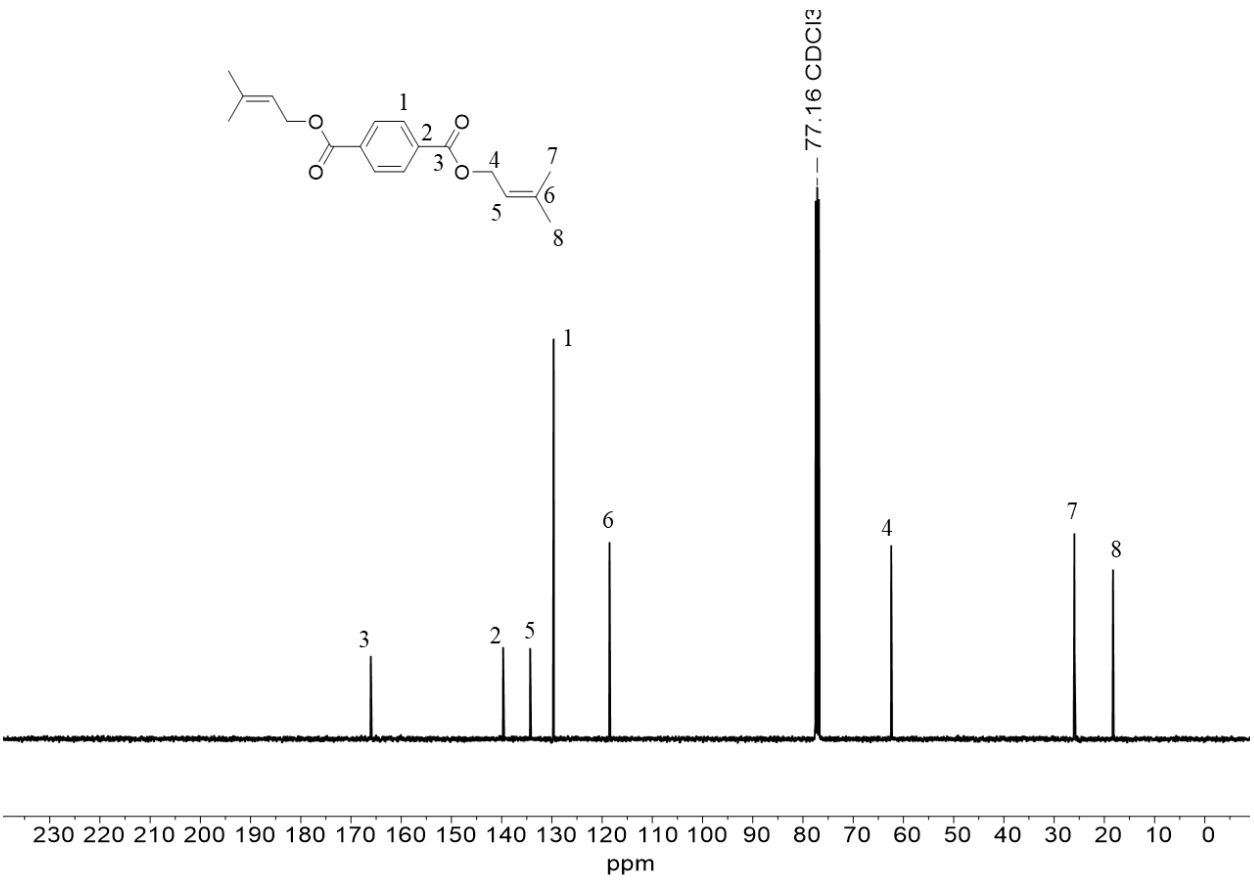

Fig. S6.
^{13}C NMR ($CDCl_3$, 298 K, 100 MHz) spectrum of bis(prenol) terephthalate (BPreT).

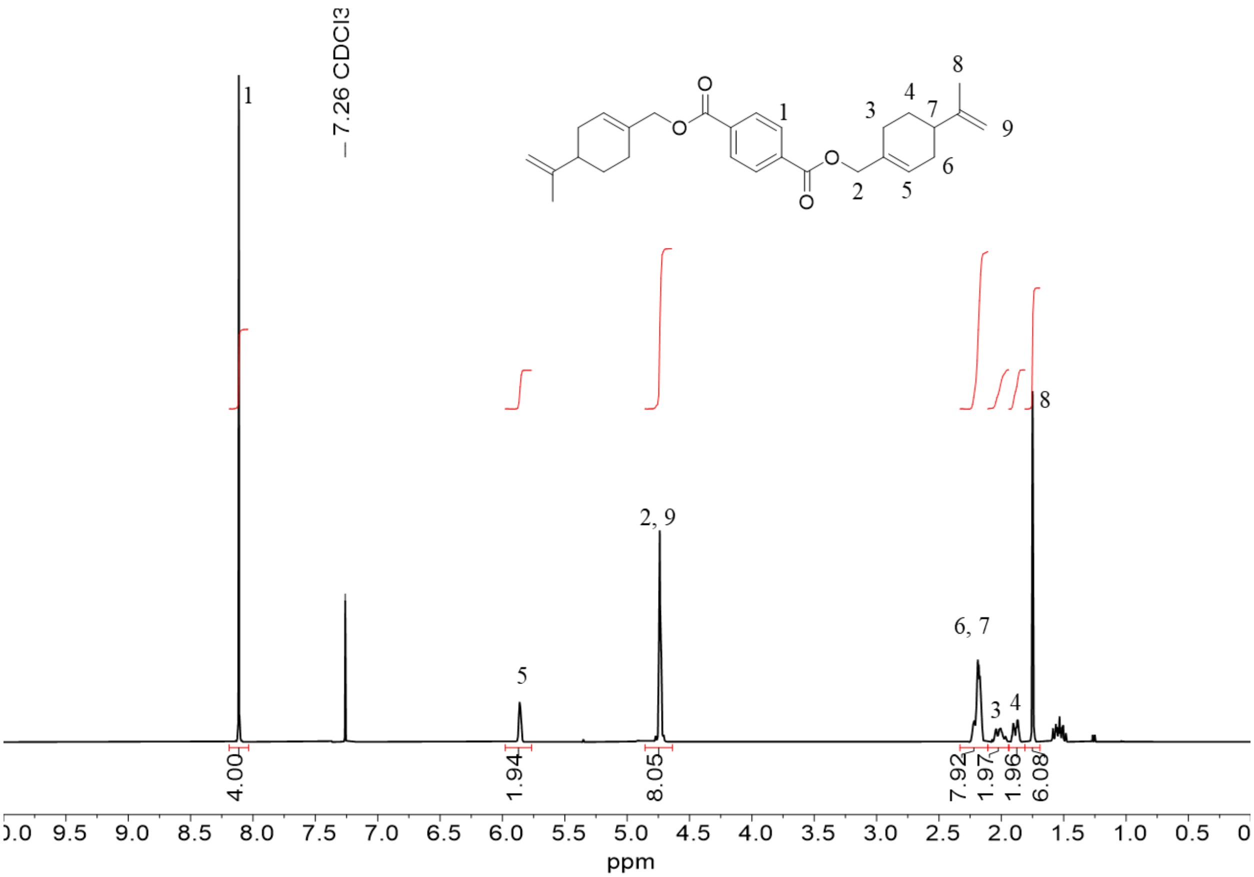

Fig. S7.

^{1}H NMR ($CDCl_3$, 298 K, 400 MHz) spectrum of bis(perillyl) terephthalate (BPerT).

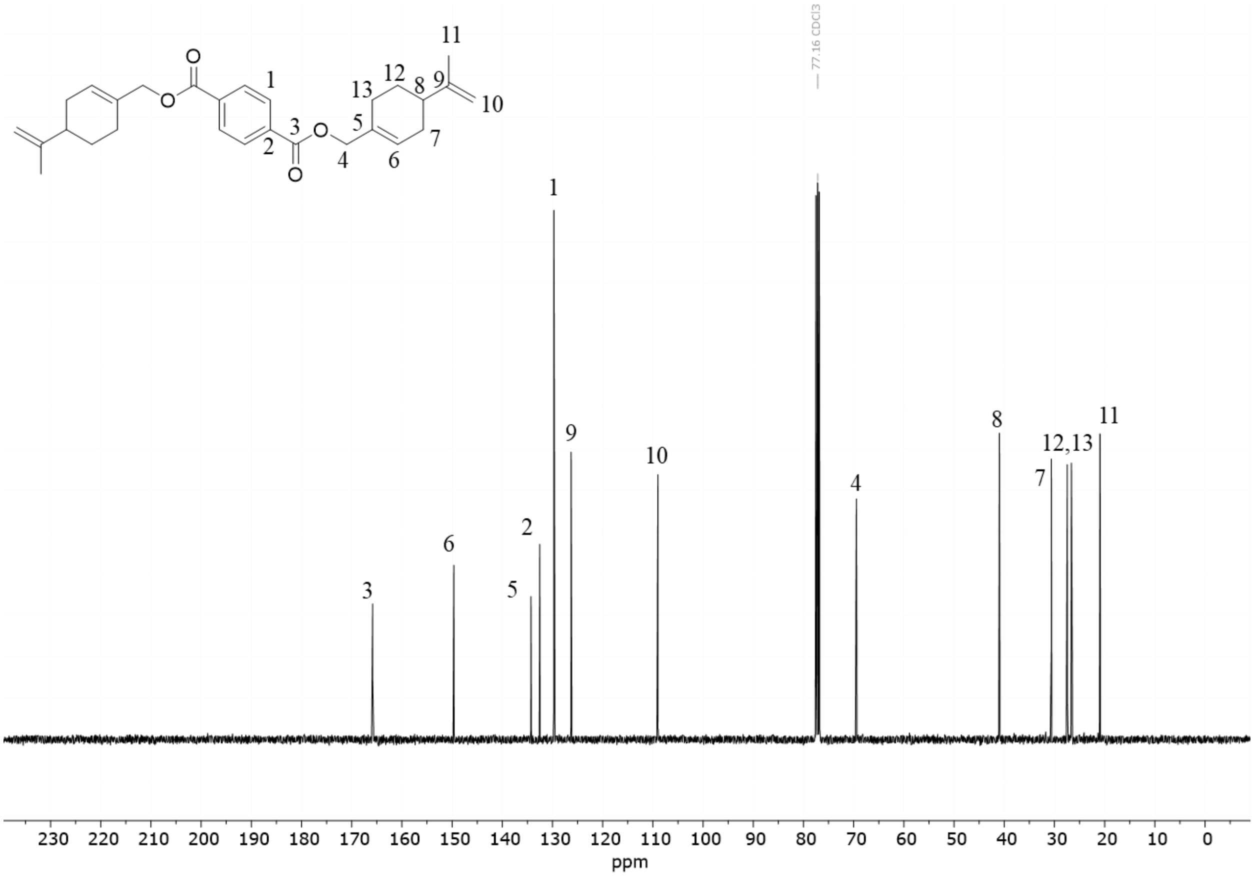

Fig. S8.
^{13}C NMR ($CDCl_3$, 298 K, 100 MHz) spectrum of bis(perillyl) terephthalate (BPerT).

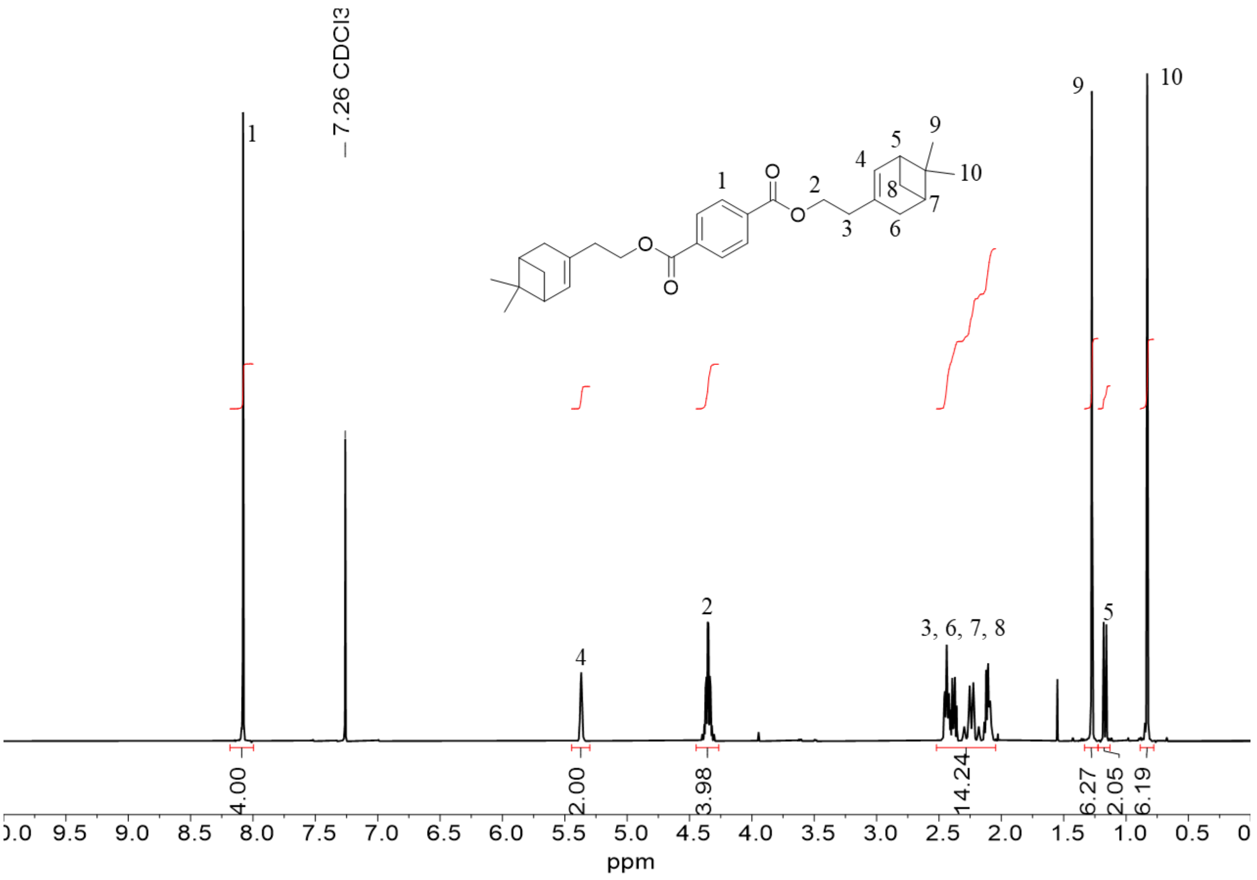

Fig. S9.

^{1}H NMR ($CDCl_3$, 298 K, 400 MHz) spectrum of bis(nopyl) terephthalate (BNopT).

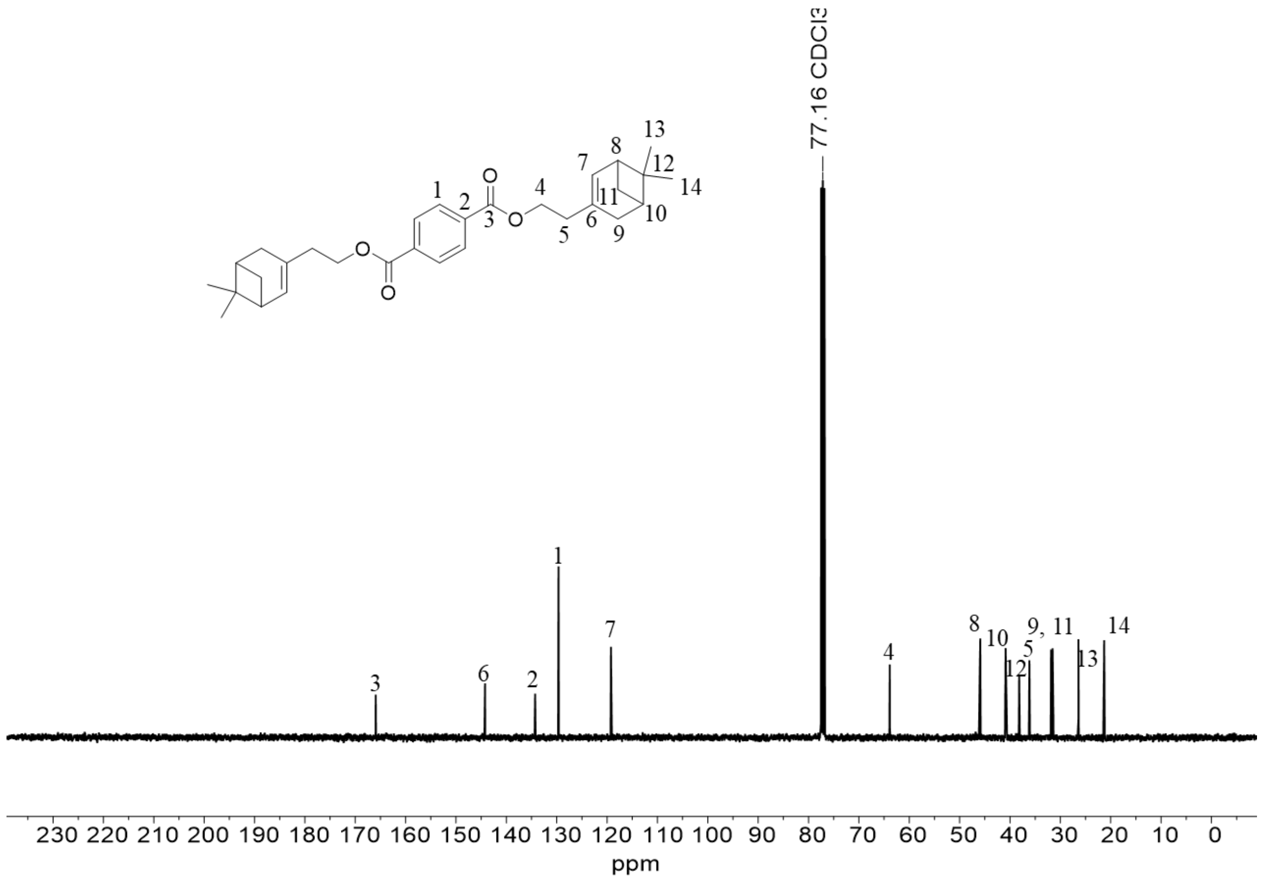

Fig. S10.
^{13}C NMR ($CDCl_3$, 298 K, 100 MHz) spectrum of bis(nopyl) terephthalate (BNopT).

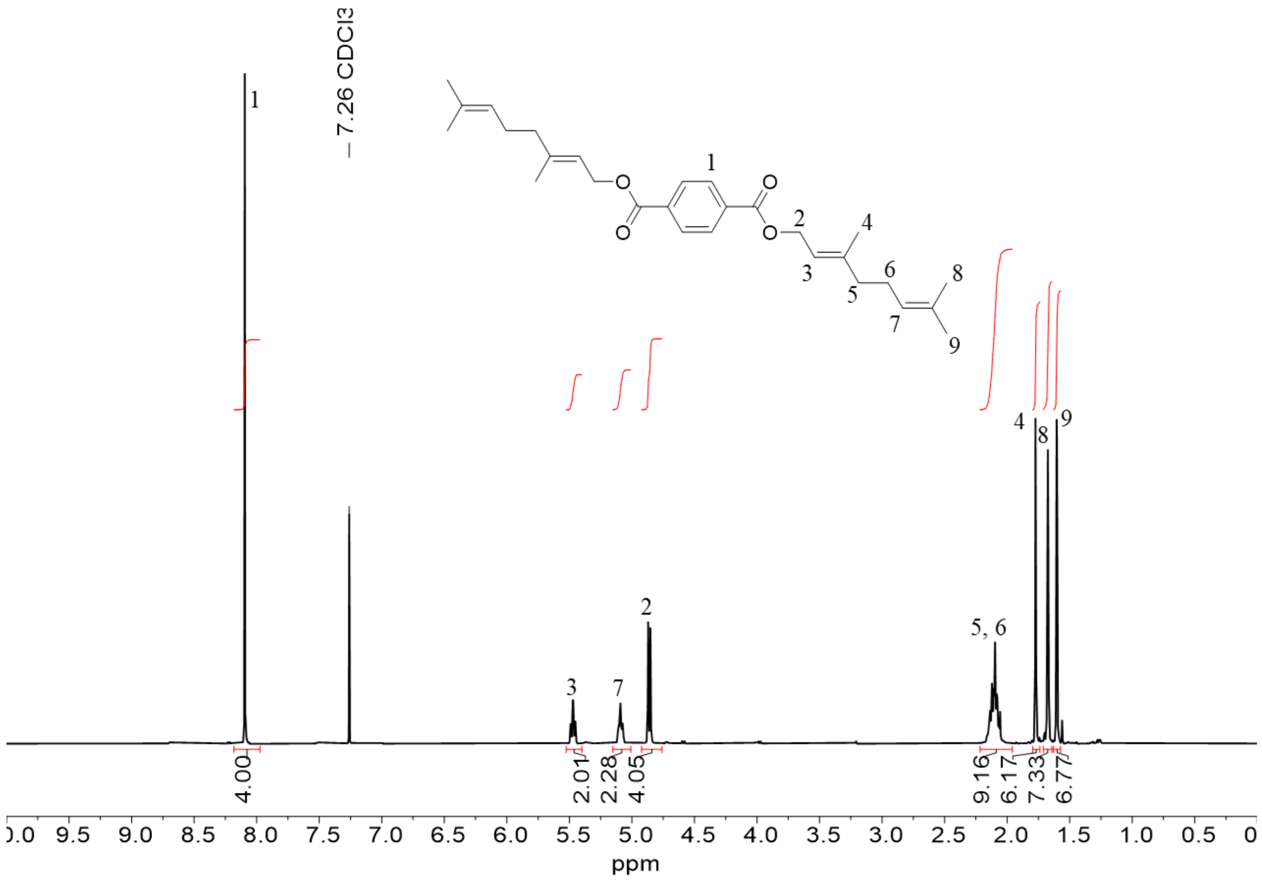

Fig. S11.

^{1}H NMR ($CDCl_3$, 298 K, 400 MHz) spectrum of bis(geranyl) terephthalate (BGerT).

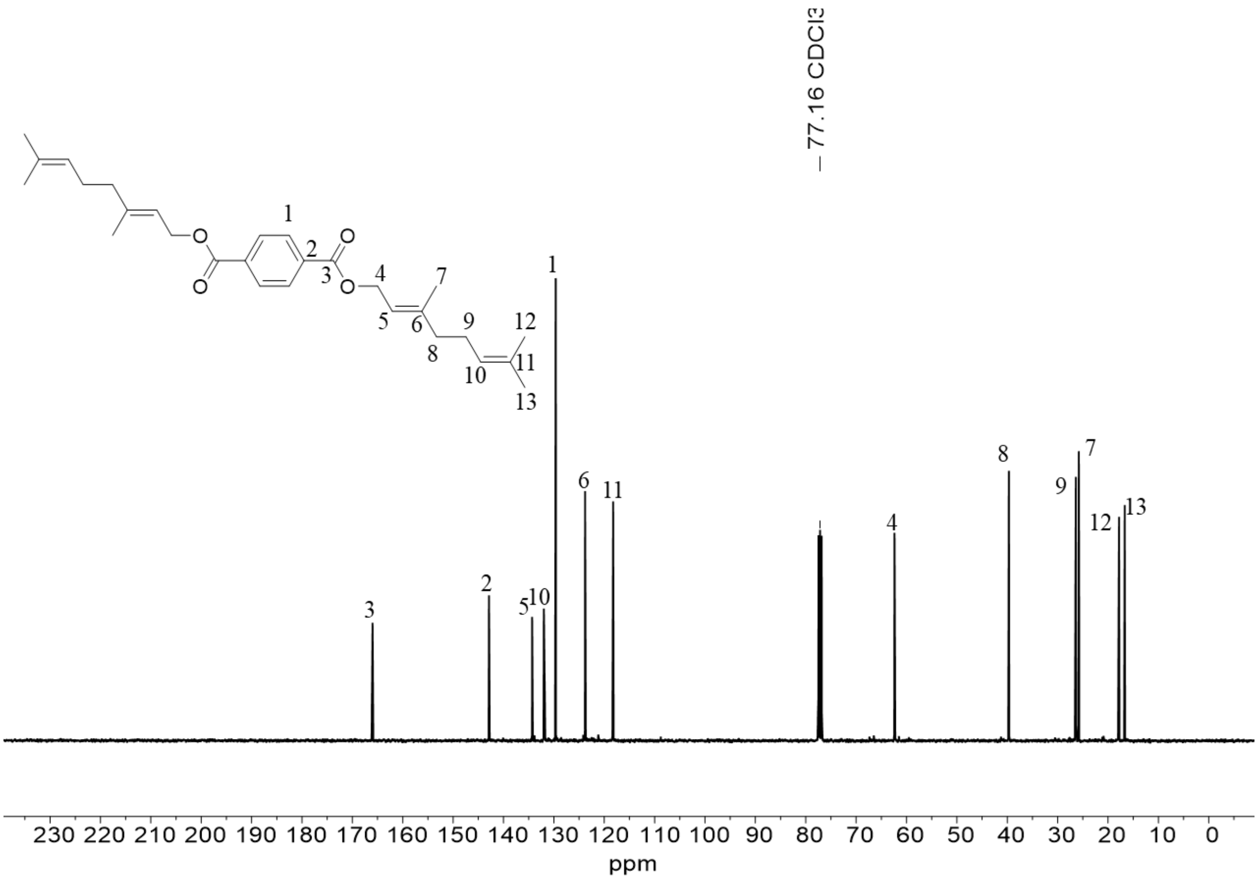

Fig. S12.

^{13}C NMR ($CDCl_3$, 298 K, 100 MHz) spectrum of bis(geranyl) terephthalate (BGerT).

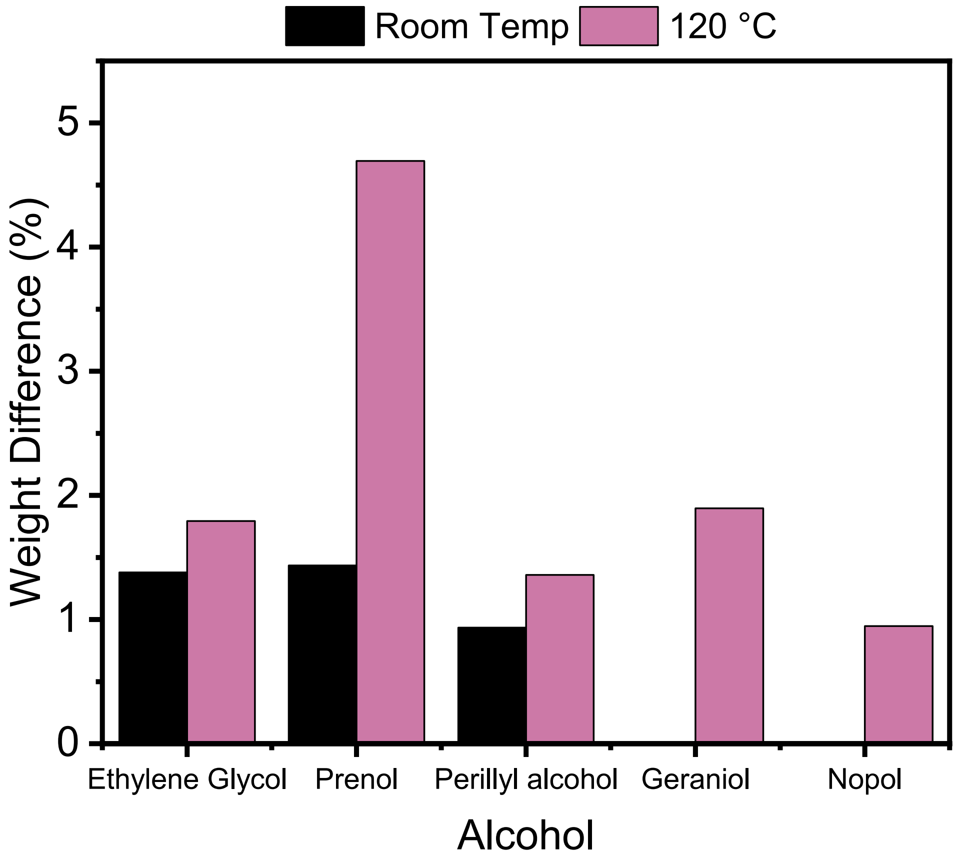

Fig. S13.

Swelling tests conducted with PET pellets (*ca*. 2 mm^3) immersed in alcohols showing activity for PET depolymerization specified in Fig. 2 for 24 h.

Contact Angle Measurements of Ethylene Glycol and Prenol on the Surface of PET

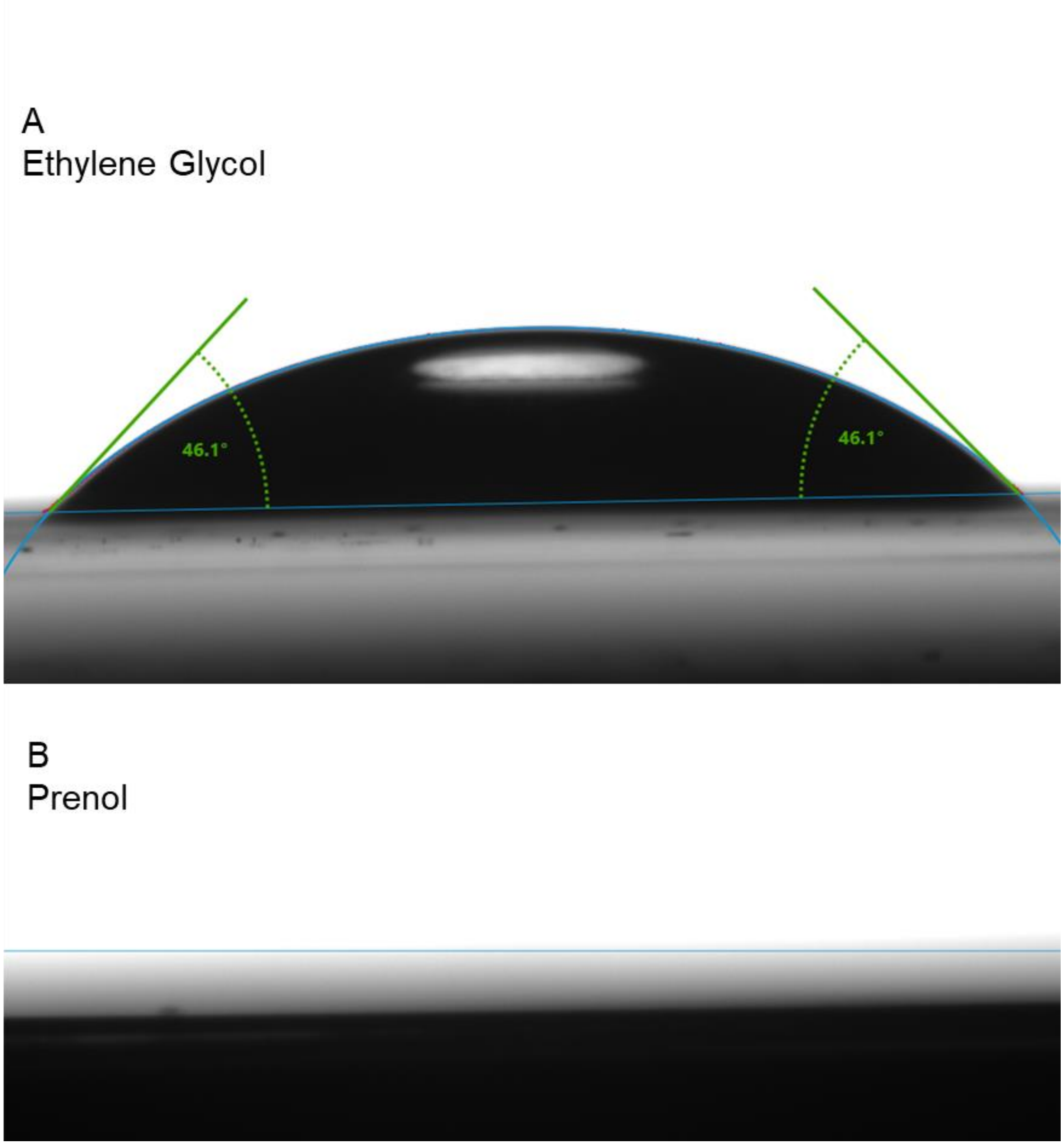

Fig. S14.
Photographs of droplets of (A) ethylene glycol and (B) prenol on the surface of a PET film, showing contact angle measurements. Prenol was observed to instantaneously wet the surface completely.

DMTA of PET Swollen in Prenol and Ethylene Glycol

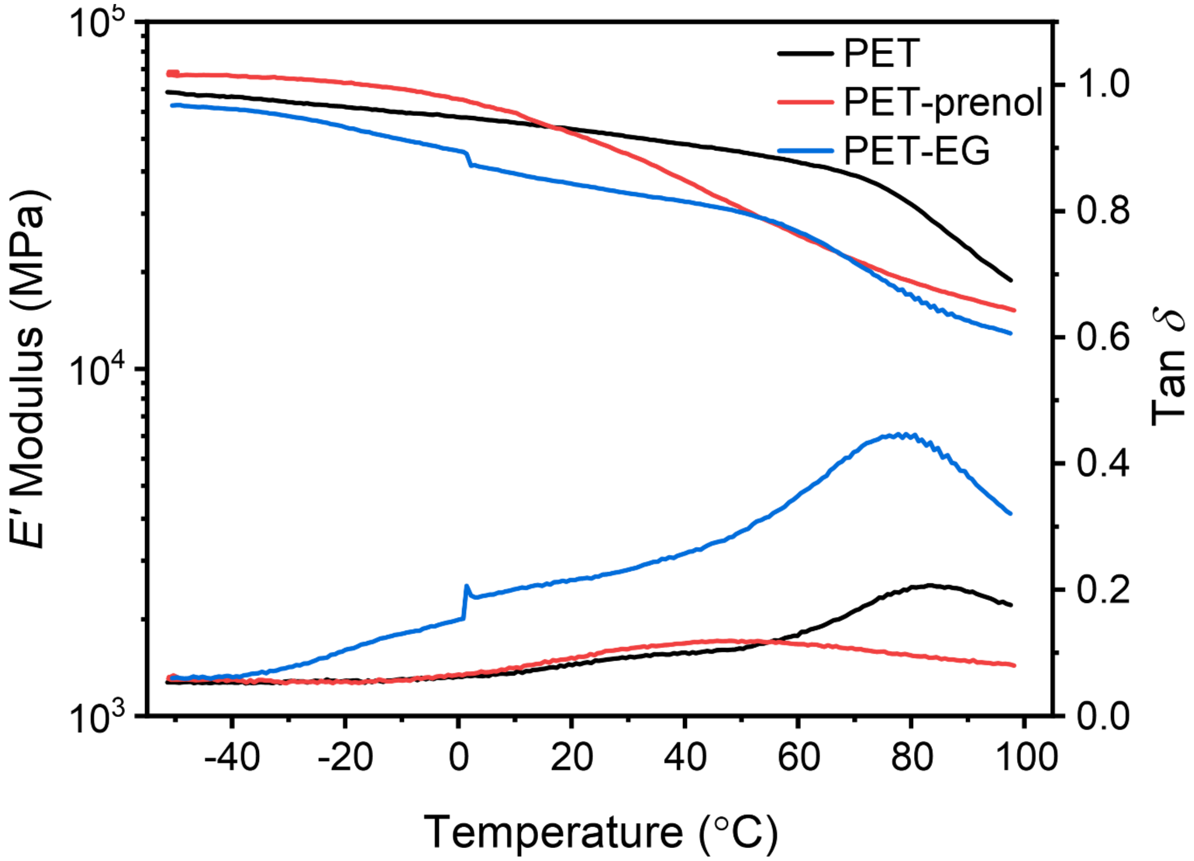

Fig. S15.
DMTA thermogram of PET films swollen in prenol and ethylene glycol.

Mixed Alcohol Depolymerization

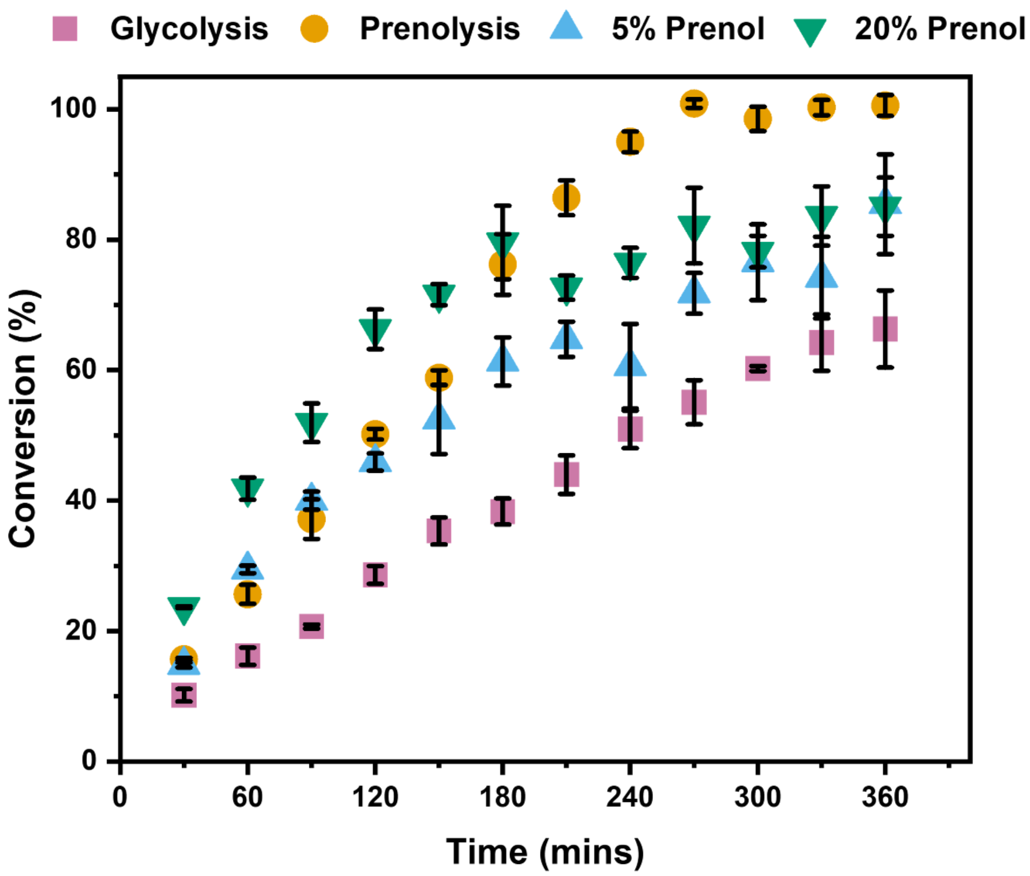

Fig. S16.
Plot of conversion against time for $Zn(OAc)_2$/DMAP-catalyzed alcoholysis of PET using neat ethylene glycol, neat prenol, and ethylene glycol with 5 and 20 mol% prenol.

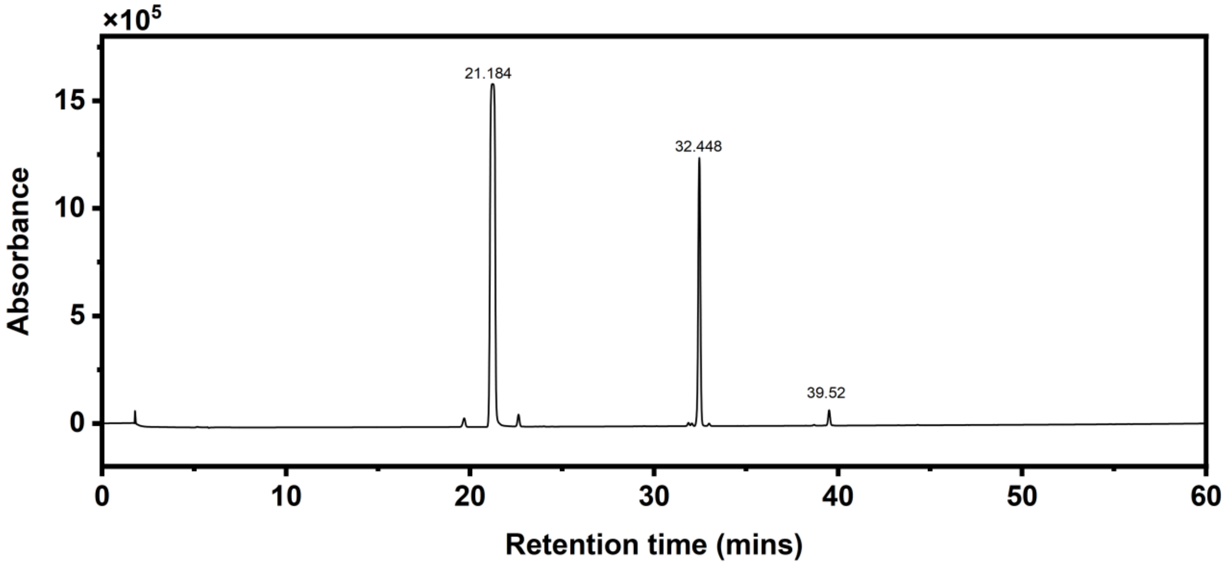

Fig. S17.
HPLC chromatogram of BHET standard used for peak identification.

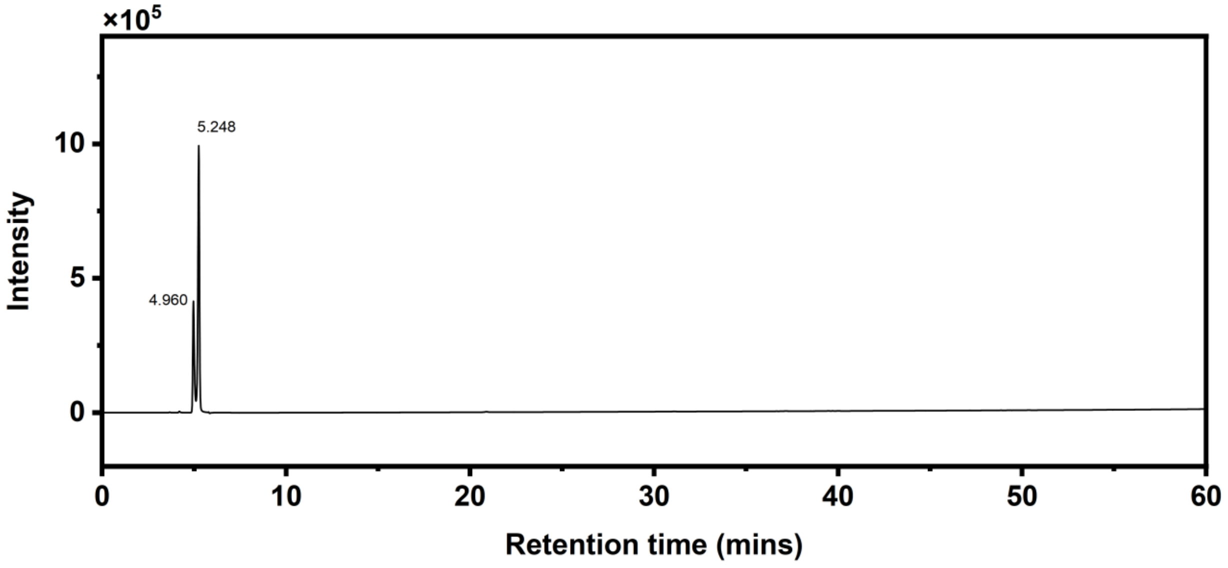

Fig. S18.
HPLC chromatogram of DMAP used for peak identification.

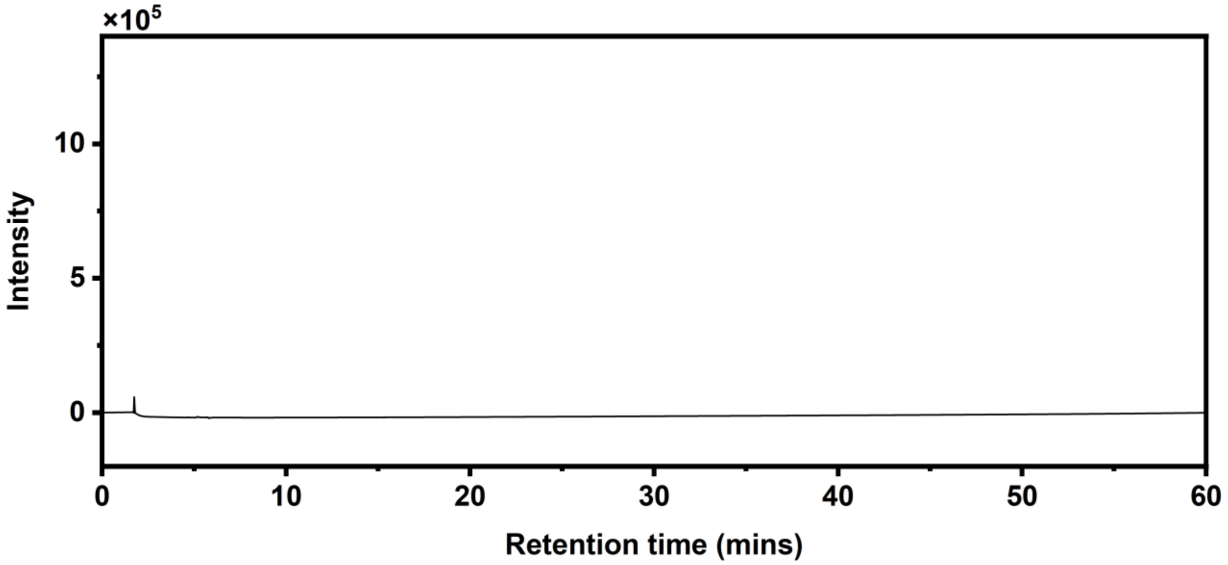

Fig. S19.
HPLC chromatogram of ethylene glycol used for peak identification.

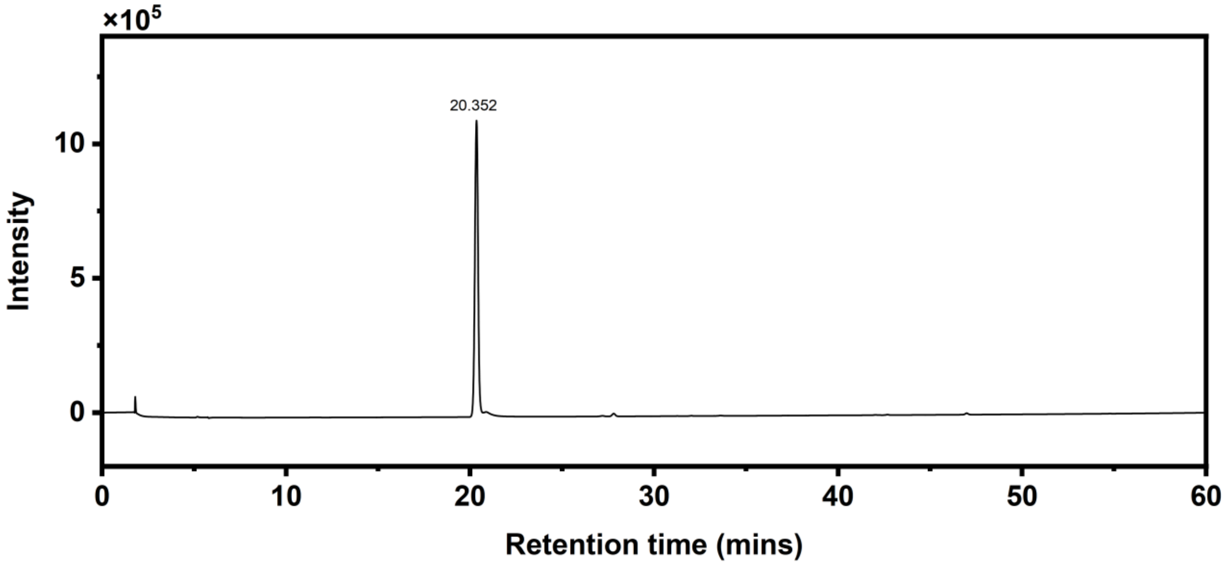

Fig. S20.
HPLC chromatogram of prenol used for peak identification.

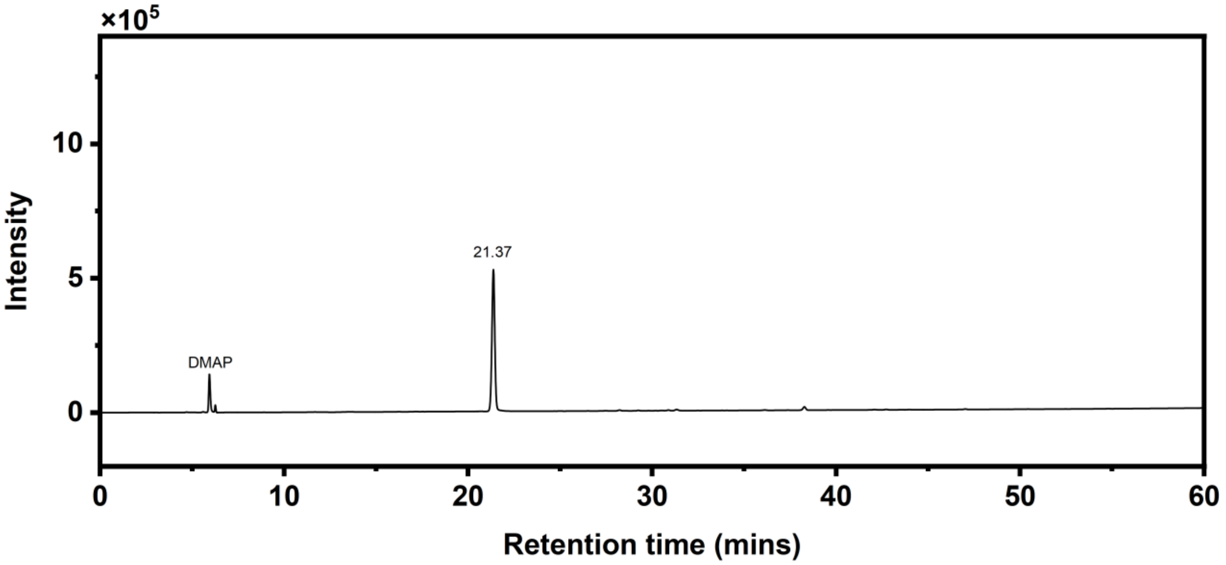

Fig. S21.
HPLC chromatogram of prenol following treatment with $ZnOAc_2$ / DMAP at 120 °C for 6 h used for peak identification.

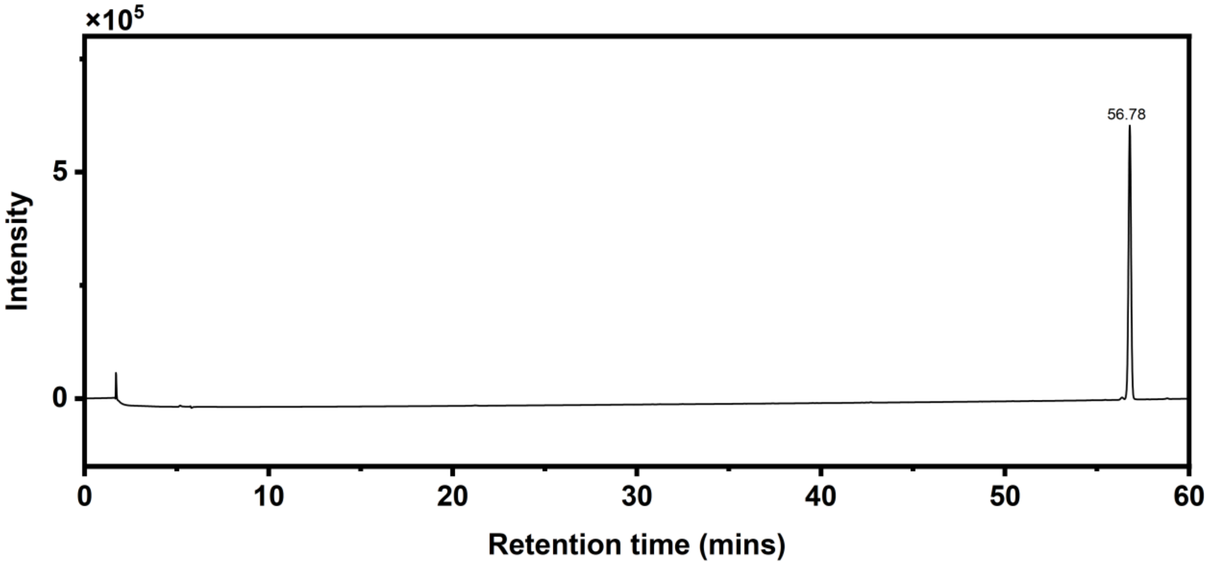

Fig. S22.
HPLC chromatogram of BPreT used for peak identification.

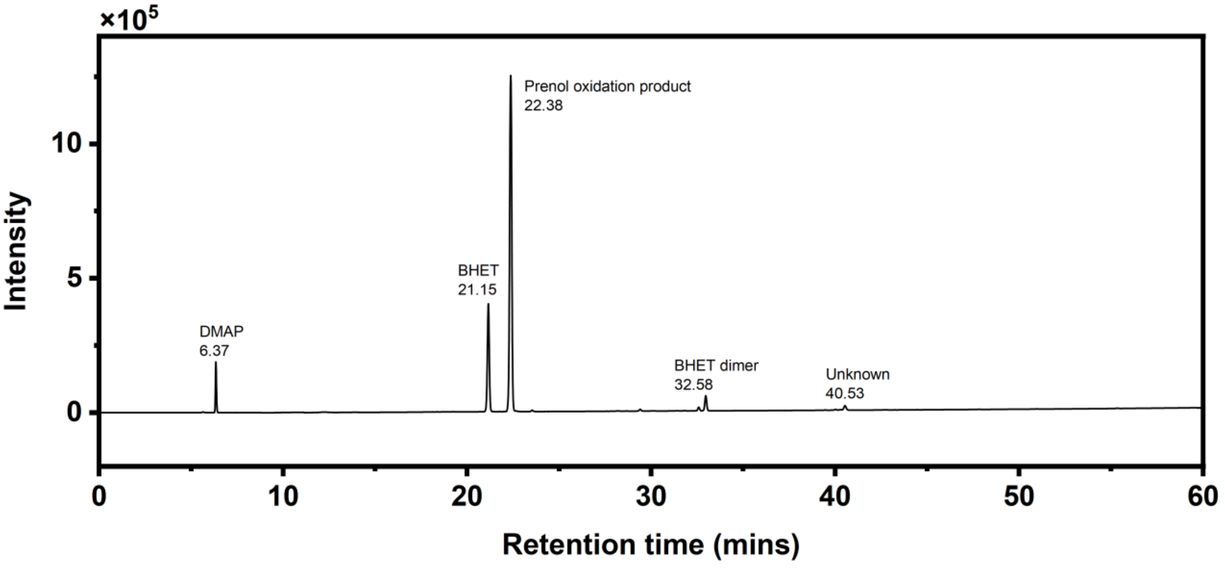

Fig. S23.

HPLC chromatogram of glycolysis spiked with 5% prenol after 6 h with peaks assigned according to Fig. S17-S22. The unknown peak may correspond to a mixed terephthalate as the retention time is halfway between BHET and BPreT or to BHET trimer.

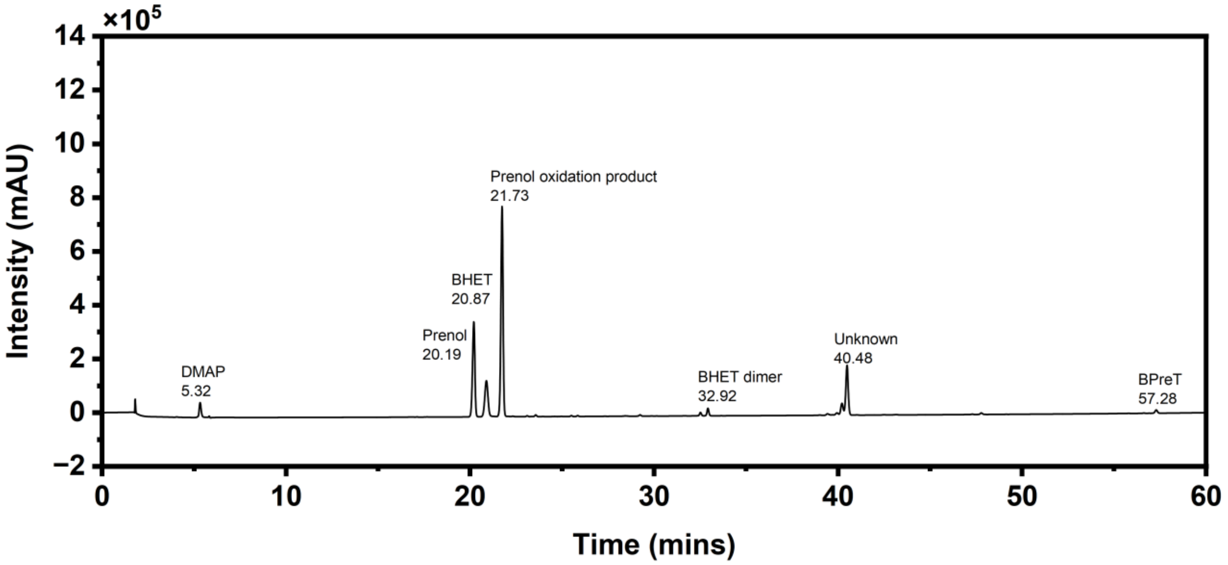

Fig. S24.

HPLC chromatogram of glycolysis spiked with 20% prenol after 6 h with peaks assigned according to Fig. S17-S22. The unknown peak may correspond to a mixed terephthalate as the retention time is halfway between BHET and BPreT or to BHET trimer.

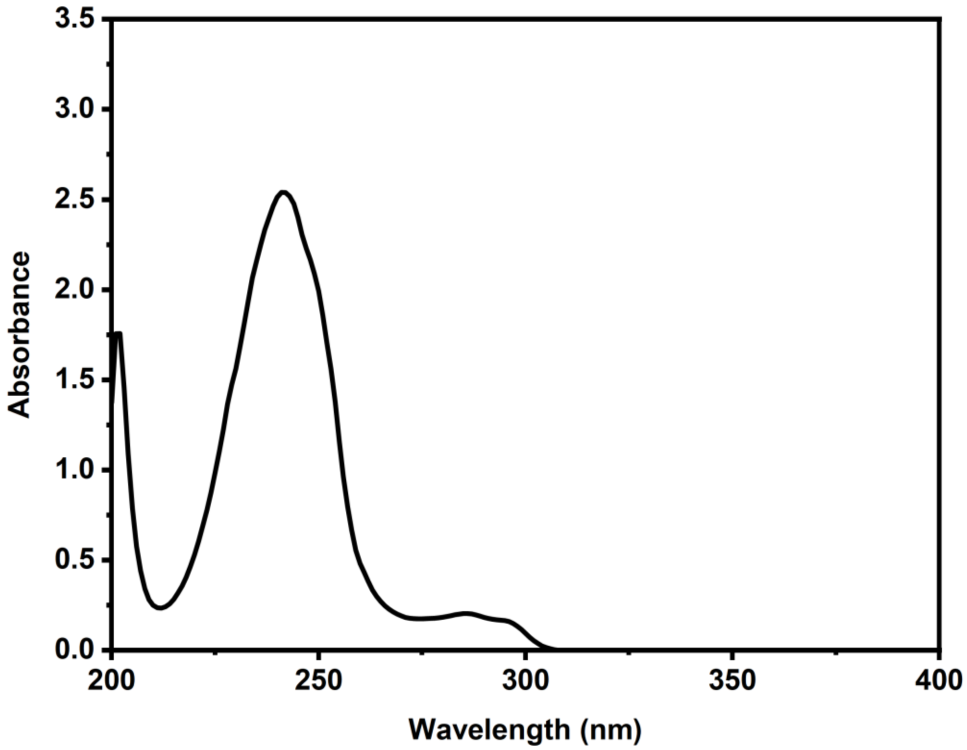

Fig. S25.

UV-vis spectrum of BHET at a concentration of 9.02×10^{-5} mol L^{-1} used to derive the molar absorptivity coefficient at 254 nm.

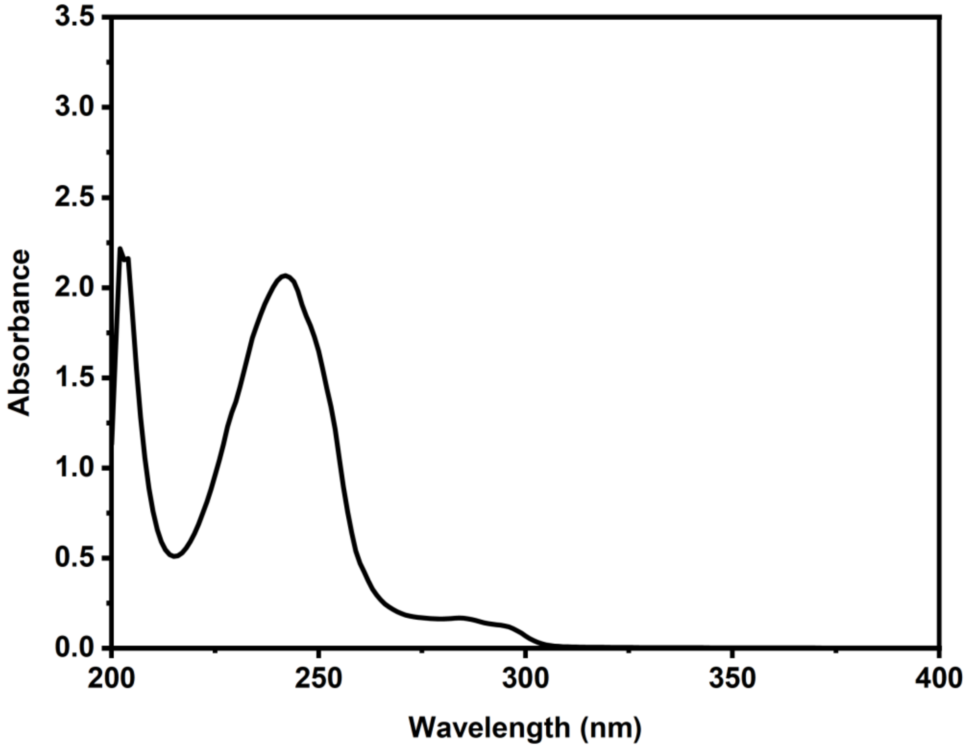

Fig. S26.

UV-vis spectrum of BPreT at a concentration of 9.13×10^{-5} mol L^{-1} used to derive the molar absorptivity coefficient at 254 nm.

Table. S3.

Percentages of monomeric products from glycolysis reactions spiked with prenol (Fig. S16) using UV spectra of BHET and BPreT (Fig. S25 and S26) derived from peak areas on HPLC chromatograms (Fig. S23 and S24).

	**Molar absorptivity coefficient calculation**			
254 nm	BHET	BPreT		Beer Lambert Law
A =	1.377342	1.213898		*A* = *e***c***l*
c =	9.02E-05	9.13E-05		*e*= *A*/*c*
e =	1.53E+04	1.33E+04		
	Assume mixed product has average coefficient			
	1.43E+04			
	**UV chromatogram peak areas**			
	BHET	Mix	BPreT	
5% prenol	48082.95	2384.406	0	
20% prenol	26600.43	37123.96	1819.13	
	**Peak area / coefficient**			
	BHET	Mix	BPreT	
5% prenol	3.15031	0.166965	0	
20% prenol	1.742813	2.599562	0.136789	
	**Ratio**			
	BHET	Mix	BPreT	
5% prenol	1	0.053	0	
20% prenol	1	1.491589	0.078488	
	**Percentages**			
	BHET	Mix	BPreT	
5% prenol	94.9668	5.033204	0	
20% prenol	38.90934	58.03676	3.053902	

Depolymerization of Post-Consumer Waste

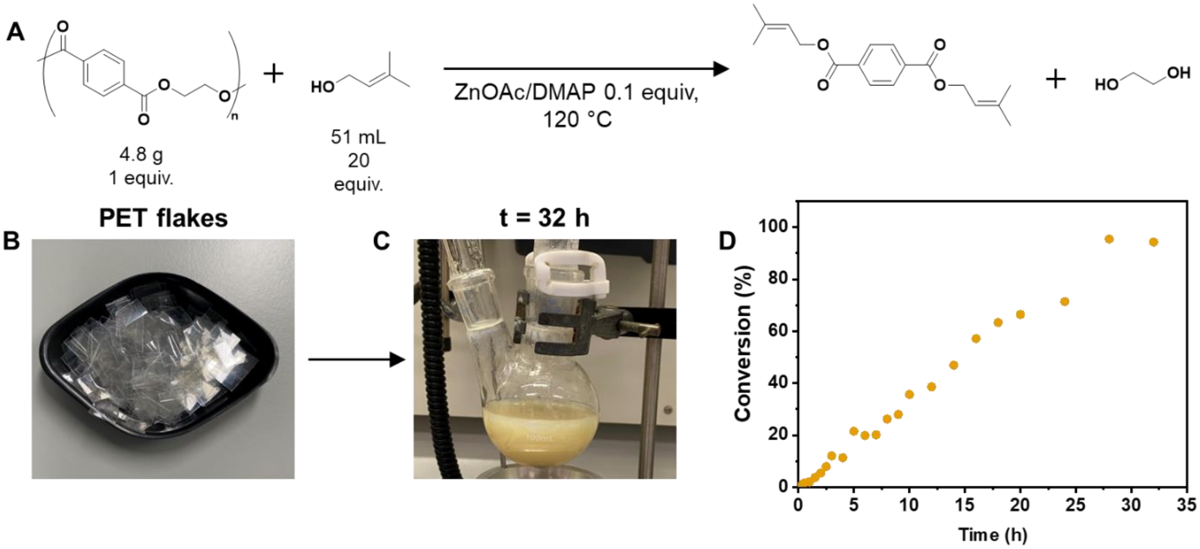

Fig. S27.
(A) Reaction scheme for depolymerization of waste PET drinks bottle. (B) Photograph of flaked PET drinks bottle before depolymerization. (C) Photograph of reaction mixture containing depolymerized post-consumer PET. (D) Plot of conversion against time for depolymerization reaction, depicting the reaction reaching 95% conversion.

Depolymerization with Varying Prenol Equivalents

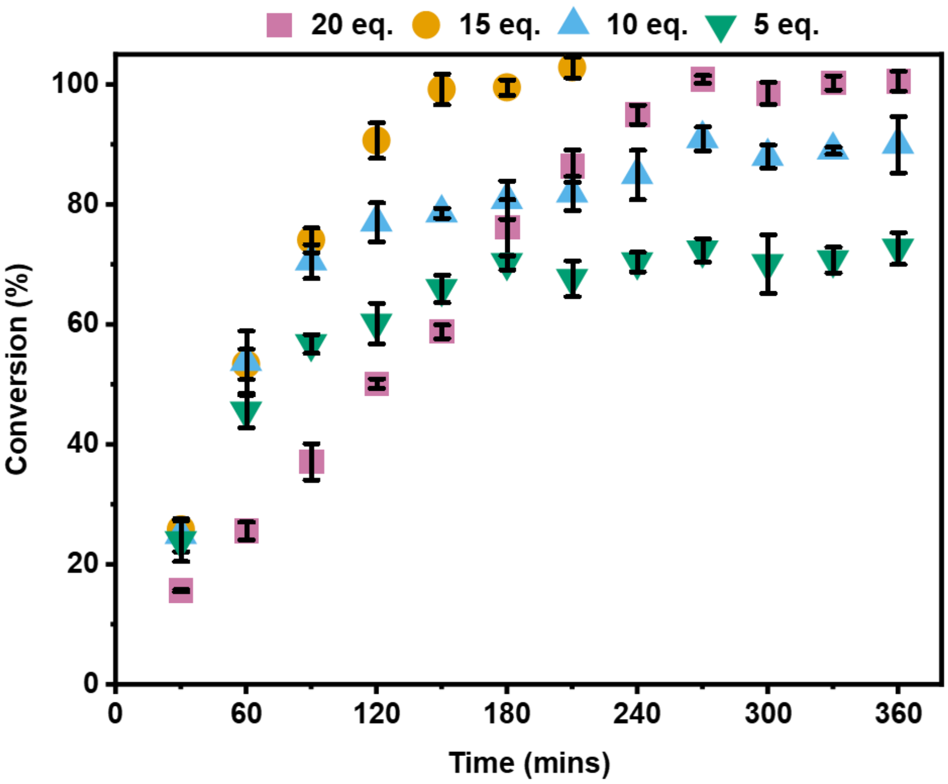

Fig. S28.
Plot of conversion against time for depolymerization reaction varying the equivalents of prenol.

Recovery and Re-use of Excess Prenol from Depolymerization

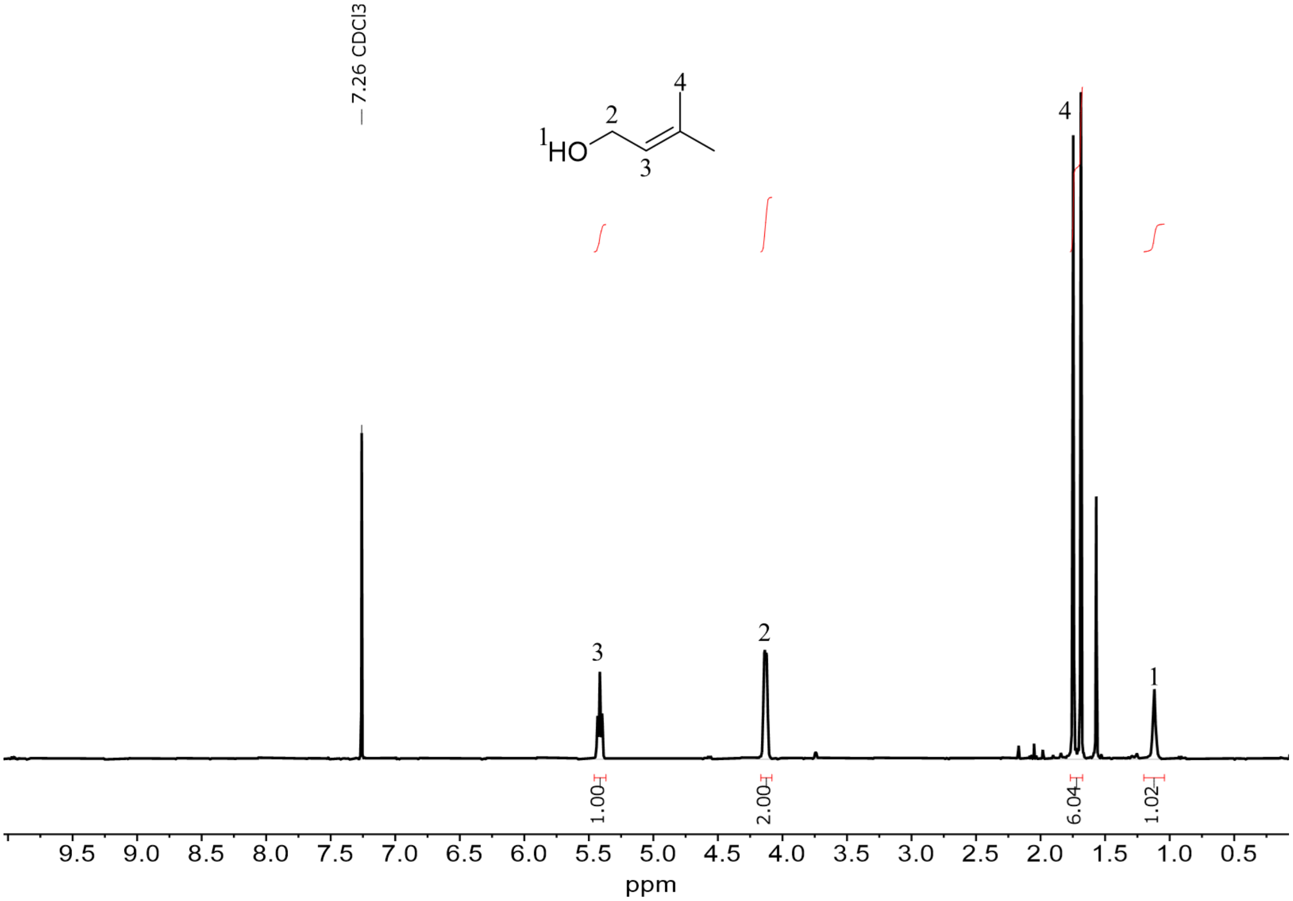

Fig. S29.
^{1}H NMR ($CDCl_3$, 298 K, 400 MHz) spectrum of prenol recovered from the first depolymerization cycle.

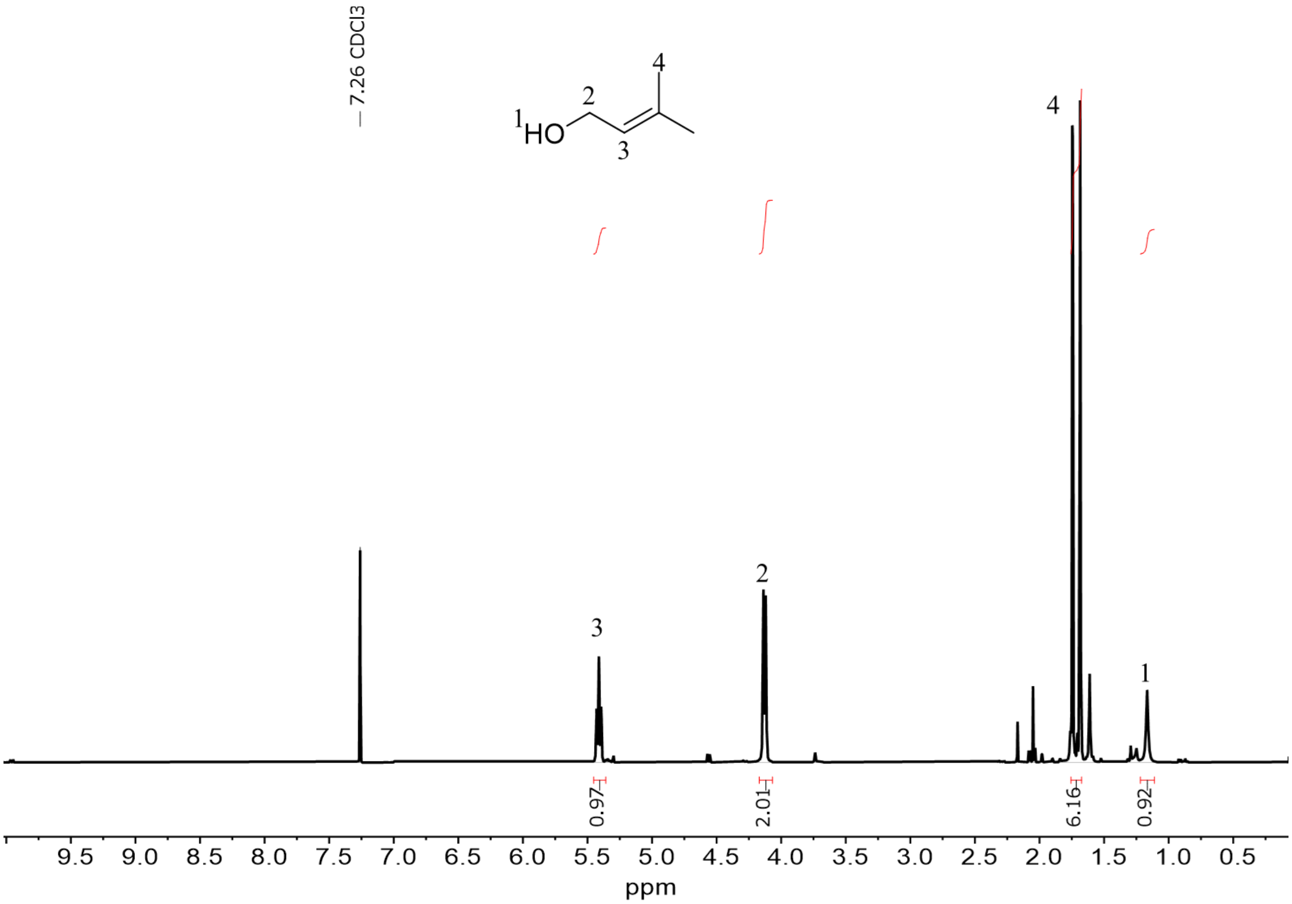

Fig. S30.
^1H NMR ($CDCl_3$, 298 K, 400 MHz) spectrum of prenol recovered from the second depolymerization cycle.

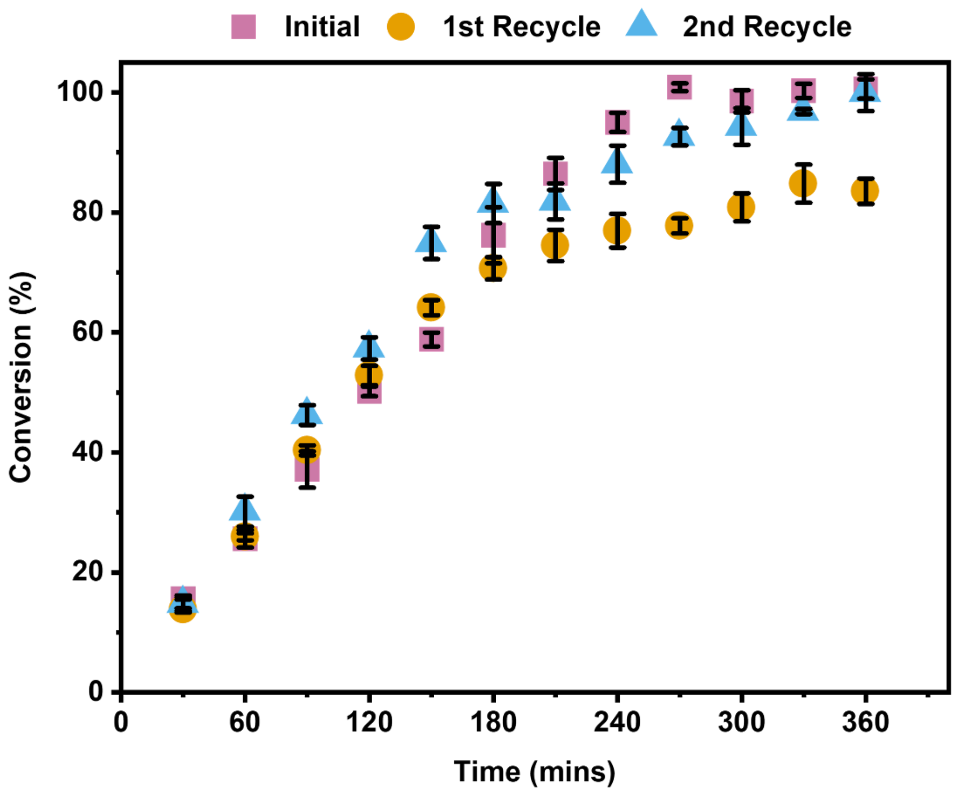

Fig. S31.
Plot of conversion against time for prenolysis of PET using prenol recovered from previous depolymerizations.

Eco Scale Score Calculation for PET Prenolysis

Table S4

Calculation of Eco Scale score using “The Eco Scale” open-access calculator. Eco Scale is calculated as: Eco Scale = 100 - sum of the individual penalties.(*56*) Eco Scale of PET prenolysis = 77.

**Product**	**Value of Penalties**
Prenol	0
PET	0
ZnOAc	0
DMAP	0
Isolated Yield (60%)	-20
Reaction conditions: Heating >1 h	-3
Purification (Cooling to room temperature, Adding solvent, Simple Filtration)	0

Synthesis of PET from BPreT

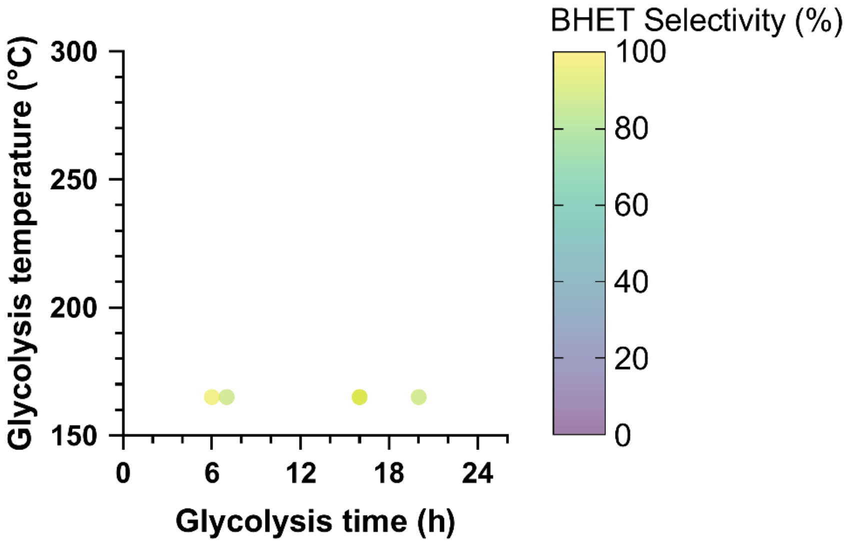

Fig. S32.

Bubble plot of glycolysis time (h) *vs* glycolysis temperature (°C) for the conversion of BPreT into BHET, color coded by BHET selectivity (%).

Table S5.

Synthesis of PET from BPreT *via* BHET under varied conditions. Five runs used a range of catalysts, glycolysis reaction times, polycondensation times, and polycondensation temperatures. The BHET selectivity after glycolysis was measured *via* HPLC, whilst the yield, M_n, DP_n, and color of PET was measured after polycondensation.

**Run**	**1**	**2**	**3**	**4**	**5**	**Commercial PET**
**Catalyst**	$ZnOAc_2$	$ZnOAc_2$	$ZnOAc_2$ / DMAP	TBD / TfOH	TBD / *p*-TSA	-
**Temperature: Stage 1 (°C)**	165					-
**Time: Stage 1 (h)**	16	7	16	16	20	-
**Temperature: Stage 2 (°C)**	165	250	250	280	250	-
**Time: Stage 2 (h)**	4	5.5	24	5.5	5	-
**BHET Selectivity (%)[a] (Stage 1)**	95	87	93	97	88	-
**Yield PET (%)**	37	45	22	55	65	-
**PET M_w (kDa)[b]**	7	174	55	44	42	40
**PET DP_n**	74	1814	576	454	438	428
**PET Colour**	White	Off-White	Off-White	Brown	Brown	White

[a]Detemined by HPLC. [b]Determined by ^1H NMR spectroscopy.

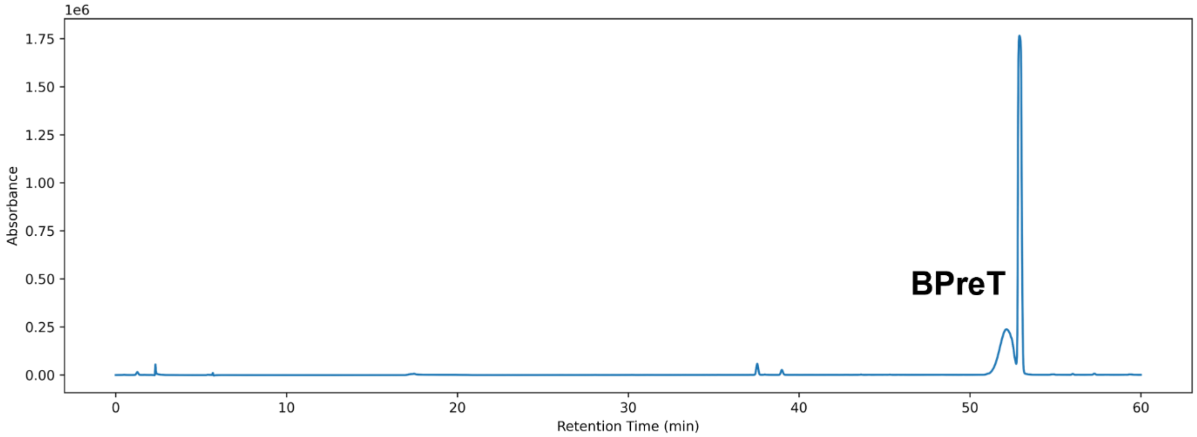

Fig. S33.
HPLC chromatogram of BPreT used for polymerization of PET.

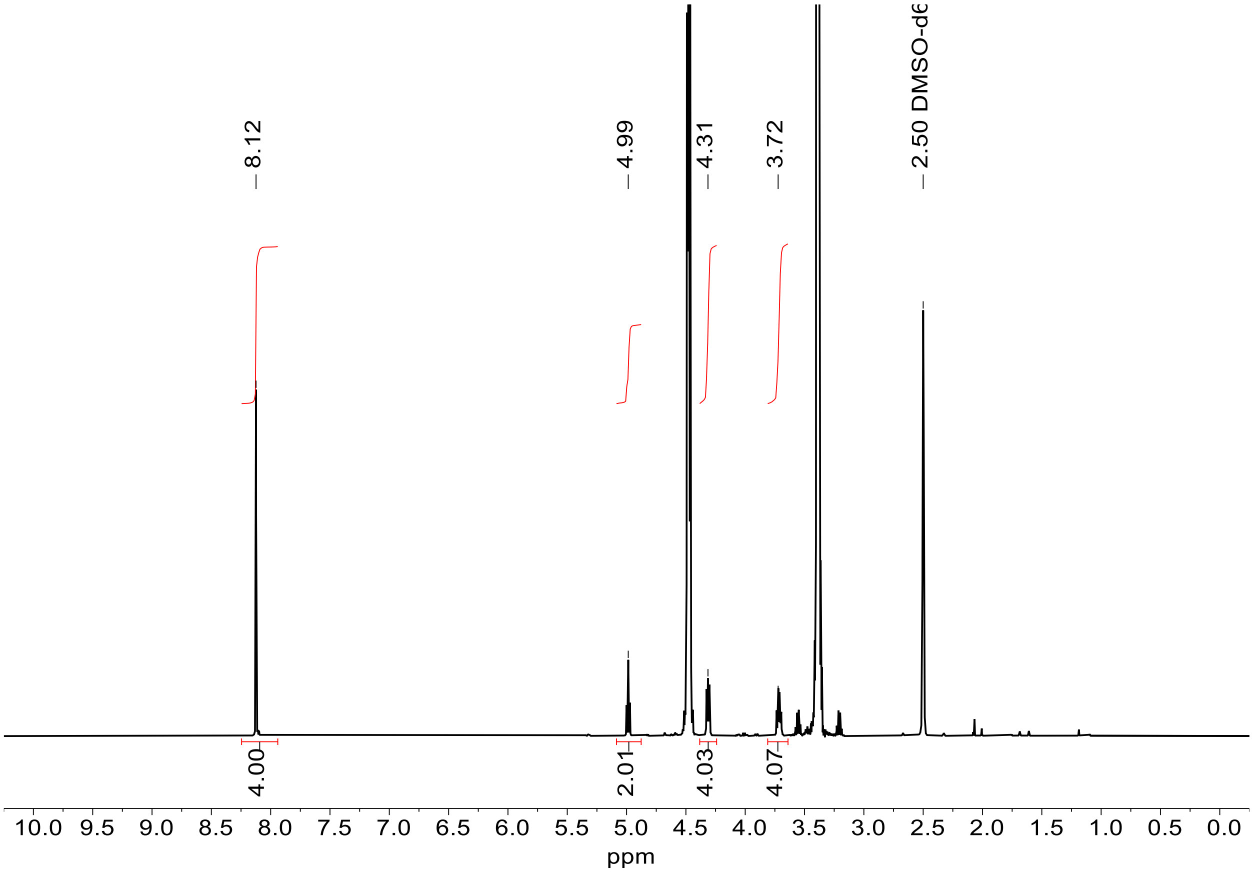

Fig. S34.

^{1}H NMR (DMSO-d_6, 298 K, 400 MHz) spectrum of crude BHET formed in situ prior to the synthesis of PET in run 1, Table S4.

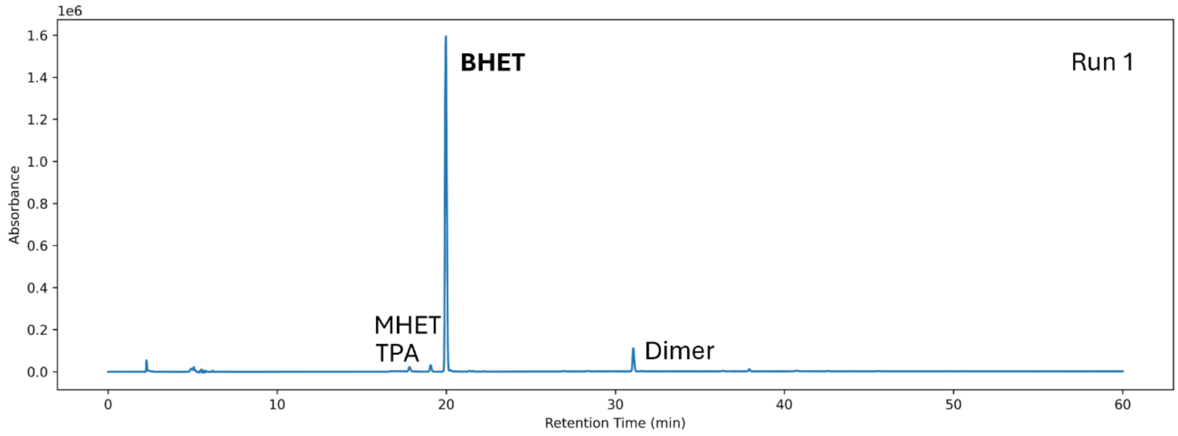

Fig. S35.
HPLC chromatogram for BHET formed in situ prior to the synthesis of PET in run 1, Table S5.

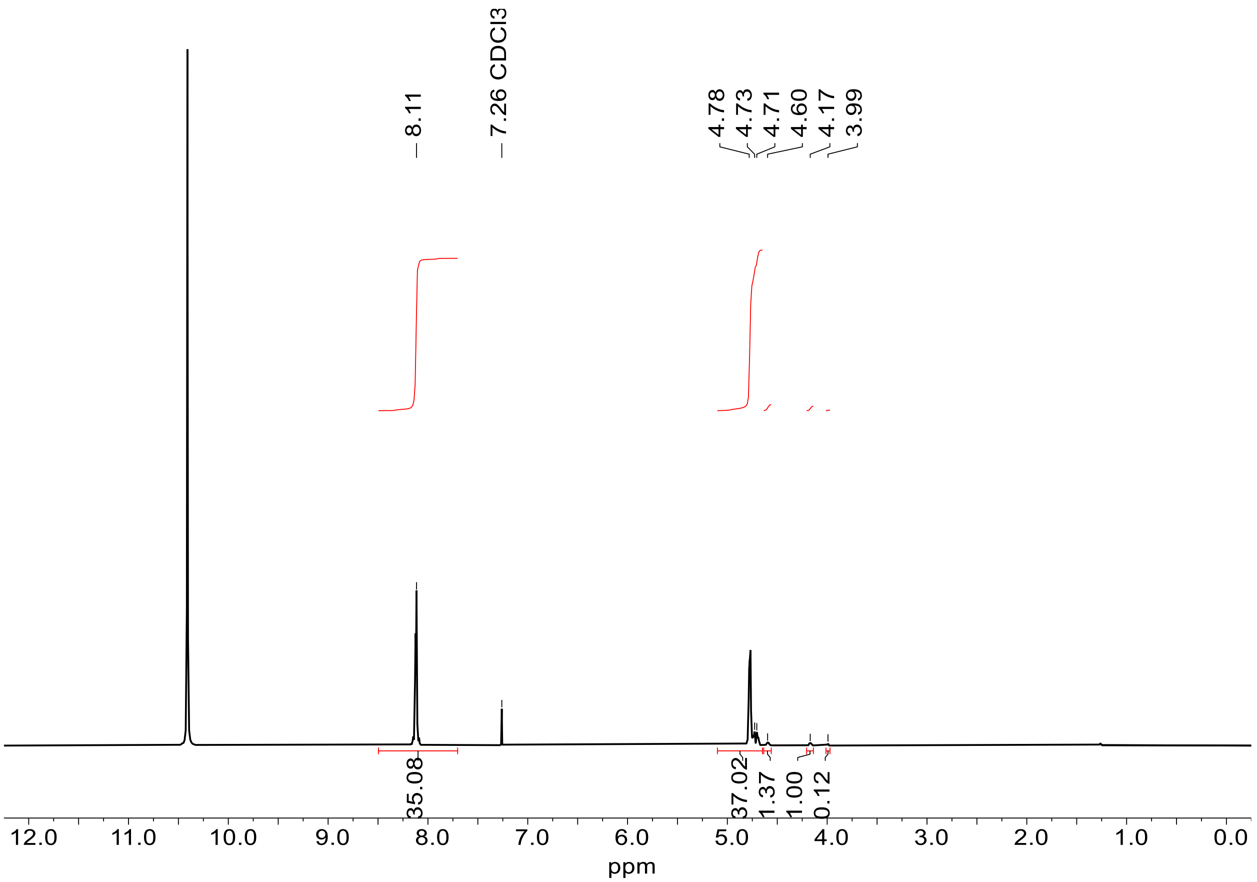

Fig. S36.

^{1}H NMR ($CDCl_3$ / trifluoroacetic acid 8:1 *v/v,* 298 K, 400 MHz) spectrum of PET synthesized in run 1, Table S5.

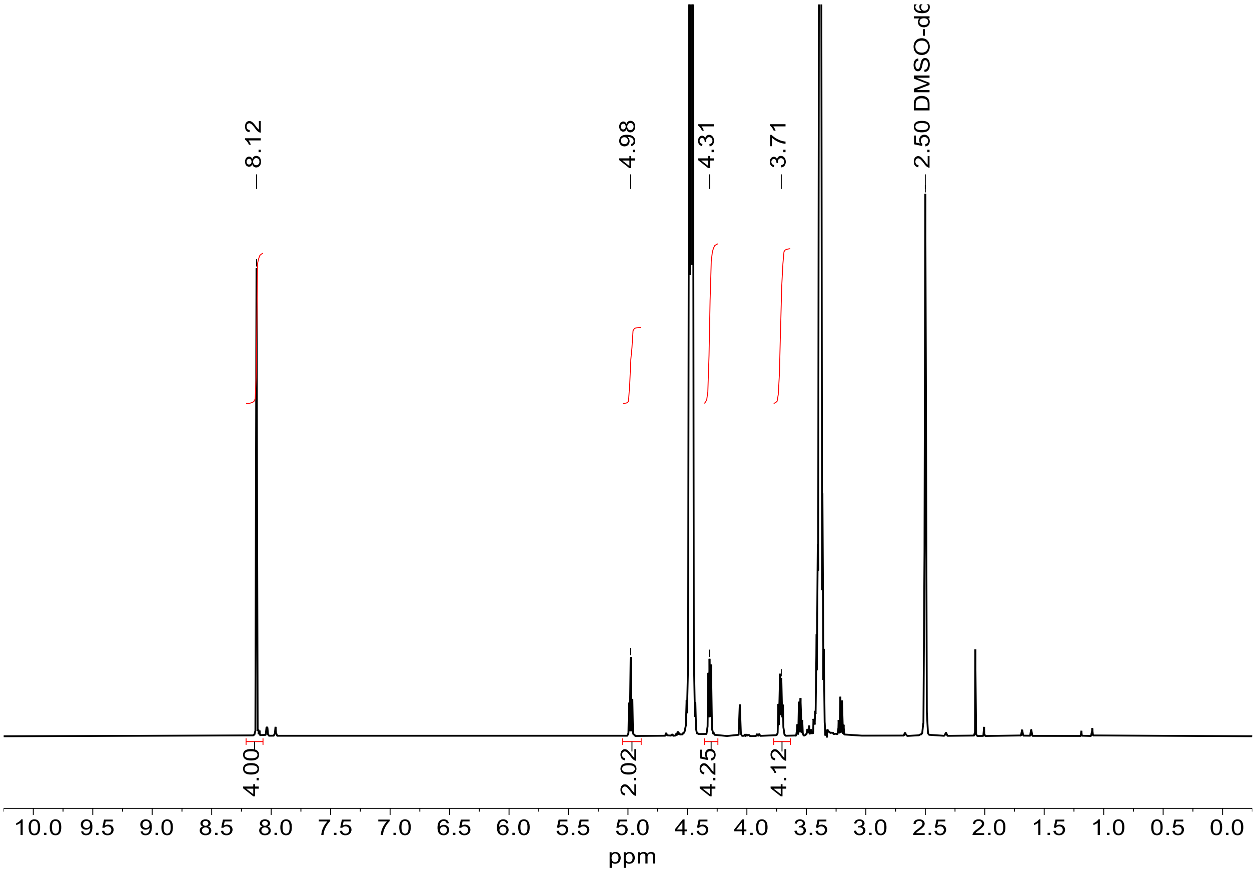

Fig. S37.

^{1}H NMR (DMSO-d_6, 298 K, 400 MHz) spectrum of crude BHET formed in situ prior to the synthesis of PET in run 2, Table S5.

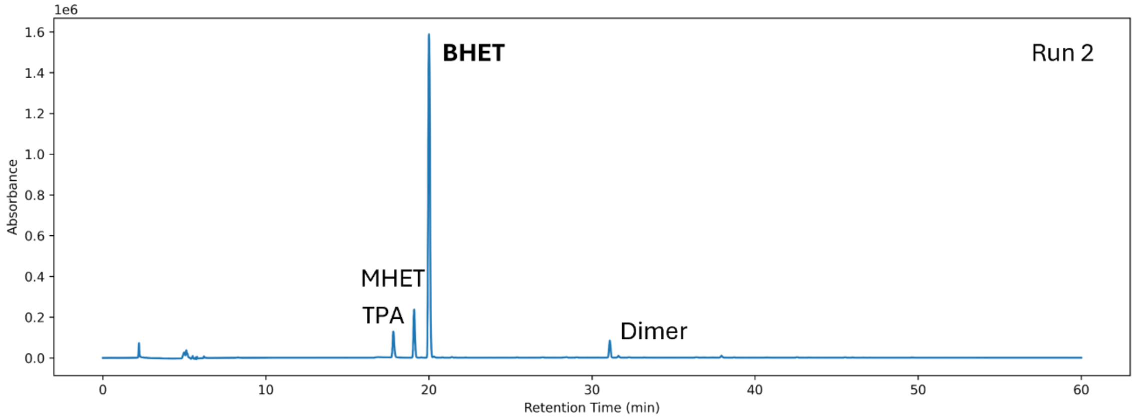

Fig. S38.

HPLC chromatogram for BHET formed in situ prior to the synthesis of PET in run 2, Table S5.

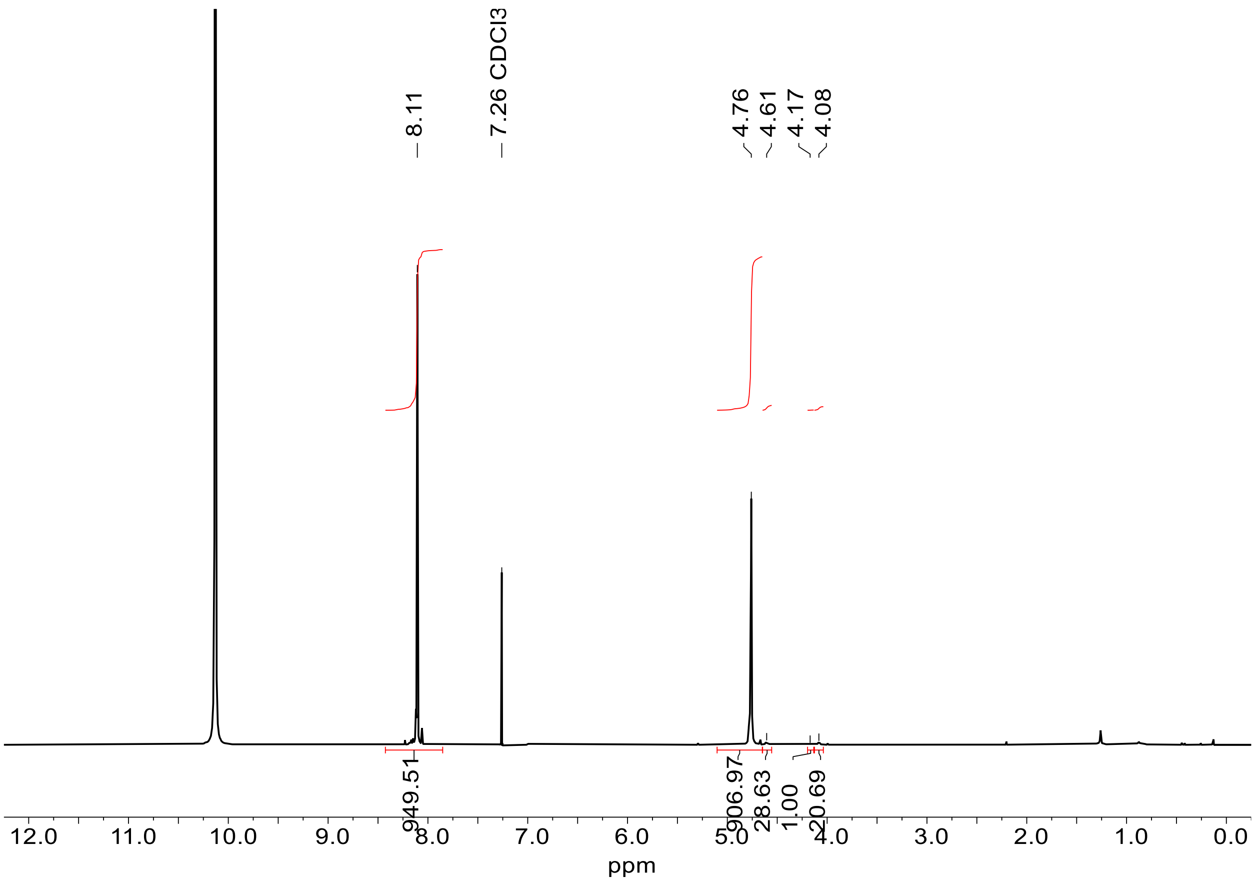

Fig. S39.

^{1}H NMR ($CDCl_3$ / trifluoroacetic acid 8:1 *v/v*, 298 K, 400 MHz) spectrum of PET synthesized in run 2, Table S5.

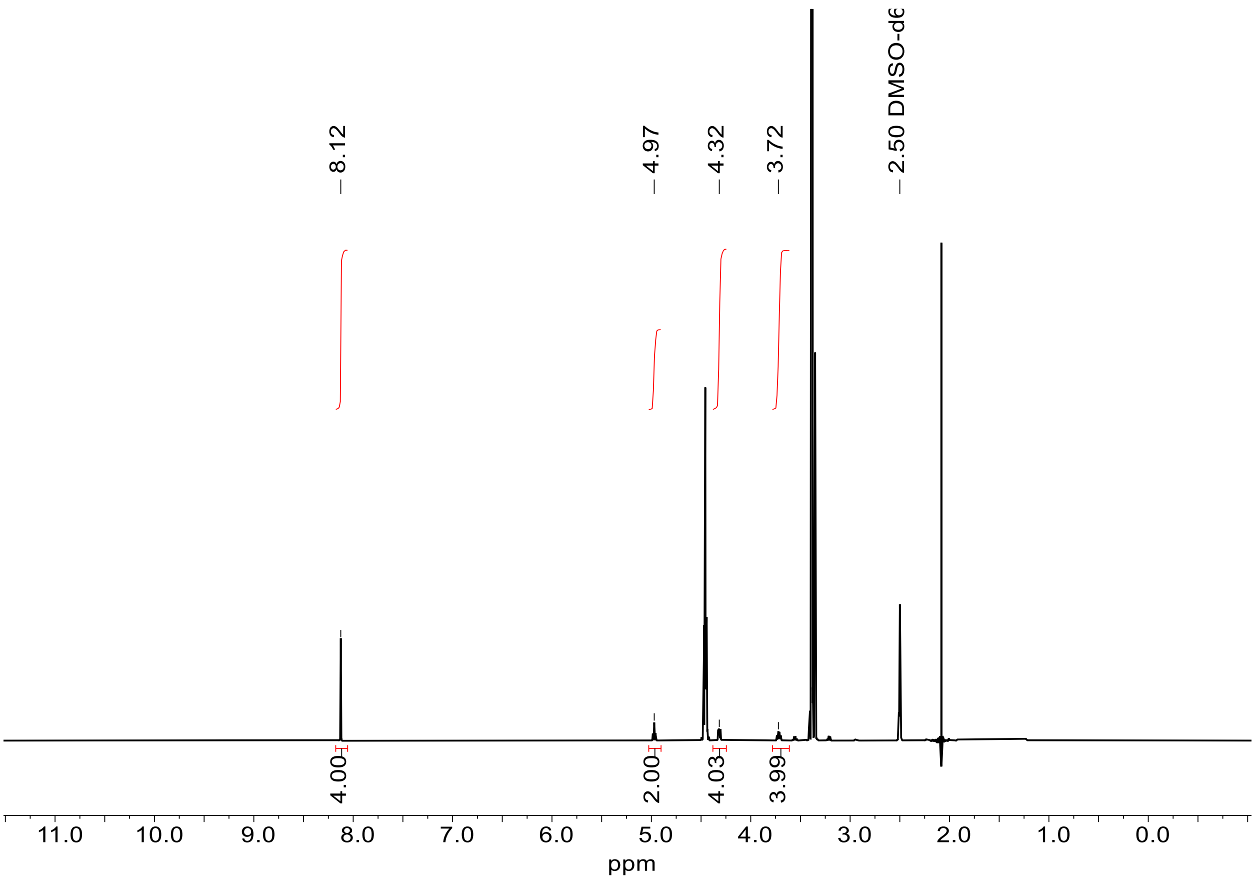

Fig. S40.

^{1}H NMR (DMSO-d_6, 298 K, 400 MHz) spectrum of crude BHET formed in situ prior to the synthesis of PET in run 3, Table S5.

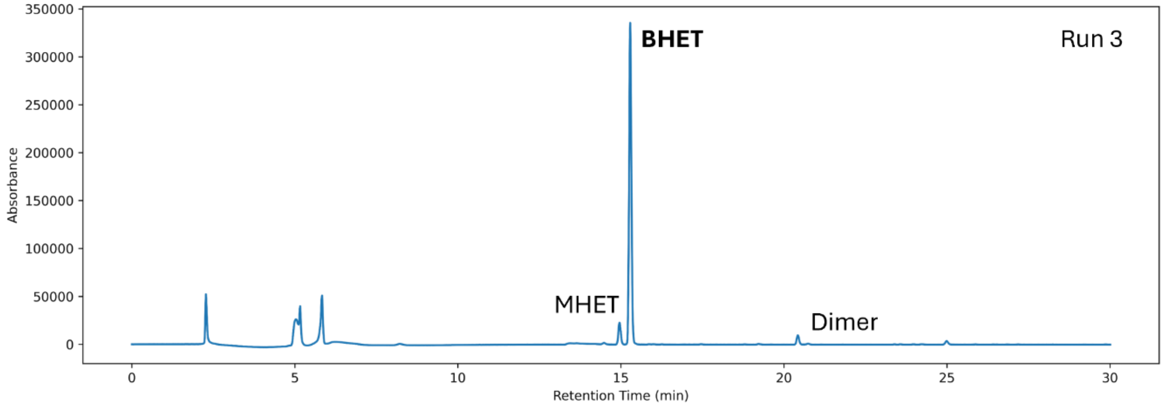

Fig. S41.
HPLC chromatogram for BHET formed in situ prior to the synthesis of PET in run 3, Table S5.

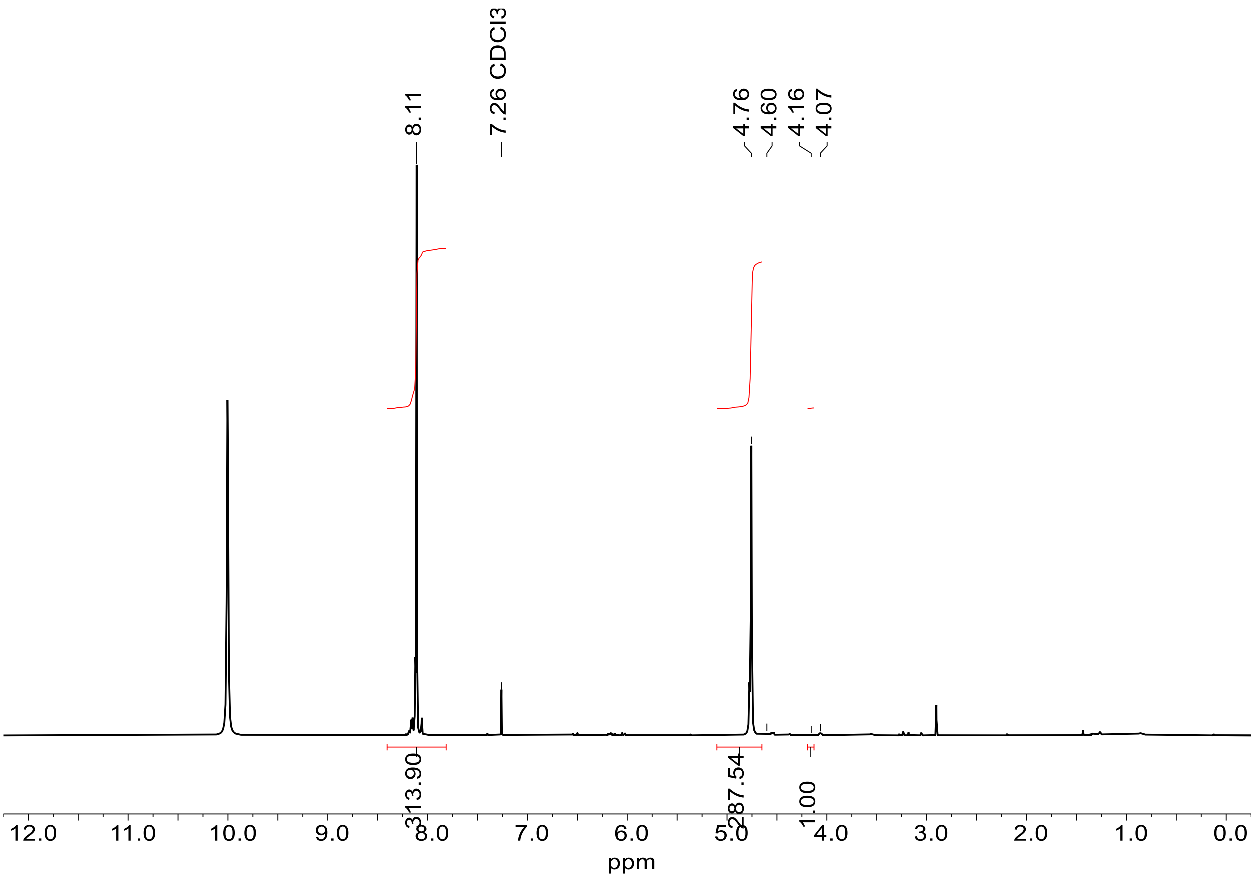

Fig. S42.

^{1}H NMR ($CDCl_3$ / trifluoroacetic acid 8:1 *v/v*, 298 K, 400 MHz) spectrum of PET synthesized in run 3, Table S5.

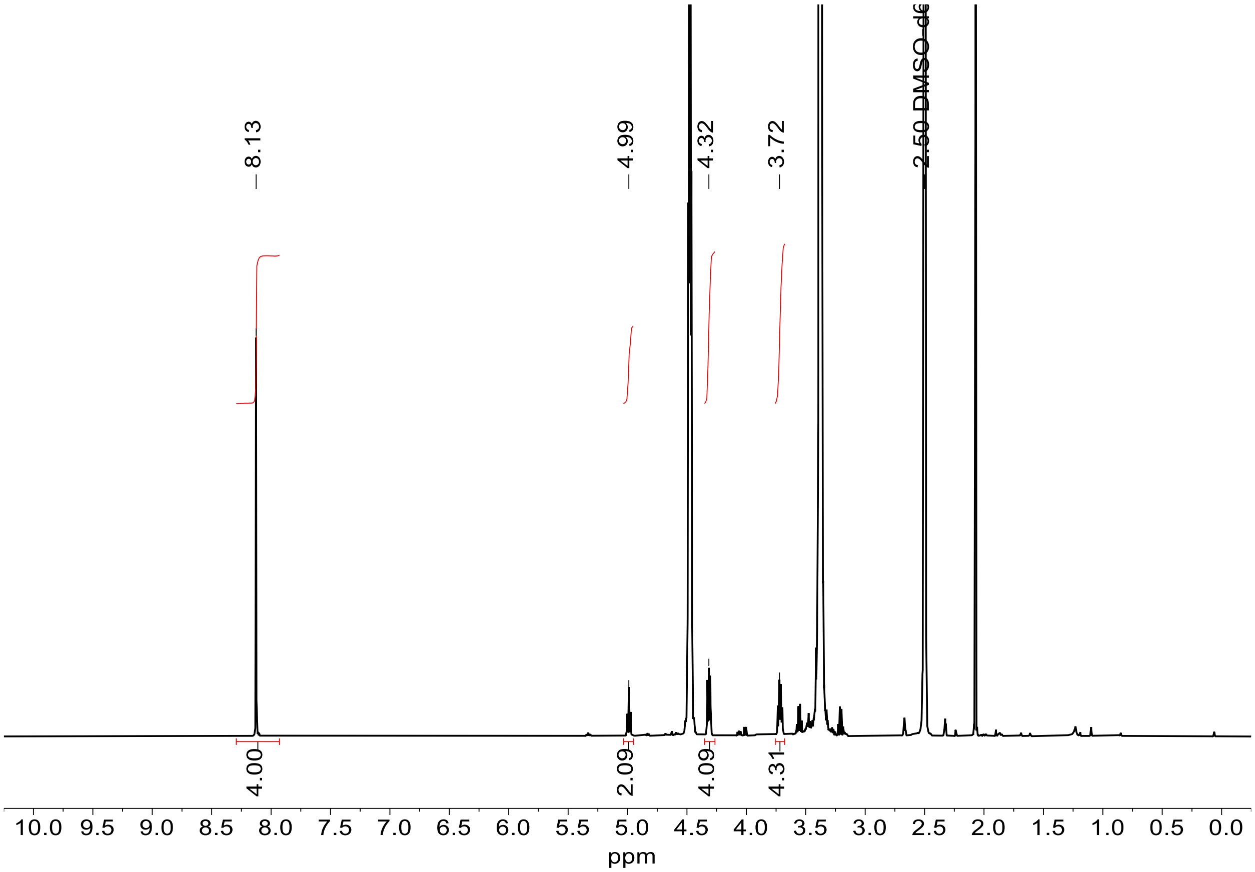

Fig. S43.

^{1}H NMR (DMSO-d_6, 298 K, 400 MHz) spectrum of crude BHET formed in situ prior to the synthesis of PET in run 4, Table S5.

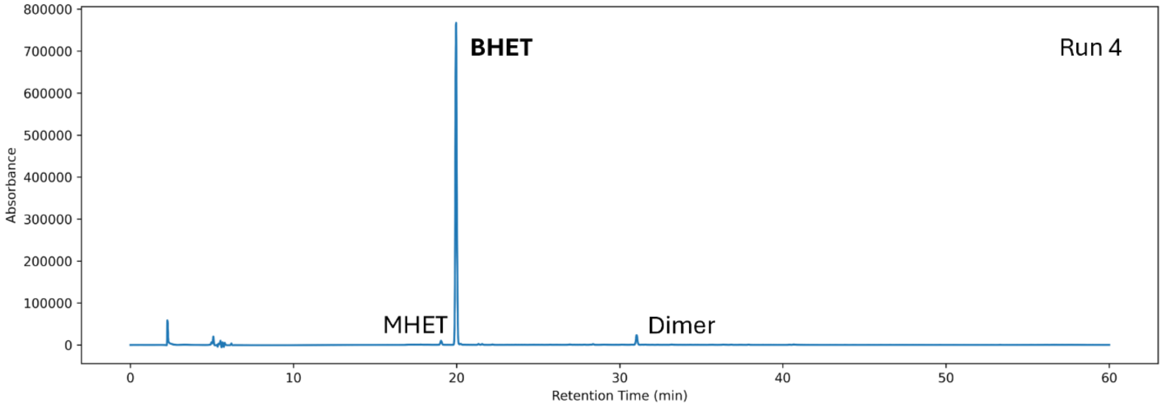

Fig. S44.

HPLC chromatogram for BHET formed in situ prior to the synthesis of PET in run 4, Table S5.

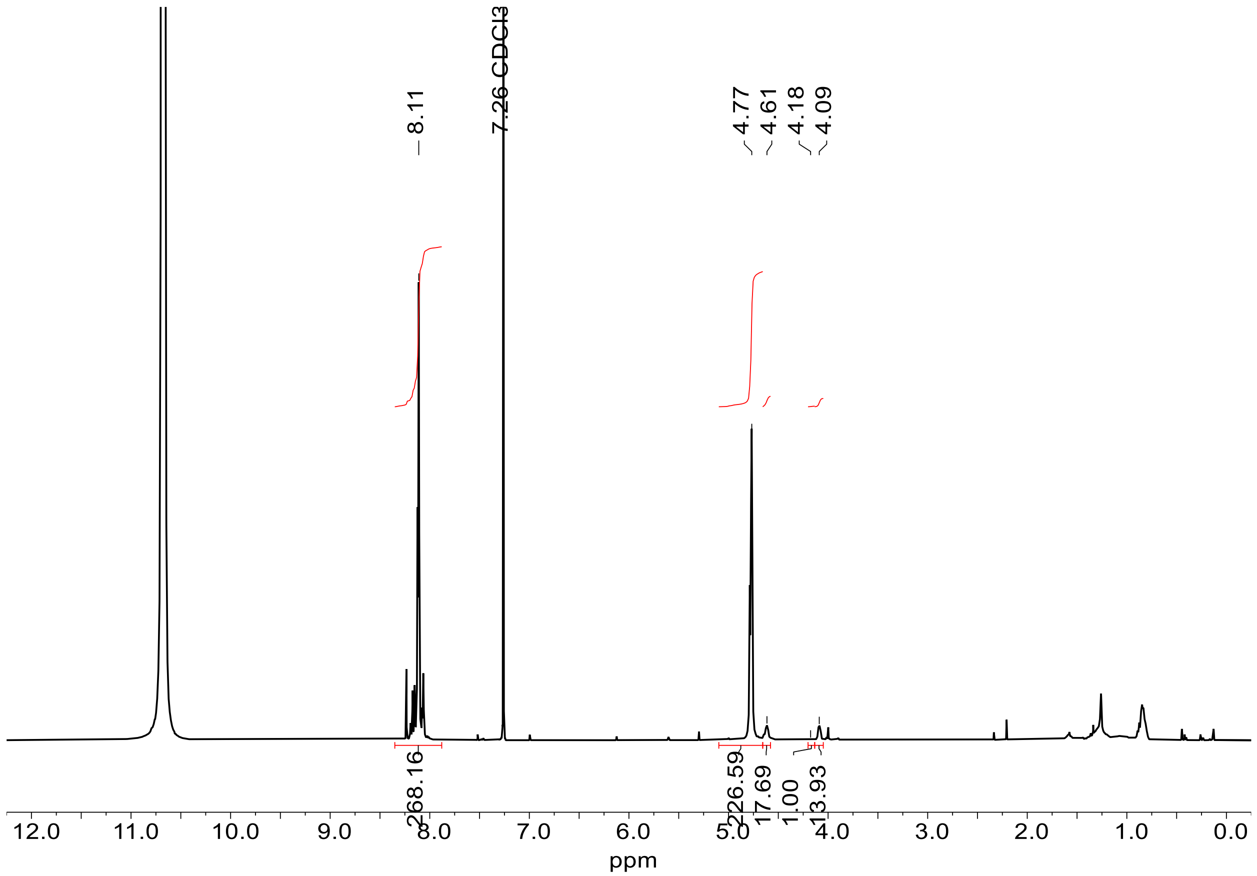

Fig. S45.
^{1}H NMR ($CDCl_3$ / trifluoroacetic acid 8:1 *v/v*, 298 K, 400 MHz) of PET synthesized in run 4, Table S5.

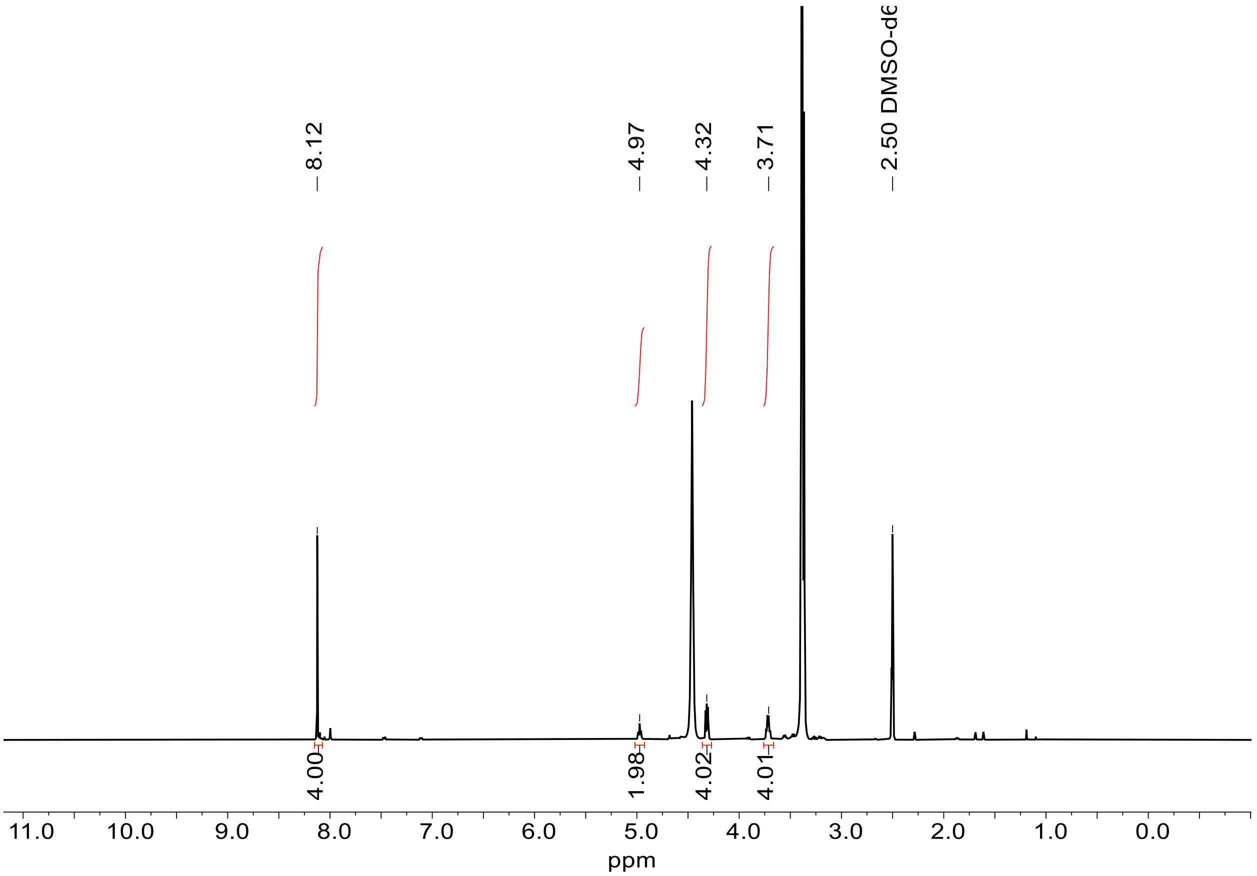

Fig. S46.

^{1}H-NMR (DMSO-d_6, 298 K, 400 MHz) of crude BHET formed in situ prior to the synthesis of PET in run 5, Table S5.

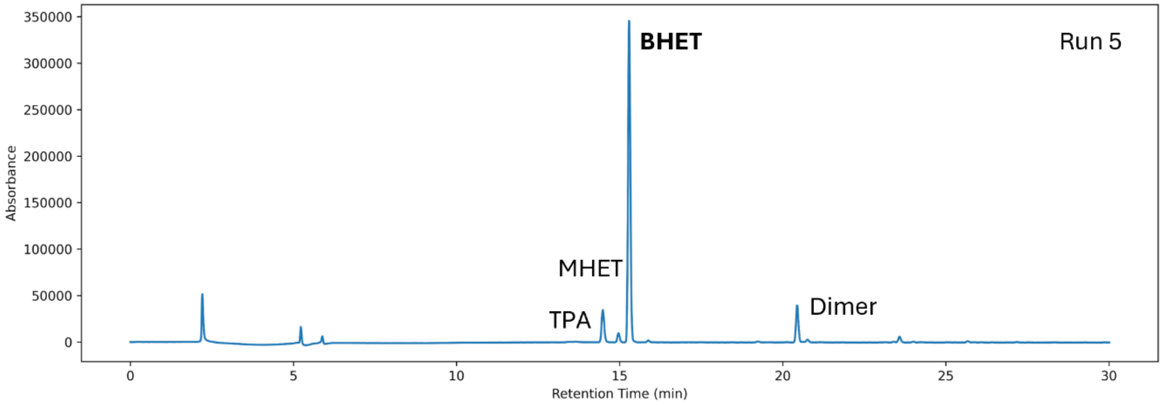

Fig. S47.

HPLC chromatogram for BHET formed in situ prior to the synthesis of PET in run 5, Table S4.

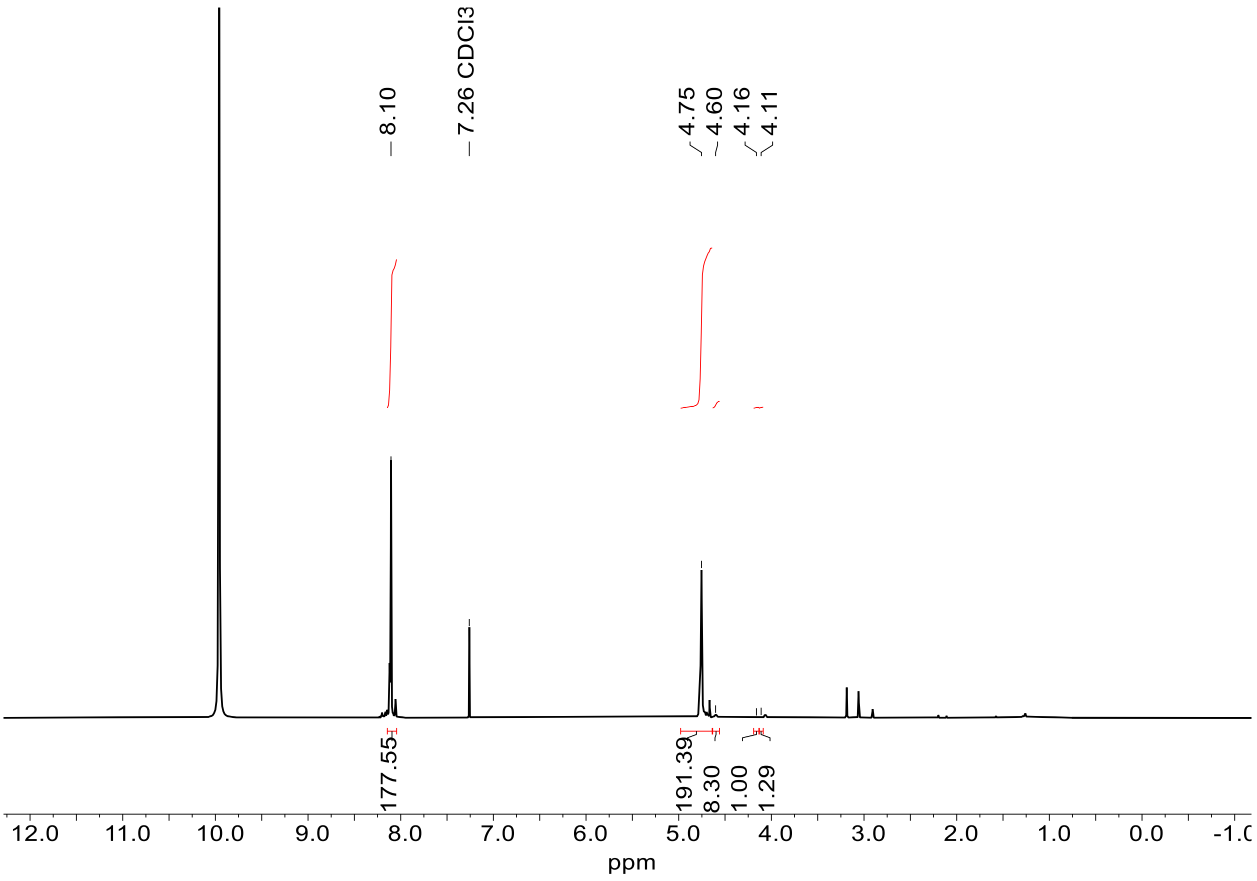

Fig. S48.

^{1}H NMR ($CDCl_3$ / trifluoroacetic acid 8:1 *v/v*, 298 K, 400 MHz) of PET synthesized in run 5, Table S5.

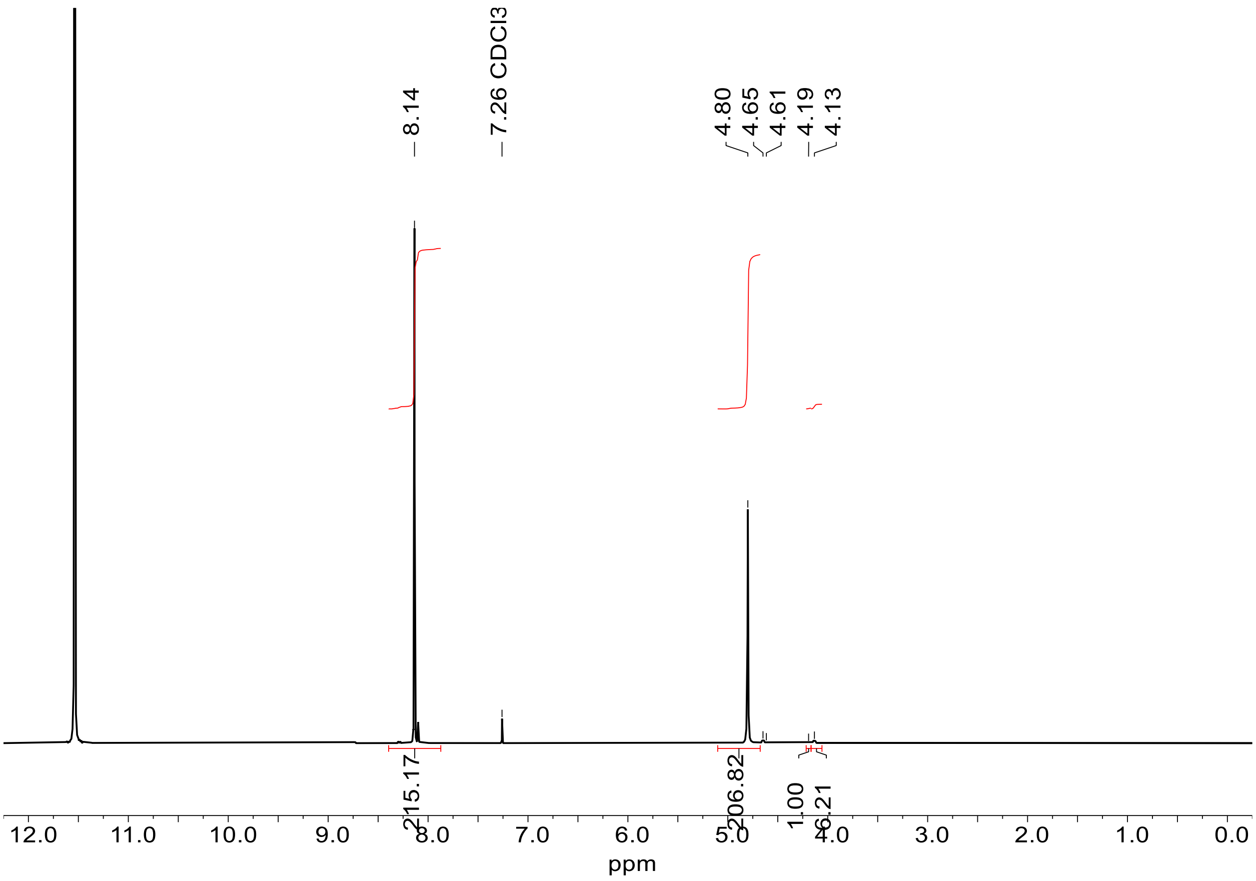

Fig. S49.

^{1}H NMR ($CDCl_3$ / trifluoroacetic acid 8:1 *v/v*, 298 K, 400 MHz) of commercial PET.

Blended Crosslinked Network Composition

Table S6.
Composition of blended crosslinked networks.

**Network ratio**	**Bis(prenyl)terephthalate BPreT**		**Bis(perillyl)terephthalate BPerT**		**TPO-L**	**TMPMP**	**THF**
	mg	mmol	mg	mmol	mg	µL	mL
100:0	302	1.00	0	0.00	19	220	2
75:25	227	0.75	109	0.25	19	220	2
50:50	151	0.50	216	0.50	19	220	2
25:75	76	0.25	325	0.75	18	220	2
0:100	0	0.00	434	1.00	21	220	2

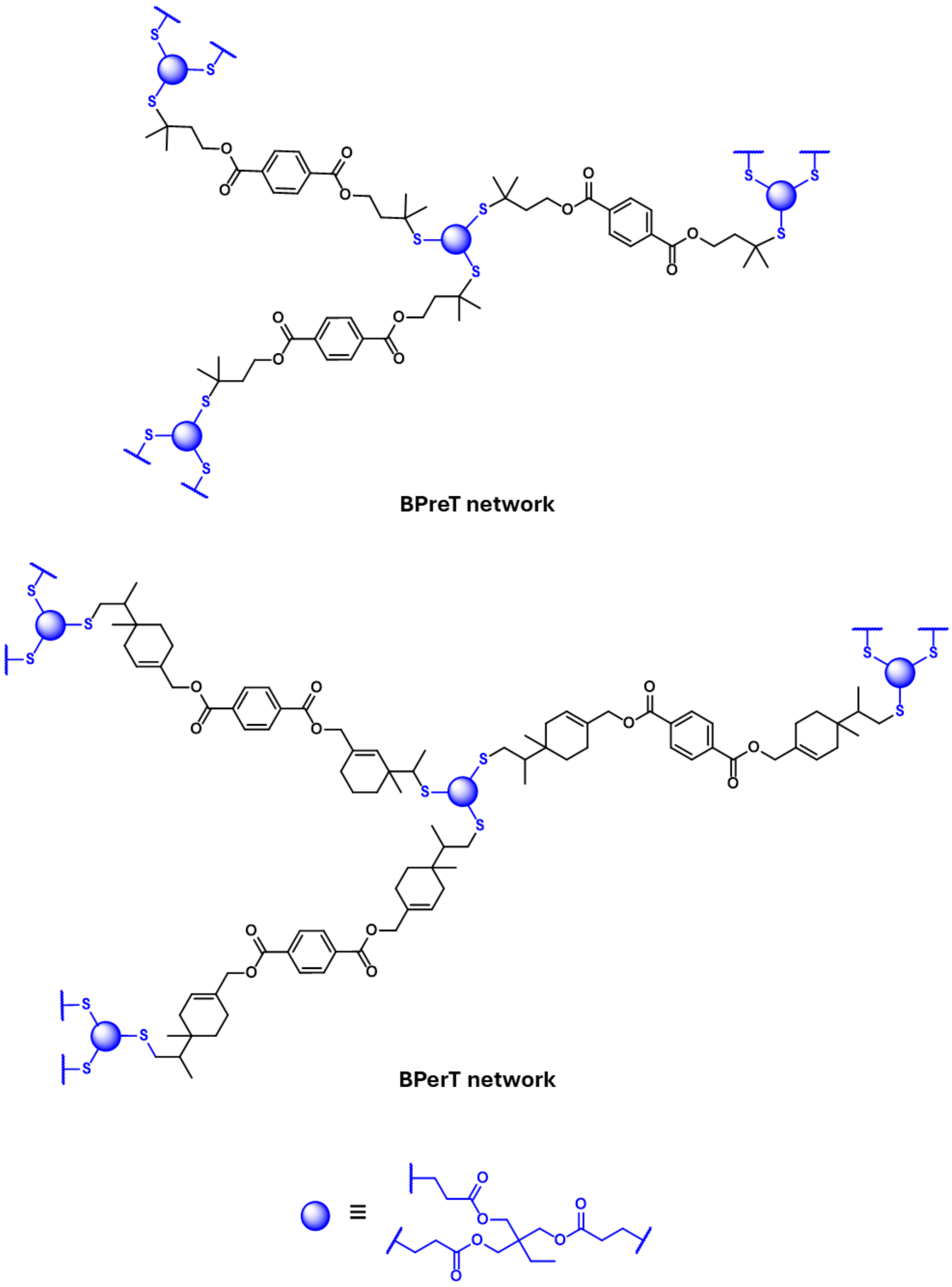

Fig. S50.
Full structure of one repeat unit of BPreT and BPerT networks.

Tensile Testing

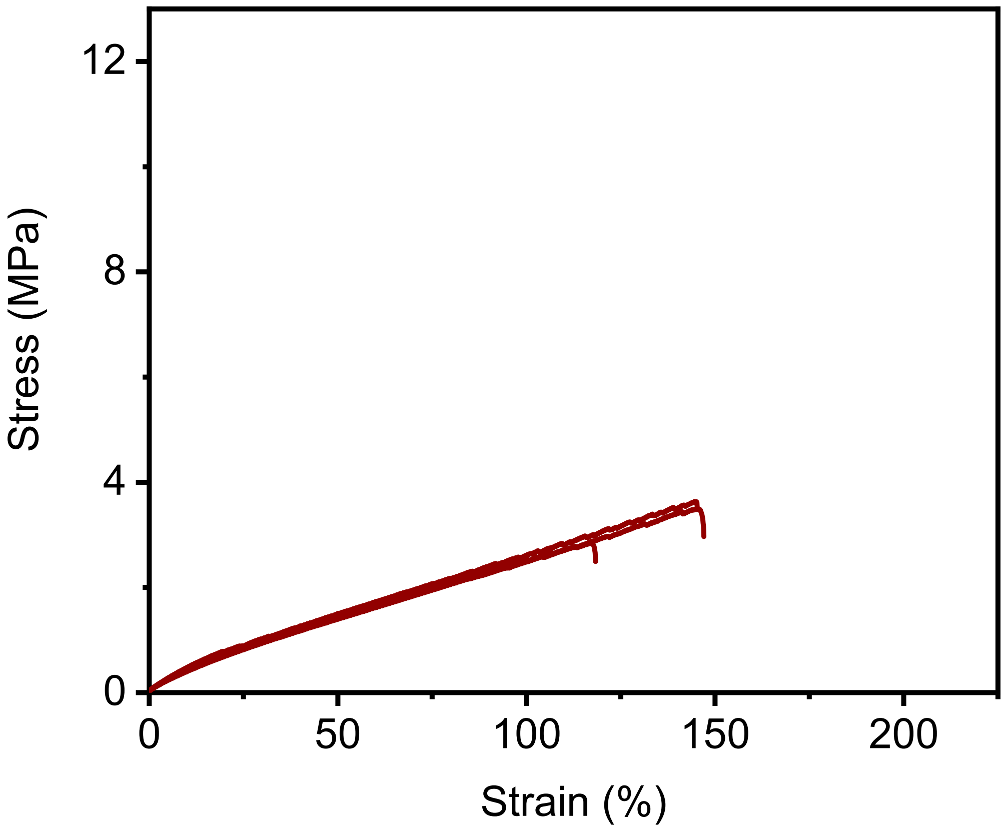

Fig. S51.

Uniaxial tensile testing plot of 100:0 BPreT:BPerT network with three repeats.

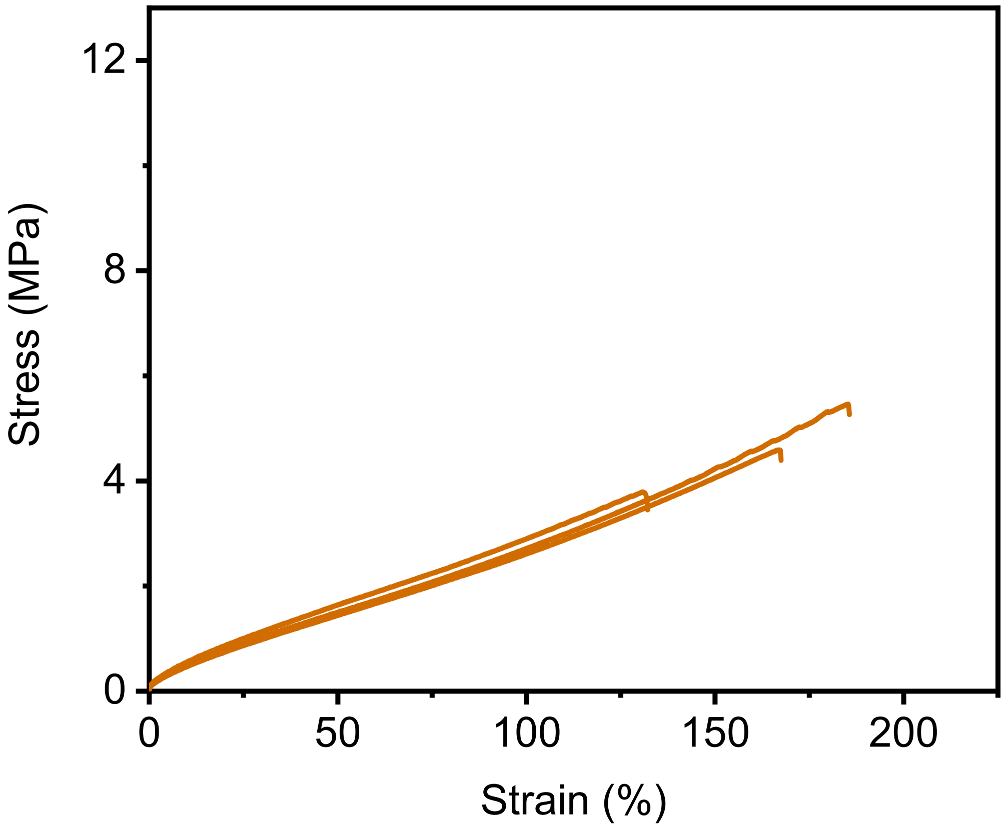

Fig. S52.
Uniaxial tensile testing plot of 75:25 BPreT:BPerT network with three repeats.

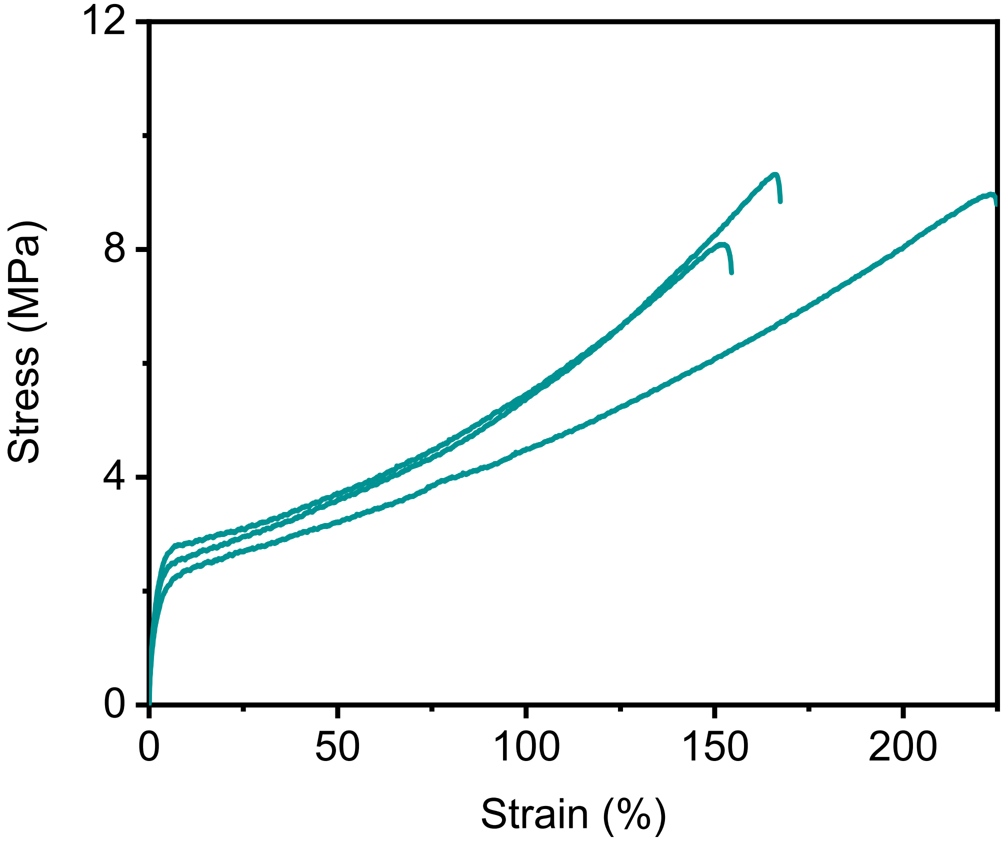

Fig. S53.

Uniaxial tensile testing plot of 50:50 BPreT:BPerT network with three repeats.

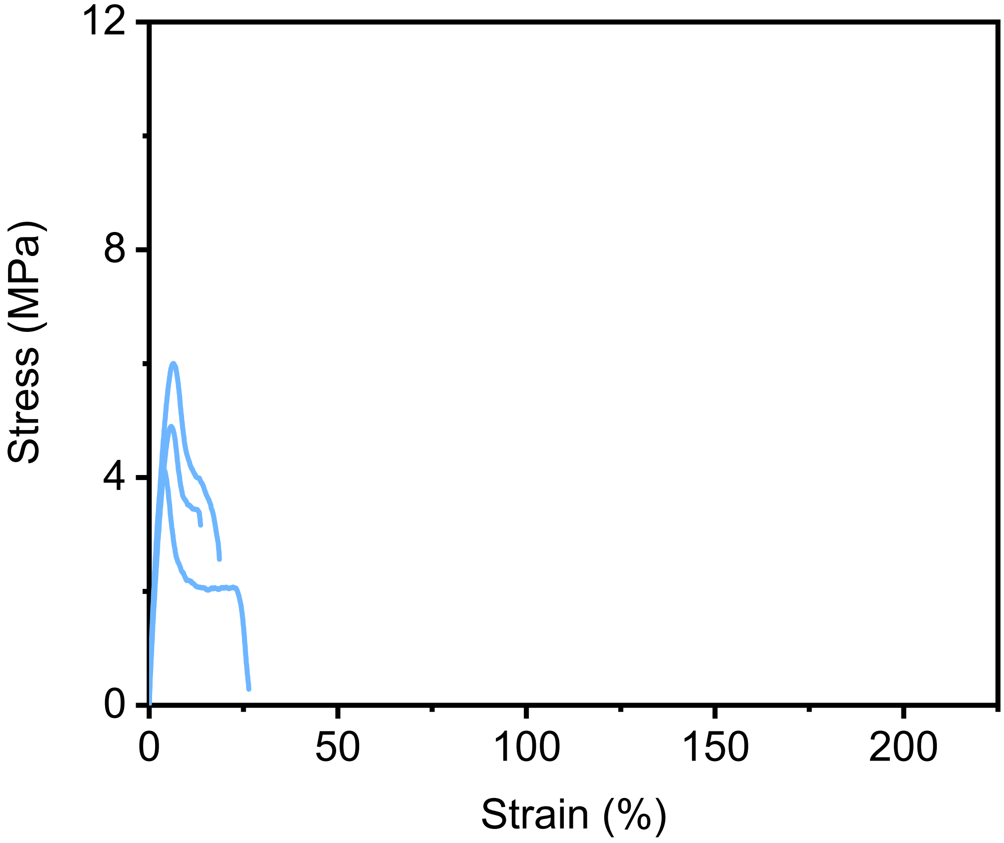

Fig. S54.
Uniaxial tensile testing plot of 25:75 BPreT:BPerT network with three repeats.

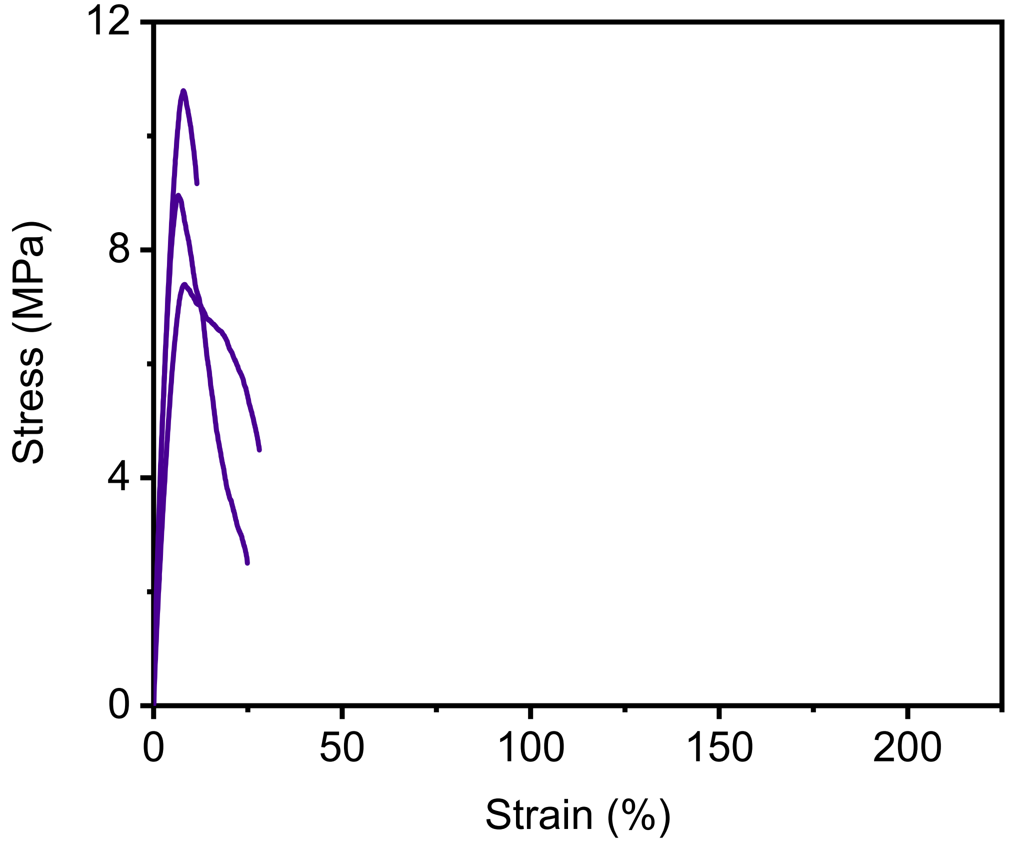

Fig. S55.
Uniaxial tensile testing plot of 0:100 BPreT:BPerT network with three repeats.

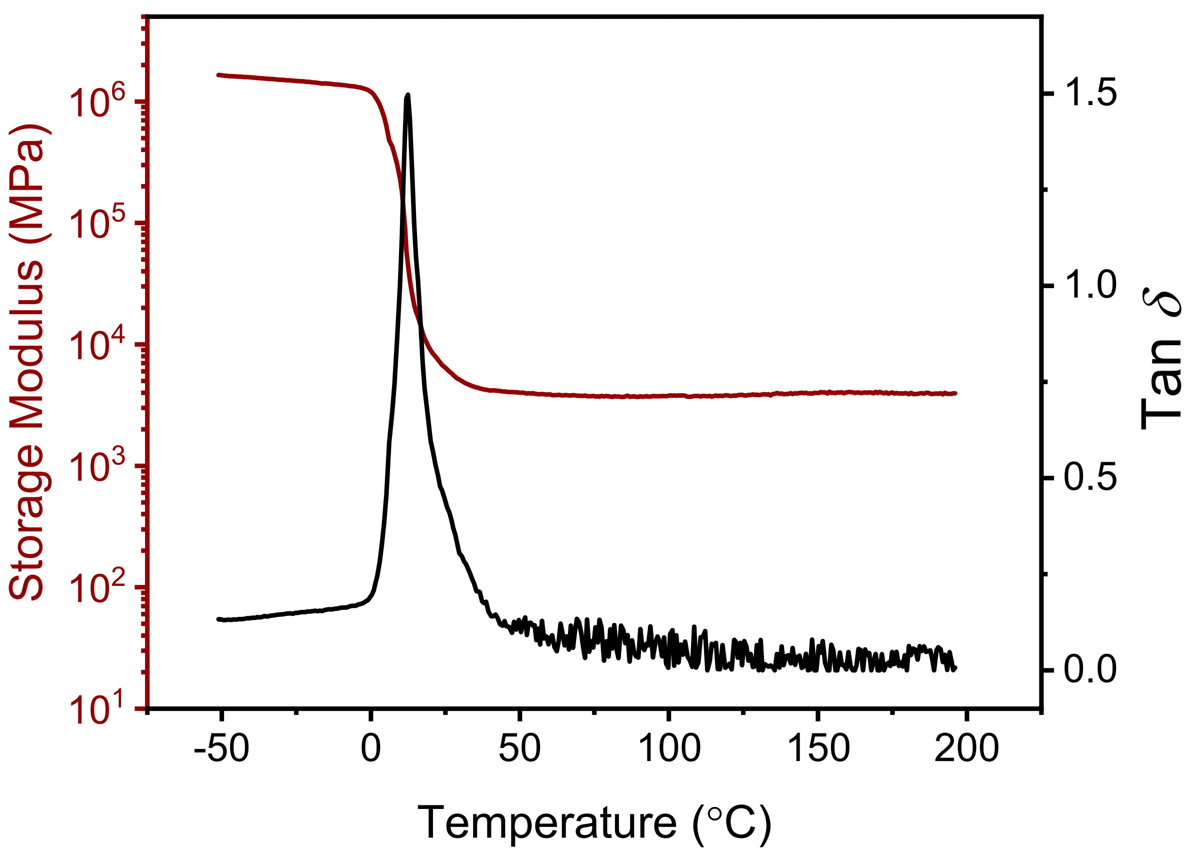

Fig. S56.

DMA temperature sweep of 100:0 BPreT:BPerT network in a temperature range from -50 to 200 °C at 5 °C min^{-1}.

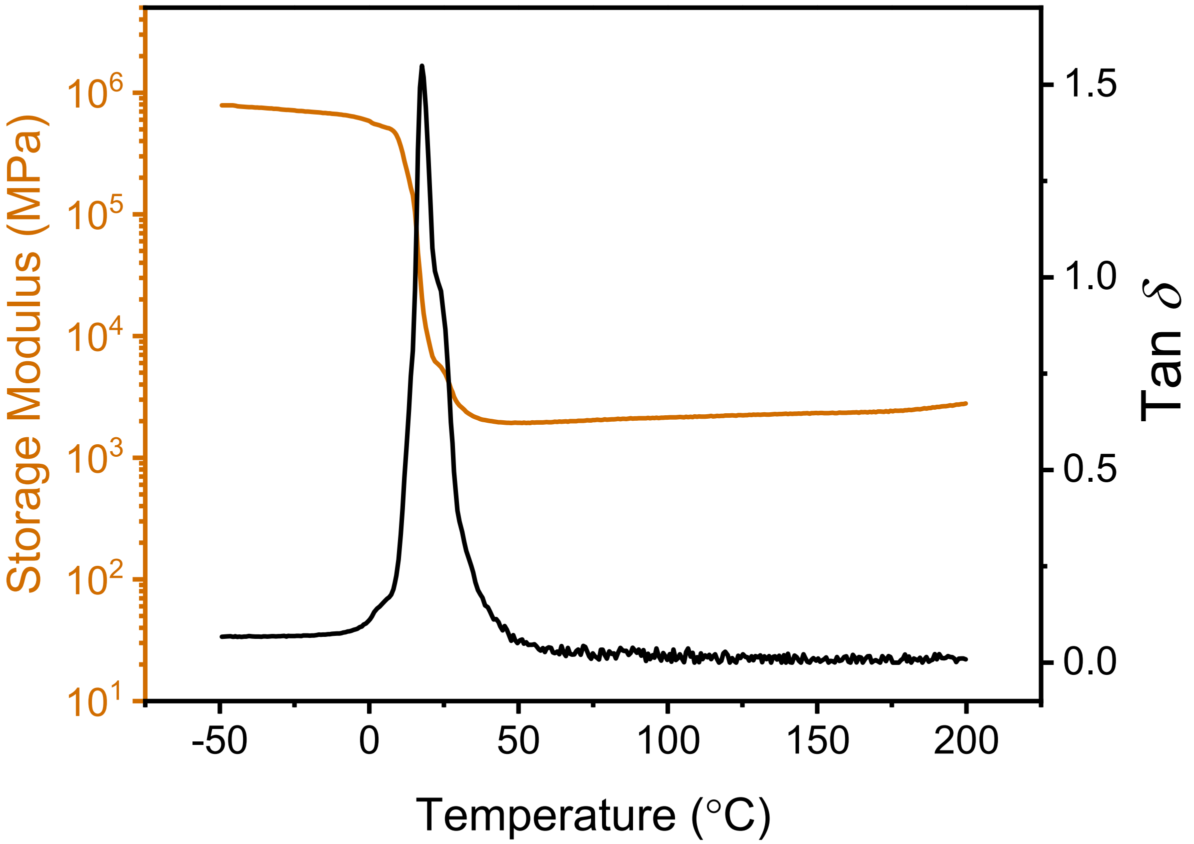

Fig. S57.

DMA temperature sweep of 75:25 BPreT:BPerT network in a temperature range from -50 to 200 °C at 5 °C min^{-1}.

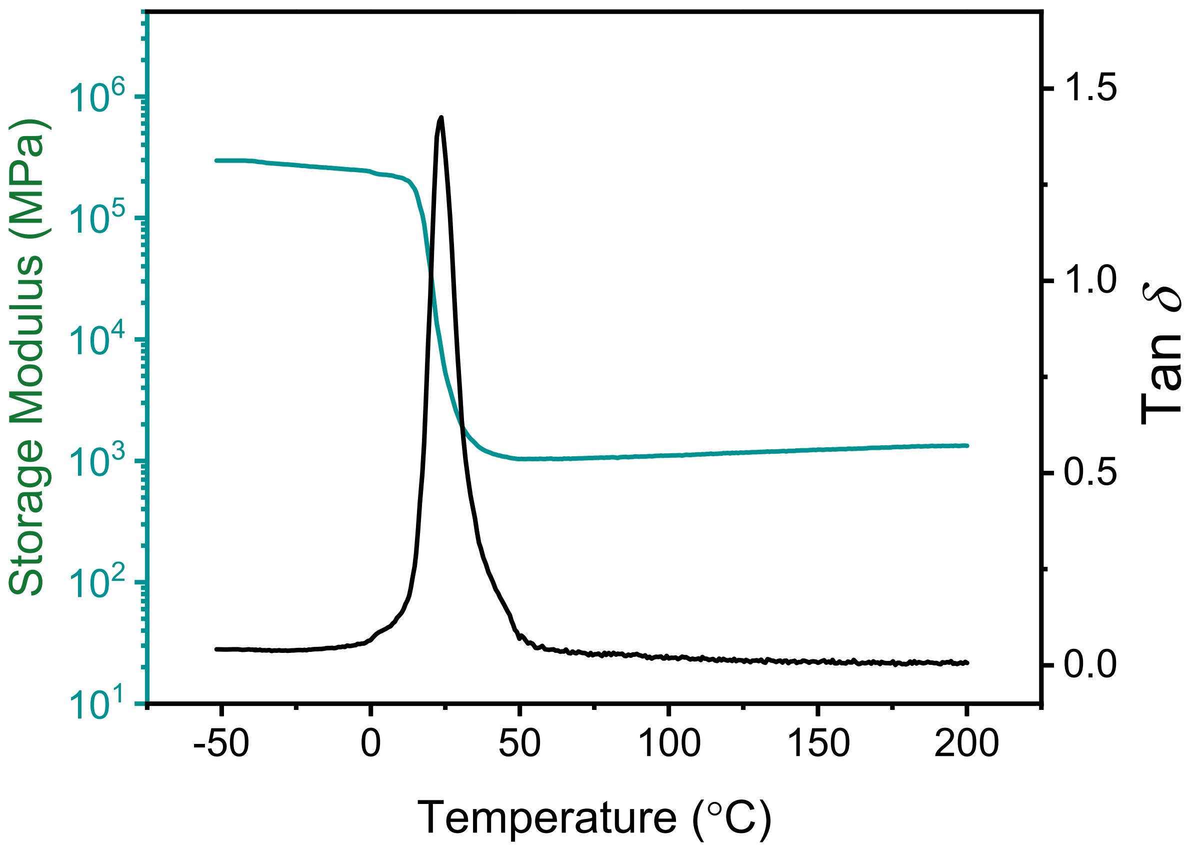

Fig. S58.

DMA temperature sweep of 50:50 BPreT:BPerT network in a temperature range from -50 to 200 °C at 5 °C min^{-1}.

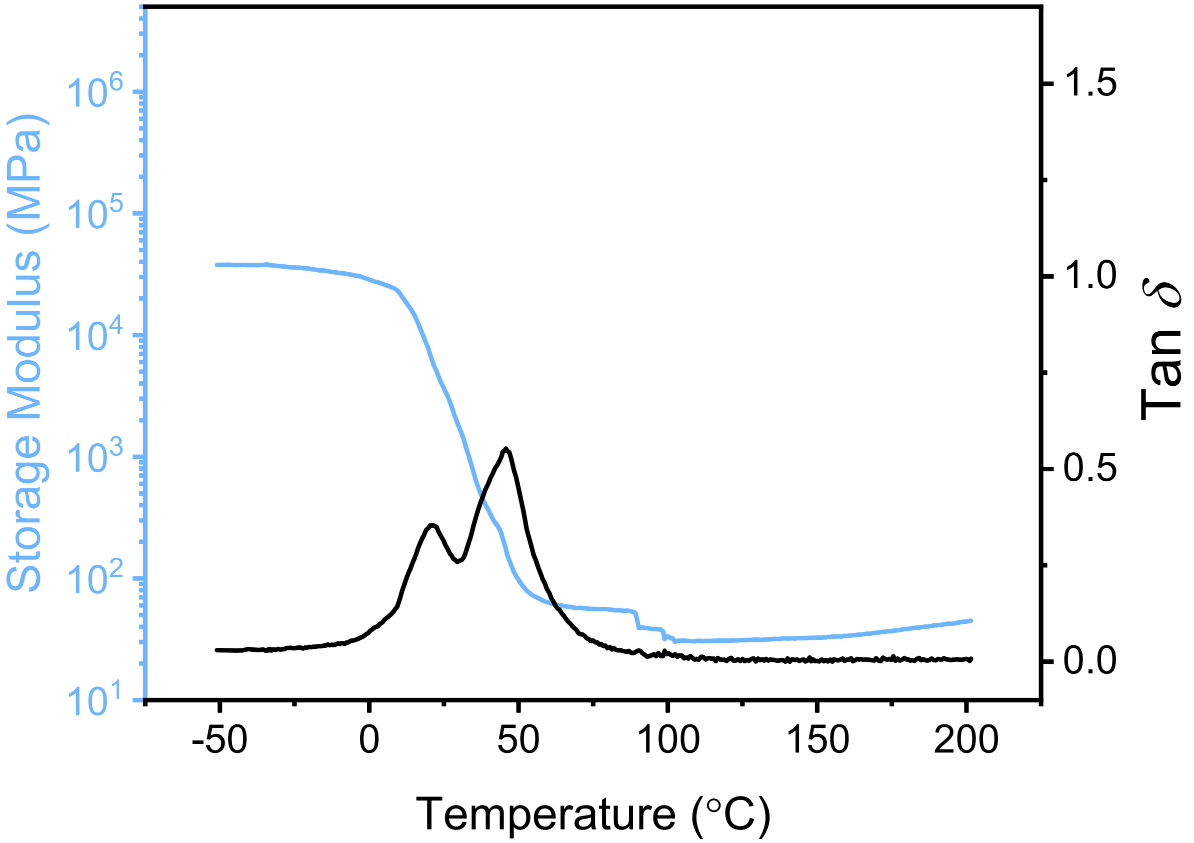

Fig. S59.

DMA temperature sweep of 25:75 BPreT:BPerT network in a temperature range from -50 to 200 °C at 5 °C min^{-1}.

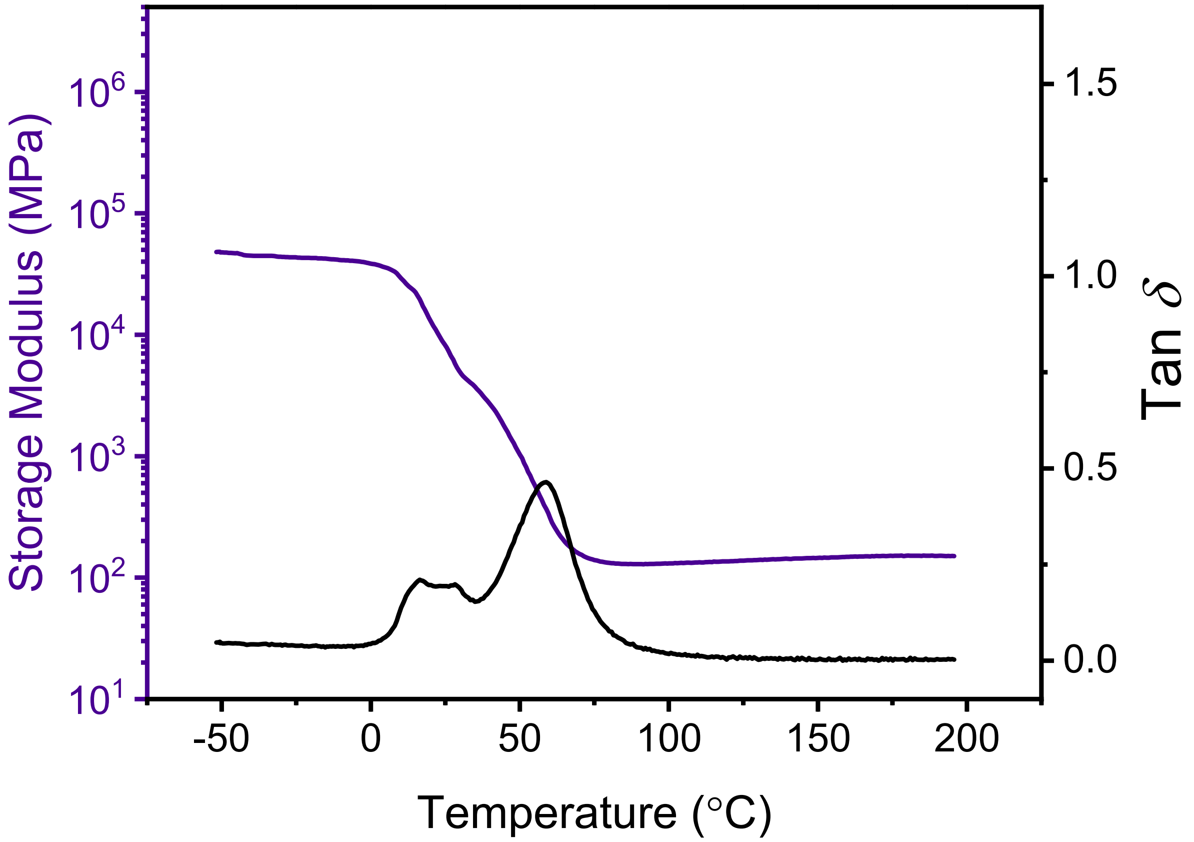

Fig. S60.

DMA temperature sweep of 0:100 BPreT:BPerT network in a temperature range from -50 to 200 °C at 5 °C min^{-1}.

3D Printing Photocurable Resin Formulation

Table S7.
Summary of photocurable resin components.

**Resin component**	**Loading (wt%)**	**Function**
Bis(prenyl)terephthalate	37.50	Monomer
Trimethylolpropane tris(3-mercaptopropionate) (TMPMP)	32.96	Monomer
Phenylbis(2,4,6-trimethylbenzoyl) phosphine oxide (BAPO)	2.08	Photoinitiator
Sudan II	0.03	Opaquing agent
N-Methyl-2-pyrrolidone (NMP)	27.44	Diluent

.

Rheology

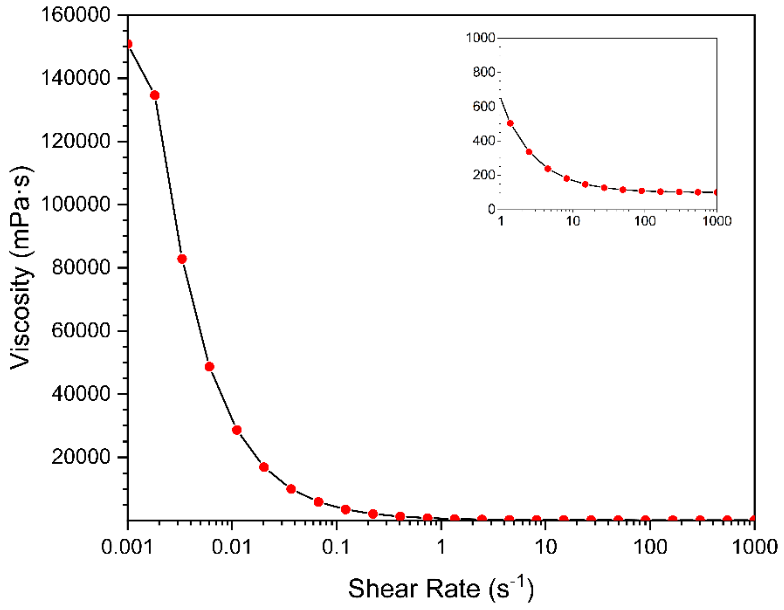

Fig. S61.

Shear rate sweep of 3D printing resin. Demonstrating a viscosity in the linear region of ca. 100 mPa·s.

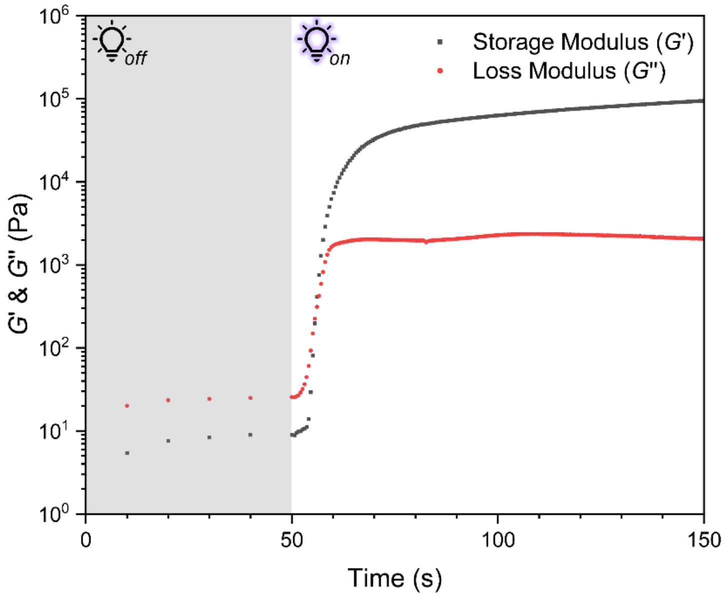

Fig. S62.

Evolution of storage and loss moduli during photocuring. Intersection of the moduli demonstrates gelation after 6 s.

Network Depolymerization

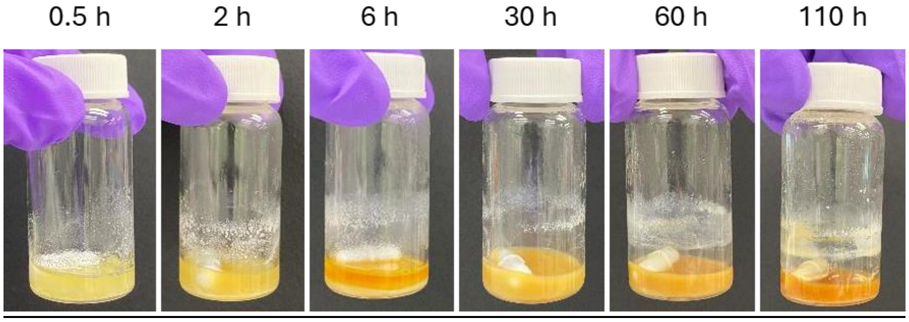

Fig. S63.
Photographs of the reaction mixture during the depolymerization of BPreT network.

Repolymerized Network Thermomechanical Properties

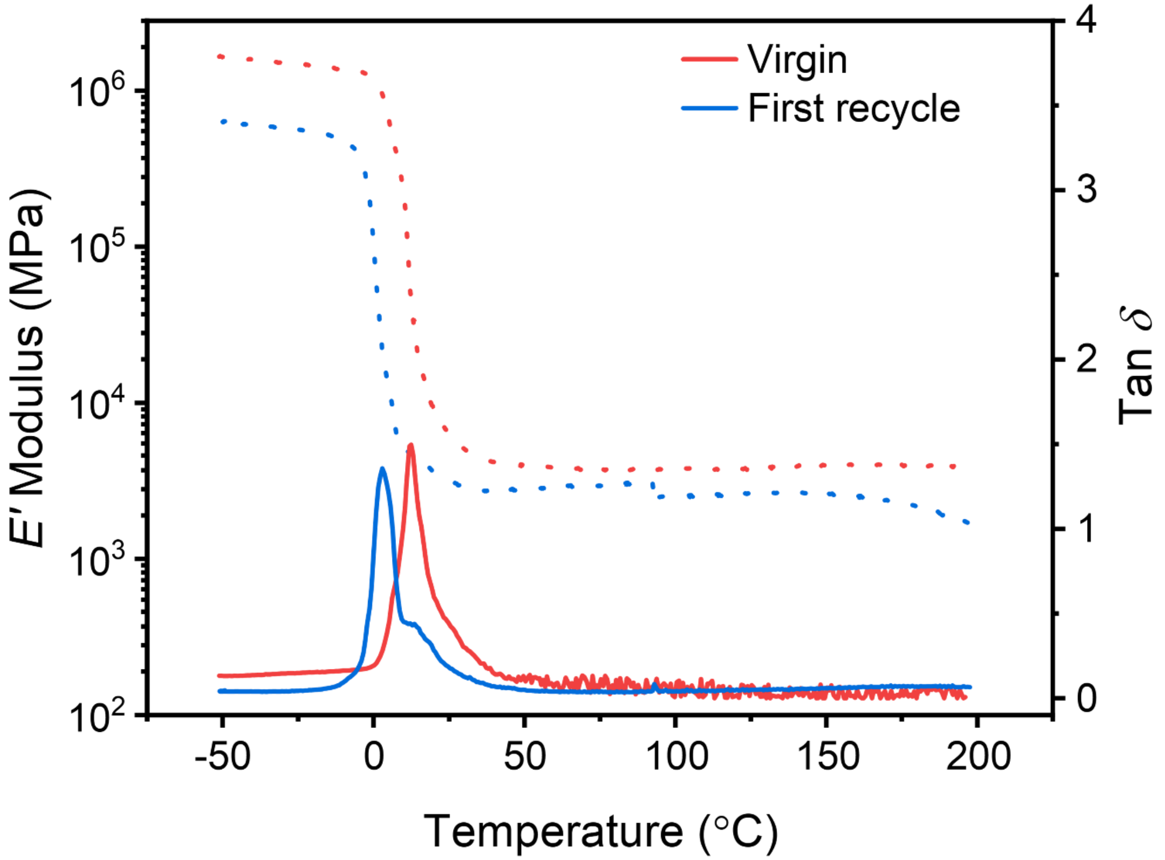

Fig. S64.
DMA temperature sweeps comparing recycled BPreT network to the virgin material in a temperature range from -50 to 200 °C at 5 °C min^{-1}.

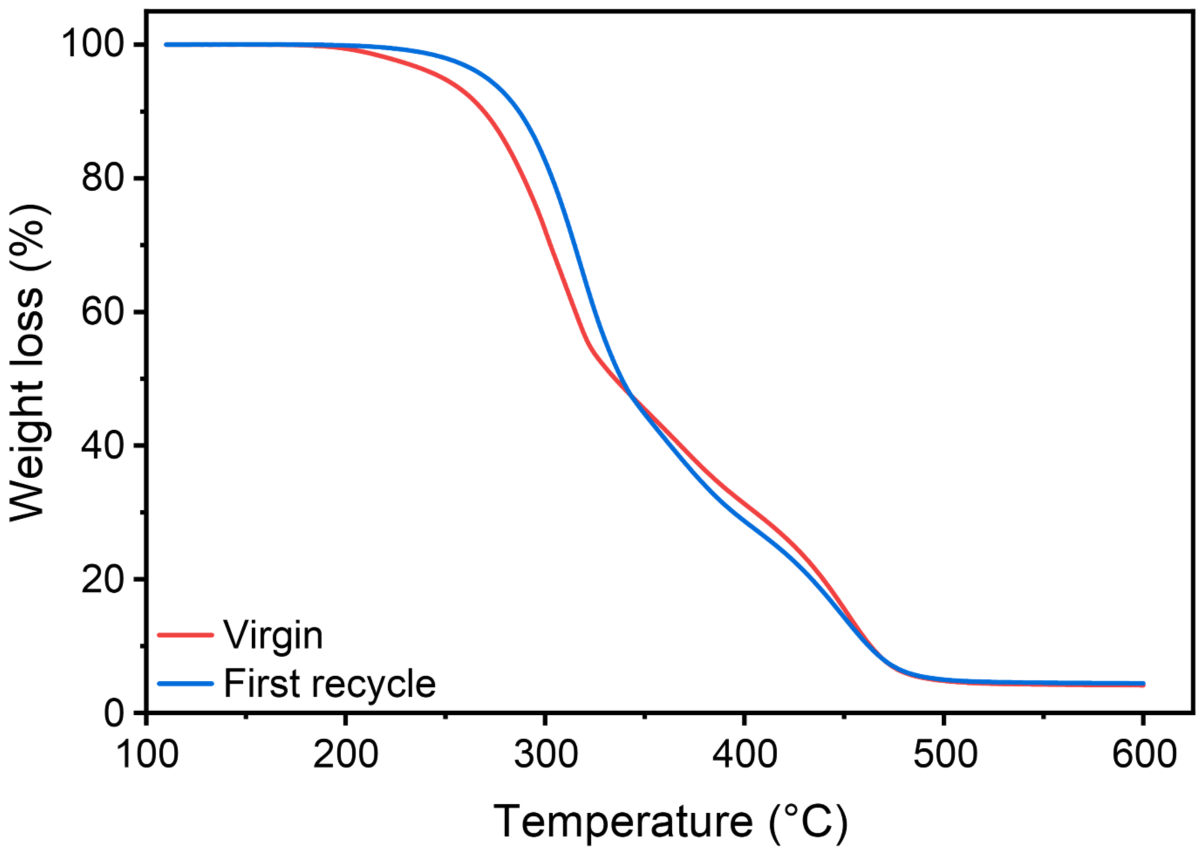

Fig. S65.
TGA thermograms comparing recycled BPreT network to the virgin material from 110 to 600 °C, heating rate 10 °C min^{-1}.

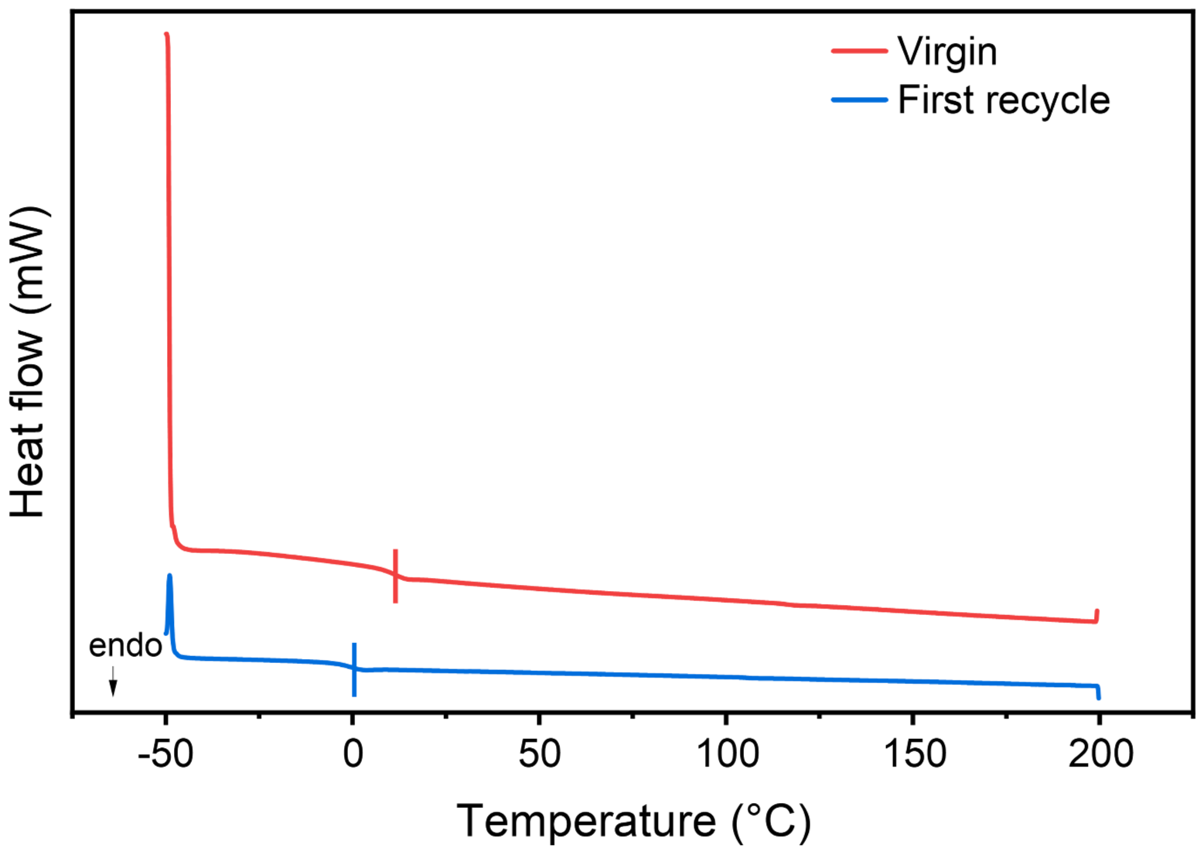

Fig. S66.
DSC thermograms of the second heating cycle comparing recycled BPreT network to the virgin material from –50 to 200 °C, heating rate 10 °C min^{-1}, with the T_g indicated by the vertical lines.

Python Code for automated HPLC analysis

```
#!/usr/bin/env python

import os
import numpy as np
import pandas as pd
import matplotlib.pylab as plt
import seaborn as sns
from scipy.signal import find_peaks
from hplc.io import load_chromatogram
from hplc.quant import Chromatogram
from colorama import Fore, Back, Style, init

#load the 3D data from the csv file as a dataframe
def load_HPLC_3D_Data (filename):
    print(f'\033[31m Now Processing {filename}. Hold Tight!\n')
    #loads the data
    data = pd.read_csv(f'{filename}.csv', sep=',', header=78, index_col=0)
    column_headings = list(data.columns)
    column_headings_nm = []
    for x in column_headings:
        x = x[:3] + '.' + x[3:]
        column_headings_nm.append(x)
    data.columns = column_headings_nm
    #transpose dataframe so Tr is x axis
    data_T = data.T

    #Replace column heading (x axis) with shortened values
    column_headings_T = list(data_T.columns)
    column_headings_T_min = []
    for x in column_headings_T:
        x = round(x, 2)
        column_headings_T_min.append(x)
    data_T.columns = column_headings_T_min

    #get sample name
    metadata_header = pd.read_csv(f'{filename}.csv', sep=',', header=0, index_col=0,
on_bad_lines='skip')
    sample_name=list(metadata_header.loc['Sample Name'])
    sample_name=str(sample_name[0])
    print(f'The sample in {filename} is called {sample_name}')
    print(f'\033[0m Loaded PDA Data for {filename} successfully')
    return(data, data_T, sample_name)

#retrieve a list of .csv files from a directory
```

```
def get_filenames(directory_in_str, filetype):
    directory = os.fsencode(directory_in_str)
    filelist = []
    filelist_with_extensions = []
    for file in os.listdir(directory):
        filename = os.fsdecode(file)
        if filename.endswith(filetype):
            # print(os.path.join(directory, filename))
            filelist_with_extensions.append(filename)
            filename_noext = os.path.splitext(filename)[0]
            filelist.append(filename_noext)
            continue
        else:
            continue
    return(filelist, filelist_with_extensions)

#extract HPLC traces from dataframe
def extract_HPLC_trace(dataframe, wavelength, filename):
    HPLC_trace_wavelength = dataframe[wavelength]
    HPLC_trace_wavelength.rename_axis(index='Tr', inplace=True)
    print(f'HPLC Chromatogram at {wavelength}nm successfully extracted')
    HPLC_trace_wavelength.rename(f'{filename}_{wavelength}nm')

HPLC_trace_wavelength.to_excel(f'{filename}/{filename}_Chromatogram_{wavelength}nm.xl
sx')

HPLC_trace_wavelength.to_csv(f'{filename}/{filename}_Chromatogram_{wavelength}nm.csv',
sep=',', index=True)
    print(f'Chromatogram for UV signal at {wavelength}nm data successfully saved')
    return(HPLC_trace_wavelength)

#plot and save a chromatogram at the specified wavelength
def plot_HPLC_Chromatogram (HPLC_trace_wavelength, wavelength, filename):
    #create the plot and specify the size
    fig, ax = plt.subplots(figsize=(15, 5))
    #modify the axis to be nice - create x and y axis labels
    #yticks = np.linspace(200, 400, 5)
    #xticks = [0,5,10,15,20,25,30,35,40,45,50,55,60]
    #plot the graph
    plt.plot(HPLC_trace_wavelength)
    #plt.xticks(xticks)
    #plt.yticks(yticks)
    plt.xlabel('Retention Time (min)')
    plt.ylabel('Absorbance')
    plt.savefig(f'{filename}/{filename}_Chromatogram_{wavelength}nm.png', dpi=600,
transparent=True)
```

```
    plt.close()
    print(f'Chromatogram for UV signal at {wavelength}nm successfully plotted')
    #plt.show()

#plot UV spectrum for a specific peak
def plot_UV_spectrum(UV_trace, UV_wavelengths, filename, peak_name):
    xticks = np.linspace(200, 400, 5)
    fig, ax = plt.subplots(figsize=(10, 10))
    UV_wavelengths = np.array(UV_wavelengths, dtype=np.float32)
    plt.plot(UV_wavelengths, UV_trace)
    plt.xticks(ticks=xticks, labels=xticks)
    plt.xlabel('Wavelength (nm)')
    plt.ylabel('Absorbance')
    plt.savefig(f'{filename}/{filename}_285nm_Tr{peak_name}_UV-spectrum.png', dpi=600,
transparent=True)
    plt.close()
    print(f'UV Spectrum for peak at Tr={peak_name} successfully plotted')
    return()


#find peaks from HPLC trace
def pick_chromatogram_peaks(HPLC_Trace, UV_wavelength, filename):
    peak_integer_index, peak_properties = find_peaks(HPLC_Trace, threshold=(70, None))
    #print(peak_integer_index)
    #Extract retention time from peak index
    peak_retention_time = []
    peak_maxima = []
    for x in peak_integer_index:
        peak_retention_time.append(HPLC_Trace.index[x])
        peak_maxima.append(HPLC_Trace.iloc[x])
    print('Peaks were detected at the following retention times:')
    print(list(map(float, peak_retention_time)))
    print('These peaks had the following maxima:')
    print(list(map(float, peak_maxima)))
    print('-------')
    #plot chromatogram with picked peaks labeled
    fig, ax = plt.subplots(figsize=(15, 5))
    plt.plot(HPLC_Trace)
    plt.plot(peak_retention_time, peak_maxima, "x")
    plt.xlabel('Retention Time (min)')
    plt.ylabel('Absorbance')
    plt.savefig(f'{filename}/{filename}_Chromatogram_{UV_wavelength}nm_Peaks-
Picked.png', dpi=600, transparent=True)
    plt.close()
    #plt.show()
    return(peak_integer_index, peak_retention_time, peak_maxima)
```

```
#extract and plot UV spectrum from major peaks
def extract_plot_UV_spectrum(Loaded_HPLC_Data, Loaded_HPLC_Data_T,
peak_retention_time, filename):
    #extract and plot the UV spectrum of each peak
    #crop peaks to 2dp so they match UV column headings
    peak_retention_time_min = []
    for x in peak_retention_time:
        x = round(x, 2)
        peak_retention_time_min.append(x)
    #extract the UV spectrum of each peak and plot as a graph
    UV_spectra_table = []
    UV_spectra_wavelengths = list(Loaded_HPLC_Data.columns)
    print(f'The PDA signal ranges from {UV_spectra_wavelengths[0]}nm to
{UV_spectra_wavelengths[-1]}nm')
    for x in peak_retention_time_min:
        UV_spectra_table.append(list(Loaded_HPLC_Data_T[x]))
        plot_UV_spectrum(Loaded_HPLC_Data_T[x], UV_spectra_wavelengths, filename, x)
    #Output these arrayed UV spectra as a data table
    UV_spectra_table = pd.DataFrame(UV_spectra_table)
    UV_spectra_table = UV_spectra_table.T
    UV_spectra_table.columns=peak_retention_time_min
    UV_spectra_table.index=UV_spectra_wavelengths
    print('-------')
    print('The detected peaks and associated UV spectra are as follows:')
    print(UV_spectra_table)
    UV_spectra_table.to_excel(f'{filename}/{filename}_Arrayed_Peak_UV_Spectra.xlsx')
    UV_spectra_table.to_csv(f'{filename}/{filename}_Arrayed_Peak_UV_Spectra.csv', sep=',',
index=True)
    return(UV_spectra_table)

#process HPLC chromatogram using hplc-py
#load chromatogram
def hplc_py_processing(wavelength, filename):
    print(f'HPLC-py processing for {filename} at {wavelength}nm in progress...')
    loaded_chromatogram =
load_chromatogram(f'{filename}/{filename}_Chromatogram_{wavelength}nm.csv',
cols={'Tr':'time', f'{wavelength}': 'signal'})
    #convert to chromatogram object
    chrom = Chromatogram(loaded_chromatogram)
    #correct baseline
    chrom.correct_baseline(window=2)
    #fit peaks
    peaks = None
    try:
```

```
        peaks = chrom.fit_peaks(buffer=1, prominence=0.15, param_bounds={'amplitude':[0.001,
100], 'scale':[0, np.inf]}, known_peaks={
                                                    19.0 : {'width': 0.25},
                                                    20.0 : {'width': 0.25},
                                                    31.0 : {'width': 0.25}}) #add trimer and tetramer
and TPA to this
        #peaks = chrom.fit_peaks(buffer=50, prominence=0.02)
    except ValueError as error:
        print(f'\033[1;33m {error}\n\033[0m')
        print(f'Ohh dear bud, looks like HPLC-py cannot detect the peaks in {filename} at
{wavelength}nm. Trying agan with looser parameter bounds... This might take a while, why not
head to Timmy\'s?')
        try:
            peaks = chrom.fit_peaks(buffer=1, prominence=0.02,
param_bounds={'amplitude':[0.001, 100], 'scale':[0, np.inf]}, known_peaks={
                                                    19.0 : {'width': 0.25},
                                                    20.0 : {'width': 0.25},
                                                    31.0 : {'width': 0.25}})
        except ValueError as error:
            print(f'\033[1;33m {error}\n\033[0m')
            print('nope, HPLC-py just doesn\'t like this trace. Giving up for now...')
        #assess the fit of the peaks
        if peaks != None:
            try:
                chrom.assess_fit()
                print('-------')
            except AttributeError as error:
                print(f'\033[1;33m {error}\n\033[0m')
                pass
    print('The detected peaks and integrations are as follows:')
    print(peaks)
    print('-------')
        #save the data
    try:
        peaks.to_excel(f'{filename}/{filename}_HPLC-py_Fitted-Peaks_{wavelength}nm.xlsx')
        peaks.to_csv(f'{filename}/{filename}_HPLC-py_Fitted-Peaks_{wavelength}nm.csv',
sep=',', index=True)
    except AttributeError as error:
        print(f'\033[1;33m {error}\n\033[0m')
        pass

    #Define the peaks of interest and their calibration curves for quantitation
    if wavelength == '284.75':
        #ADD TPA, Trimer, and Tetramer here!!
        HPLC_calibration = {'MHET': {'retention_time': 19.0, 'slope': 3000000, 'intercept': -
223727, 'unit': 'mM'}, #uses universal calibration from BHET and Dimer, R² = 0.998
```

```
                            'BHET': {'retention_time': 20.0, 'slope': 3000000, 'intercept': -223727, 'unit':
'mM'}, #uses universal calibration from BHET and Dimer, R² = 0.998
                            'Dimer': {'retention_time': 31.0, 'slope': 3000000, 'intercept': -223727, 'unit':
'mM'}} #uses universal calibration from BHET and Dimer, R² = 0.998
        try:
            quantitated_peaks = chrom.map_peaks(HPLC_calibration, loc_tolerance=0.2)
            print('According to the calibration, the following species were detected in the HPLC
injection vial:')
            print(quantitated_peaks)
            print('-------')
        except AttributeError as error:
            print(f'\033[1;33m {error}\n\033[0m')
            print('Unfortunately the quantitation failed, trying with stricter bounds.')
            try:
                #ADD TPA, Trimer, and Tetramer here too!!
                HPLC_calibration = {'MHET': {'retention_time': 19.0, 'slope': 3000000, 'intercept': -
223727, 'unit': 'mM'}, #uses universal calibration from BHET and Dimer, R² = 0.998
                                    'BHET': {'retention_time': 20.0, 'slope': 3000000, 'intercept': -223727,
'unit': 'mM'}, #uses universal calibration from BHET and Dimer, R² = 0.998
                                    'Dimer': {'retention_time': 31.0, 'slope': 3000000, 'intercept': -223727,
'unit': 'mM'}} #uses universal calibration from BHET and Dimer, R² = 0.998
                quantitated_peaks = chrom.map_peaks(HPLC_calibration, loc_tolerance=0.1)
                print('According to the calibration, the following species were detected in the HPLC
injection vial:')
                print(quantitated_peaks)
                print('-------')
            except AttributeError as error:
                print(f'\033[1;33m {error}\n\033[0m')
                print('Sorry bud, the quantitation failed for this sample. But you can try quantitating
manually from the peak integrations')
                pass
        #save the quantitated peaks data
        try:
            quantitated_peaks.to_excel(f'{filename}/{filename}_HPLC-py_Fitted-
Peaks_{wavelength}nm_Quantitated.xlsx')
            quantitated_peaks.to_csv(f'{filename}/{filename}_HPLC-py_Fitted-
Peaks_{wavelength}nm_Quantitated.csv', sep=',', index=True)
        except (AttributeError, UnboundLocalError) as error:
            print(f'\033[1;33m {error}\n\033[0m')
            pass

    #save chromatogram
    fig, ax = chrom.show()
    ax.legend()
    plt.savefig(f'{filename}/{filename}_HPLC-py_Chromatogram_Fitted_{wavelength}nm.png',
dpi=600, transparent=False)
```

```
    plt.close()
    plt.style.use('default')
    print(f'HPLC-py processing complete for {filename}.')
    print('-------')
    return(peaks)

#Process all .csv files in the current directory
def process_HPLC_data_from_filelist(filelist):
    #load a HPLC dataset
    for x in filelist:
        filename = x
        Loaded_HPLC_Data, Loaded_HPLC_Data_T, Loaded_HPLC_Data_SampleName =
load_HPLC_3D_Data(filename)
        #create a subfolder to save data
        try:
            os.mkdir(f'{Loaded_HPLC_Data_SampleName}') #creating a subfolder
        except FileExistsError: #if subfolder already exists
            pass
        #save 3D data as table

Loaded_HPLC_Data_T.to_excel(f'{Loaded_HPLC_Data_SampleName}/{Loaded_HPLC_Data
_SampleName}_PDA_Data.xlsx')

Loaded_HPLC_Data_T.to_csv(f'{Loaded_HPLC_Data_SampleName}/{Loaded_HPLC_Data_S
ampleName}_PDA_Data.csv', sep=',', index=True)
        # #print(Loaded_HPLC_Data)
        # #print(list(Loaded_HPLC_Data.columns))
        #----------------------

        #plot and save a 2D heatmap of the 3D PDA signal
        #create the plot and specify the size
        fig, ax = plt.subplots(figsize=(15, 5))
        #modify the axis to be nice - create x and y axis labels
        yticks = np.linspace(200, 400, 5)
        xticks = [0,10,20,30,40,50,60]
        #plot the graph
        plt.imshow(Loaded_HPLC_Data_T, cmap='viridis', interpolation='nearest',
extent=[0,60,400,200], aspect=0.1)
        plt.xticks(xticks)
        plt.yticks(yticks)
        plt.xlabel('Retention Time (min)')
        plt.ylabel('Wavelength (nm)')

plt.savefig(f'{Loaded_HPLC_Data_SampleName}/{Loaded_HPLC_Data_SampleName}_PDA-
Plot.png', dpi=600, transparent=True)
        plt.close()
```

```
        print('2D heatmap of the 3D PDA Data successfully plotted')
        print('-------')
        #plt.show()
        #----------------------

        #Extract and plot the chromatogram from specific wavelengths
        #Extract the chromatogram from specific wavelengths
        wavelength_to_plot_1 = '284.75'
        Trace_285nm = extract_HPLC_trace(Loaded_HPLC_Data, wavelength_to_plot_1,
Loaded_HPLC_Data_SampleName)
        plot_HPLC_Chromatogram (Trace_285nm, wavelength_to_plot_1,
Loaded_HPLC_Data_SampleName)
        wavelength_to_plot_2 = '259.63'
        Trace_260nm = extract_HPLC_trace(Loaded_HPLC_Data, wavelength_to_plot_2,
Loaded_HPLC_Data_SampleName)
        plot_HPLC_Chromatogram (Trace_260nm, wavelength_to_plot_2,
Loaded_HPLC_Data_SampleName)
        print('-------')
        #----------------------

        #extract and plot UV spectrum from major peaks
        #find peaks from HPLC trace
        peak_integer_index, peak_retention_time, peak_maxima =
pick_chromatogram_peaks(Trace_285nm, wavelength_to_plot_1,
Loaded_HPLC_Data_SampleName)
        UV_spectra_table = extract_plot_UV_spectrum(Loaded_HPLC_Data,
Loaded_HPLC_Data_T, peak_retention_time, Loaded_HPLC_Data_SampleName)
        print('-------')
        #----------------------

        #process HPLC chromatogram at 285nm and 260nm using hplc-py
        try:
            HPLCpy_Peaks_1 = hplc_py_processing(wavelength_to_plot_1,
Loaded_HPLC_Data_SampleName)
        except ValueError as error:
            print(f'\033[1;33m {error}\n\033[0m')
            print(f'Sorry pal, HPLC-py did not like {Loaded_HPLC_Data_SampleName} at
{wavelength_to_plot_1}nm. The chromatogram trace shape is not compatible with HPLC-py.')
        try:
            HPLCpy_Peaks_2 = hplc_py_processing(wavelength_to_plot_2,
Loaded_HPLC_Data_SampleName)
        except ValueError as error:
            print(f'\033[1;33m {error}\n\033[0m')
            print(f'Sorry pal, HPLC-py did not like {Loaded_HPLC_Data_SampleName} at
{wavelength_to_plot_2}nm. The chromatogram trace shape is not compatible with HPLC-py.')
```

```
    #sample complete
    print(f'Data processing complete for {Loaded_HPLC_Data_SampleName}. HOORAY!!
=)')
    continue


#-----------------------


#get a list of the filenames
directory_in_str = os.getcwd()
fileformat = '.csv'
#get a list of .csv files in the current directory
filelist_csv_noext, filelist_csv_ext = get_filenames(directory_in_str, f'{fileformat}')

#inform the user of the current directory and .csvf files within
print(f'The working folder is: \n {directory_in_str}')
print('-------')
print(f'The following {fileformat} files are in the working folder: \n {filelist_csv_ext}')
print('-------')
#-----------------------

#Process all .csv files in the current directory
process_HPLC_data_from_filelist(filelist_csv_noext)

#Complete
print('Data processing complete for all HPLC samples in this folder. Have a nice day buddy!')
os.system("pause")
```

REFERENCES

1. R. Geyer, J. R. Jambeck, K. L. Law, Production, use, and fate of all plastics ever made. *Sci. Adv.* **3**, e1700782 (2017).

2. M. Babaei, M. Jalilian, K. Shahbaz, Chemical recycling of polyethylene terephthalate: A mini-review. *J. Environ. Chem. Eng.* **12**, 112507 (2024).

3. E. Barnard, J. J. Rubio Arias, W. Thielemans, Chemolytic depolymerisation of PET: A review. *Green Chem.* **23**, 3765–3789 (2021).

4. V. Sinha, M. R. Patel, J. V. Patel, PET waste management by chemical recycling: A review. *J. Polym. Environ.* **18**, 8–25 (2010).

5. L. T. Korley, T. H. Epps III, B. A. Helms, A. J. Ryan, Toward polymer upcycling—Adding value and tackling circularity. *Science* **373**, 66–69 (2021).

6. S. C. Kosloski-Oh, Z. A. Wood, Y. Manjarrez, J. P. de Los Rios, M. E. Fieser, Catalytic methods for chemical recycling or upcycling of commercial polymers. *Mater. Horiz.* **8**, 1084–1129 (2021).

7. S. E. Lewis, B. E. Wilhelmy, F. A. Leibfarth, Organocatalytic C–H fluoroalkylation of commodity polymers. *Polym. Chem.* **11**, 4914–4919 (2020).

8. C. Jehanno, J. W. Alty, M. Roosen, S. De Meester, A. P. Dove, E. Y.-X. Chen, F. A. Leibfarth, H. Sardon, Critical advances and future opportunities in upcycling commodity polymers. *Nature* **603**, 803–814 (2022).

9. J. C. Worch, A. P. Dove, 100th anniversary of macromolecular science viewpoint: Toward catalytic chemical recycling of waste (and future) plastics. *ACS Macro Lett.* **9**, 1494–1506 (2020).

10. G. W. Coates, Y. D. Getzler, Chemical recycling to monomer for an ideal, circular polymer economy. *Nat. Rev. Mater.* **5**, 501–516 (2020).

11. J. P. Tan, J. Tan, N. Park, K. Xu, E. D. Chan, C. Yang, V. A. Piunova, Z. Ji, A. Lim, J. Shao, Upcycling poly (ethylene terephthalate) refuse to advanced therapeutics for the treatment of nosocomial and mycobacterial infections. *Macromolecules* **52**, 7878–7885 (2019).

12. M. Poderyte, R. Lima, P. I. Golbækdal, D. W. Juhl, K. L. Olesen, N. C. Nielsen, A. Lanza, J.-W. Lee, Repurposing polyethylene terephthalate plastic waste to capture carbon dioxide. *Sci. Adv.* **11**, eadv5906 (2025).

13. K. Fukushima, J. M. Lecuyer, D. S. Wei, H. W. Horn, G. O. Jones, H. A. Al-Megren, A. M. Alabdulrahman, F. D. Alsewailem, M. A. McNeil, J. E. Rice, Advanced chemical recycling of poly (ethylene terephthalate) through organocatalytic aminolysis. *Polym. Chem.* **4**, 1610–1616 (2013).

14. J. Demarteau, I. Olazabal, C. Jehanno, H. Sardon, Aminolytic upcycling of poly (ethylene terephthalate) wastes using a thermally-stable organocatalyst. *Polym. Chem.* **11**, 4875–4882 (2020).

15. G. Mir Mohamad Sadeghi, R. Shamsi, M. Sayaf, From aminolysis product of PET waste to novel biodegradable polyurethanes. *J. Polym. Environ.* **19**, 522–534 (2011).

16. T. Spychaj, E. Fabrycy, S. Spychaj, M. Kacperski, Aminolysis and aminoglycolysis of waste poly (ethylene terephthalate). *J. Mater. Cycles Waste Manag.* **3**, 24–31 (2001).

17. E. Bulak, I. Acar, The use of aminolysis, aminoglycolysis, and simultaneous aminolysis–hydrolysis products of waste PET for production of paint binder. *Polym. Eng. Sci.* **54**, 2272–2281 (2014).

18. J. He, J. W. Kim, K. Yamaguchi, N. Mizuno, Efficient catalytic synthesis of tertiary and secondary amines from alcohols and urea. *ChemInform* **41**, 9888–9891 (2010).

19. C. Gunanathan, D. Milstein, Selective synthesis of primary amines directly from alcohols and ammonia. *Angew. Chem. Int. Ed.* **47**, 8661–8664 (2008).

20. M. Shiramizu, F. D. Toste, Deoxygenation of biomass-derived feedstocks: Oxorhenium-catalyzed deoxydehydration of sugars and sugar alcohols. *Angew. Chem. Int. Ed.* **51**, 8082–8086 (2012).

21. D. Sun, S. Sato, W. Ueda, A. Primo, H. Garcia, A. Corma, Production of C4 and C5 alcohols from biomass-derived materials. *Green Chem.* **18**, 2579–2597 (2016).

22. P. Roose, K. Eller, E. Henkes, R. Rossbacher, H. Höke, "Aliphatic amines" in *Ullmann's Encyclopedia of Industrial Chemistry*, B. Elvers, S. Hawkins, G. Schulz, Eds. (VCH, Weinheim, ed. 5, 1985), vol. A2, pp. 1–55.

23. O. Strubelt, M. Deters, R. Pentz, C. Siegers, M. Younes, The toxic and metabolic effects of 23 aliphatic alcohols in the isolated perfused rat liver. *Toxicol. Sci.* **49**, 133–142 (1999).

24. C. C. Westover, T. E. Long, Envisioning a BHET economy: Adding value to PET waste. *Sustain. Chem.* **4**, 363–393 (2023).

25. R. López-Fonseca, I. Duque-Ingunza, B. De Rivas, S. Arnaiz, J. I. Gutierrez-Ortiz, Chemical recycling of post-consumer PET wastes by glycolysis in the presence of metal salts. *Polym. Degrad. Stab.* **95**, 1022–1028 (2010).

26. S. Baliga, W. T. Wong, Depolymerization of poly (ethylene terephthalate) recycled from post-consumer soft-drink bottles. *J. Polym. Sci. A* **27**, 2071–2082 (1989).

27. I. Olazabal, E. J. Luna Barrios, S. De Meester, C. Jehanno, H. Sardon, Overcoming the limitations of organocatalyzed glycolysis of poly (ethylene terephthalate) to facilitate the recycling of complex waste under mild conditions. *ACS Appl. Polym. Mater.* **6**, 4226–4232 (2024).

28. N. H. Le, T. T. Ngoc Van, B. Shong, J. Cho, Low-temperature glycolysis of polyethylene terephthalate. *ACS Sustain. Chem. Eng.* **10**, 17261–17273 (2022).

29. E. Luna, I. Olazabal, M. Roosen, A. Müller, C. Jehanno, M. Ximenis, S. De Meester, H. Sardon, Towards a better understanding of the cosolvent effect on the low-temperature glycolysis of polyethylene terephthalate (PET). *Chem. Eng. J.* **482**, 148861 (2024).

30. T. Sako, T. Sugeta, K. Otake, N. Nakazawa, M. Sato, K. Namiki, M. Tsugumi, Depolymerization of polyethylene terephthalate to monomers with supercritical methanol. *J. Chem. Eng. Jpn.* **30**, 342–346 (1997).

31. D. D. Pham, J. Cho, Low-energy catalytic methanolysis of poly (ethyleneterephthalate). *Green Chem.* **23**, 511–525 (2021).

32. K. Fukushima, D. J. Coady, G. O. Jones, H. A. Almegren, A. M. Alabdulrahman, F. D. Alsewailem, H. W. Horn, J. E. Rice, J. L. Hedrick, Unexpected efficiency of cyclic amidine catalysts in depolymerizing poly (ethylene terephthalate). *J. Polym. Sci. A* **51**, 1606–1611 (2013).

33. S. Liu, Z. Wang, L. Li, S. Yu, C. Xie, F. Liu, Butanol alcoholysis reaction of polyethylene terephthalate using acidic ionic liquid as catalyst. *J. Appl. Polym. Sci.* **130**, 1840–1844 (2013).

34. K. R. Delle Chiaie, F. R. McMahon, E. J. Williams, M. J. Price, A. P. Dove, Dual-catalytic depolymerization of polyethylene terephthalate (PET). *Polym. Chem.* **11**, 1450–1453 (2020).

35. F. Chen, Q. Zhou, R. Bu, F. Yang, W. Li, Kinetics of poly (ethylene terephthalate) fiber glycolysis in ethylene glycol. *Fiber Polym.* **16**, 1213–1219 (2015).

36. J. Sutton, G. Grause, A. A. R. Hmayed, S. T. Street, A. P. Dove, J. Wood, Organocatalytic glycolysis of polyethylene terephthalate and product separation by membrane filtration. *Chem. Eng. J.* **512**, 162400 (2025).

37. F. F. Chen, G. H. Wang, W. Li, F. Yang, Kinetics of glycolysis of poly (ethylene terephthalate) by shrinking-core model. *Adv. Mater. Res.* **233**, 627–631 (2011).

38. J. W. Chen, L. W. Chen, W. H. Cheng, Kinetics of glycolysis of polyethylene terephthalate with zinc catalyst. *Polym. Int.* **48**, 885–888 (1999).

39. S. Kaiho, A. A. R. Hmayed, K. R. Delle Chiaie, J. C. Worch, A. P. Dove, Designing thermally stable organocatalysts for poly (ethylene terephthalate) synthesis: Toward a one-pot, closed-loop chemical recycling system for PET. *Macromolecules* **55**, 10628–10639 (2022).

40. A. J. Spicer, A. Brandolese, A. P. Dove, Selective and sequential catalytic chemical depolymerization and upcycling of mixed plastics. *ACS Macro Lett.* **13**, 189–194 (2024).

41. J. Huang, D. Yan, Q. Zhu, X. Cheng, J. Tang, X. Lu, J. Xin, Depolymerization of polyethylene terephthalate with glycol under comparatively mild conditions. *Polym. Degrad. Stab.* **208**, 110245 (2023).

42. K. Van Aken, L. Strekowski, L. Patiny, EcoScale, a semi-quantitative tool to select an organic preparation based on economical and ecological parameters. *Beilstein J. Org. Chem.* **2**, 3 (2006).

43. A. Gałuszka, Z. M. Migaszewski, P. Konieczka, J. Namieśnik, Analytical Eco-Scale for assessing the greenness of analytical procedures. *Trends Anal. Chem.* **37**, 61–72 (2012).

44. Y. Suzuki, T. Kano, T. Tomii, N. Tsuji, A. Matsumoto, Relaxation and amorphous structure of polymers containing rigid fumarate segments. *Polymers* **14**, 4876–4887 (2022).

45. M. Sepe, *Dynamic Mechanical Analysis for Plastics Engineering* (William Andrew, 1998).

46. T. R. Long, R. M. Elder, E. D. Bain, K. A. Masser, T. W. Sirk, J. H. Yu, D. B. Knorr, J. L. Lenhart, Influence of molecular weight between crosslinks on the mechanical properties of polymers formed via ring-opening metathesis. *Soft Matter* **14**, 3344–3360 (2018).

47. A. Oussai, Z. Bártfai, L. Kátai, Development of 3D printing raw materials from plastic waste. A case study on recycled polyethylene terephthalate. *Appl. Sci.* **11**, 7338 (2021).

48. T. Kuhnt, F. L. Morgan, M. B. Baker, L. Moroni, An efficient and easily adjustable heating stage for digital light processing set-ups. *Addit. Manuf.* **46**, 102102 (2021).

49. Y. He, N. Li, Z. Xiang, Y. Rong, L. Zhu, X. Huang, Natural polyphenol as radical inhibitors used for DLP-based 3D printing of photosensitive gels. *Mater. Today Commun.* **33**, 104698 (2022).

50. T. M. Lammens, M. C. R. Franssen, E. L. Scott, J. P. M. Sanders, Synthesis of biobased N-methylpyrrolidone by one-pot cyclization and methylation of γ-aminobutyric acid. *Green Chem.* **12**, 1430–1436 (2010).

51. O. Clavilier, D. Foy, F. Byrne, The solvent miscibility table updated: Miscibility and potential applications of green solvents. *Green Chem.* **27**, 12151–12159 (2025).

52. J. W. Seo, G. M. Kim, Y. Choi, J. M. Cha, H. Bae, Improving printability of digital-light-processing 3D bioprinting via photoabsorber pigment adjustment. *Int. J. Mol. Sci.* **23**, 5428 (2022).

53. Z. Wang, Q. Lu, X. Li, Y. Zhou, Y. Xiao, M. Lang, Digital light processing of customized elastic scaffolds by efficient thiol-yne crosslinking. *Eur. Polym. J.* **202**, 112586 (2024).

54. G. Chure, J. Cremer, hplc-py: A python utility for rapid quantification of complex chemical chromatograms. *J. Open Source Softw.* **9**, 6270 (2024).

55. D. Tillier, H. Lefebvre, M. Tessier, J. C. Blais, A. Fradet, High temperature bulk reaction between poly (ethylene terephtalate) and lactones: 1H NMR and SEC/MALDI-TOF MS study. *Macromol. Chem. Phys.* **205**, 581–592 (2004).

56. Ecoscale calculator (2006); https://ecoscale.cheminfo.org/calculator.
